# Boring bryozoans: an investigation into the endolithic bryozoan family Penetrantiidae

**DOI:** 10.1007/s13127-023-00612-z

**Published:** 2023-05-24

**Authors:** Sebastian H. Decker, Masato Hirose, Sarah Lemer, Piotr Kuklinski, Hamish G. Spencer, Abigail M. Smith, Thomas Schwaha

**Affiliations:** 1 Department of Evolutionary Biology, University of Vienna, Schlachthausgasse 43, 1030 Vienna, Austria; 2 School of Marine Biosciences, Kitasato University, Kitasato 1-15-1, Sagamihara-Minami, Kanagawa, 252-0373 Japan; 3 Marine Laboratory, UOG Station, Mangilao, Guam 96923 USA; 4 Institute of Oceanology, Polish Academy of Sciences, Sopot, Poland; 5 Department of Zoology, University of Otago, Dunedin, New Zealand; 6 Department of Marine Science, University of Otago, Dunedin, New Zealand

**Keywords:** Ctenostomata, *Penetrantia*, Microeroders, Gonozooid

## Abstract

An endolithic lifestyle in mineralized substrates has evolved multiple times in various phyla including Bryozoa. The family Penetrantiidae includes one genus with ten extant and two fossil species. They predominantly colonize the shells of molluscs and establish colonies by chemical dissolution of calcium carbonate. Based on several morphological characters, they were described to be either cheilostome or ctenostome bryozoans. For more than 40 years, neither the characters of species identity and systematics nor the problem of their phylogeny was approached. Consequently, the aim of this study is to reevaluate species identities and the systematic position of the genus *Penetrantia* by analyzing at least six different species from eight regions with the aid of modern methods such as confocal laser scanning microscopy and 3D-reconstruction techniques. This study demonstrates that the musculature associated with the operculum and brood chamber shows significant differences from the cheilostome counterparts and seems to have evolved independently. Together with the presence of other ctenostome-like features such as true polymorphic stolons and uncalcified body wall, this finding supports a ctenostome affinity. Operculum morphology reveals many new species-specific characters, which, together with information about gonozooid morphology, tentacle number, and zooid size ranges, will enhance species identification. It also revealed a probable new species in Japan as well as potential cryptic species in France and New Zealand. In addition, this study increases the known distribution range of the family and its substrate diversity. Altogether, the new information collated here provides the basis for future work on a neglected taxon.

**Supplementary Information:**

The online version contains supplementary material available at 10.1007/s13127-023-00612-z.

## Introduction

Bryozoa are a lophotrochozoan phylum which comprises exclusively aquatic and suspension feeding animals. Currently, there are almost 6000 recent and more than 15,000 fossil bryozoan species recognized (Bock & Gordon, 2013). The vast majority live in marine environments with only a small fraction being restricted to limnic or brackish habitats. Each colony is composed of repetitive and modular units, the zooids. Zooids can proliferate by asexual reproduction for colony growth and thereby colonize substrates as flat sheath-like encrusting colonies or as erect and highly structured colonies (Hageman et al., 1998; Ryland, 1970). All bryozoans show a characteristic, traditional division of zooids into a cystid and polypide. The cystid comprises the body wall, which is mineralized in the majority of bryozoan species. The polypide includes the u-shaped gut, the ciliated tentacle crown or lophophore, and associated organs.

Typical for all bryozoans is the retractability of the polypide into the cystid (e.g., Mukai et al., 1997). There are two clades of bryozoans present in marine environments: Stenolaemata and Gymnolaemata (Schwaha, 2020a). Stenolaemates are represented by one extant clade, Cyclostomata, with approximately 540 species, whereas the majority of recent bryozoans are gymnolaemates and most are part of the Cheilostomata clade. Ctenostomes are a small group of gymnolaemates with about 350 species, whereas over 5500 species are cheilostomes (Bock & Gordon, 2013; Schwaha, 2020a; Waeschenbach et al., 2012).

While most ctenostomes are tiny encrusters, a few have adopted an endolithic lifestyle and live within mineralized substrates. These endoliths are present in four recent ctenostome families (Terebriporidae, Spathiporidae, Immergentiidae, and Penetrantiidae). Current data indicate that they occur predominantly in the shells of living and dead molluscs (Pohowsky, 1978; Soule & Soule, 1969a). An additional nine boring bryozoan taxa are known from the fossil record, with the oldest specimen dating back to the Ordovician, indicating an early radiation and adaptation of an endolithic lifestyle in ctenostomes (Pohowsky, 1978). However, these fossil taxa represent trace fossils and thereby should be considered ichnotaxa rather than true biologic taxa, since they were erected based on their boring traces only without soft body information (Bertling et al., 2006; Rosso, 2008). The same issue accounts for the recent families Terebriporidae and Spathiporidae and is currently debated (Bertling et al., 2006). Although boring traces of boring bryozoans give detailed information about their colony morphologies (Pohowsky, 1974), they only truly resemble their bioerosion activities (Bertling et al., 2006; Wisshak et al., 2019).

Almost nothing is currently known about the phylogenetic relationships of recent boring bryozoans. Given its long history, together with morphological differences, a “boring” lifestyle has probably evolved several times independently within ctenostomes (Pohowsky, 1978). Most likely, endolithic bryozoans use chemical dissolution to bore into the substrate as mechanical means are not present/available (Pohowsky, 1974, 1978; Silén, 1947), but the process is still little understood.

All four recent boring bryozoan families have their colonies completely immersed in the substrate with only the boreholes’ apertures, and often parts of the interconnecting stolons or cystid appendages, visible as traces from the outside. The zooids are usually widely spaced and interconnected either by zooidal polymorphs, (i.e., kenozooidal stolons), or, in the case of the Immergentiidae, by a network of cystid appendages (“pseudostolons”), which do not represent true polymorphs (Pohowsky, 1978; Schwaha, 2020d; Silén, 1947).

The family Penetrantiidae currently includes ten extant and two fossil species within the sole genus *Penetrantia* (Pohowsky, 1978; Schwaha, 2020d). They were erected based on histological information of a recent species and is thereby an accepted biologic taxon (Bertling et al., 2006; Pohowsky, 1974; Silén, 1946). They are almost globally distributed, although the majority of species are present in tropical and subtropical intertidal habitats (Pohowsky, 1978; Schwaha, 2020d).

This family is perhaps the most studied of the boring bryozoans, in part because it has some features that other boring bryozoans lack: operculum, true polymorphic gonozooids with ovicell-like brood chambers, and a double cuticular lining of the body wall. The operculum and gonozooid morphology, in particular, serve as important characters in species identification (Pohowsky, 1978; Silén, 1946, 1947). The similarity of the operculum and the ovicell-like brood chambers to cheilostomes have fueled a long ongoing discussion about the affinity of penetrantiids to cheilostomes or ctenostomes (Pohowsky, 1978; Soule & Soule, 1969a, 1975).

The paucity of any detailed investigations of boring bryozoans including penetrantiids (Pohowsky, 1978) calls for more wholistic approaches to summarize our knowledge and moreover, to add new and comparative data in order to better understand this neglected taxon. Consequently, this study has several aims with respect to the family Penetrantiidae: (a) assess biogeographic distribution and substrate preferences; (b) thoroughly investigate morphology with modern methods in order to understand colonial, autozooidal, and gonozooidal structures; (c) evaluate morphological characters in light of the potential ctenostome affinity of penetrantiids; (d) assess characters for reliable species identification; and (e) reevaluate existing species’ characters and identities to reveal potential cryptic species and better understand penetrantiid diversity. For this purpose, we investigated a large collection of penetrantiids from eight different regions utilizing a variety of methods and compared our data with all information available in the literature.

## Material and methods

### Sample collection

*Penetrantia* specimens from 20 different localities in eight different regions were collected (Table 1). Additionally, a large amount of shell material from polar regions was checked for boring traces, including from the artic Svalbard, the Aleuten Islands in the Bering Sea and from Kerguelen Islands in the Southern Ocean. Collections were made by hand/diver in the intertidal and by dredges on the shelf.

**Table 1:**
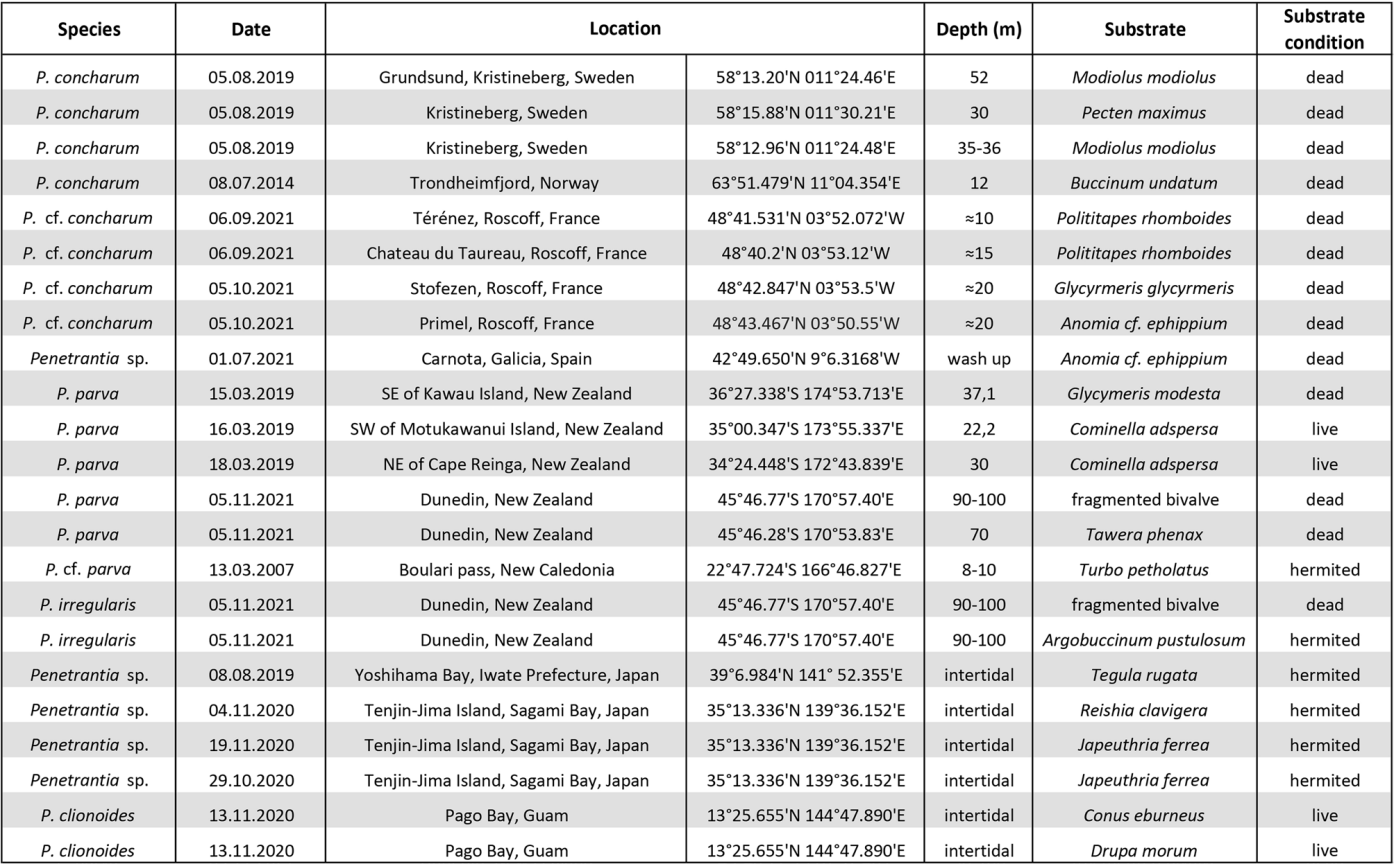
Summary of penetrantiid specimens used for morphological investigations in this study

### Biogeography and substrate diversity

Collection data of this study and all available data from the literature were combined to generate a distribution map using Khartis 2.1.0 (Sciences Po, Paris, France, https://www.sciencespo.fr/cartographie/khartis/) and Photoshop (Adobe Inc., San Jose, CA, USA).

### Morphological analysis

Stereomicroscopic images of shells were obtained using a Nikon SMZ25 stereomicroscope (Nikon, Tokyo, Japan) with a DsRi2 microscope camera, Nikon SMZ stereomicroscope combined with a Nikon Z6 mirrorless camera or with a Hirox RH-2000 3D digital microscope (Hirox Co., Ltd., Tokyo, Japan).

For histological sectioning, samples fixed in 2.5% glutaraldehyde or in 96% ethanol were used and infiltrated with Agar Low Viscosity Resin (LVR, Agar Scientific Ltd., Stansted, UK). Glutarfixed samples were sometimes postfixed in 1% aqueous osmium tetroxide prior to dehydration for resin embedding. Dehydration was conducted via a graded ethanol series, and resin infiltration was carried out in an acetone series where the concentration of resin was increased over three steps. The last infiltration step of 100% resin was carried out overnight. The following day, fully infiltrated samples were cured at 60 °C and left overnight. Semithin sections of 0.5-µm thickness were obtained using a Leica UC6 ultramicrotome (Leica Microsystems GmbH, Wetzlar, Germany) and a Histo-Jumbo diamond knife (Diatome AG, Biel, Switzerland). Sections were stained with 0.1% toluidine blue for 45 s at 65 °C. Section series were documented with a Nikon NiU compound microscope equipped with a DsRi2 camera. Images of complete section series were converted into grayscale using Photoshop (Adobe Inc., San Jose, CA, USA), and used to create 3D-reconstructions in Amira version 2020.2 (FEI, Oregon, USA). Within Amira, the image stacks were aligned with the AlignSlices tool first, before segmenting regions of interest with the segmentation editor. Segmented regions were rendered as surfaces, while parts of the body wall and the opercula were visualized using volume rendering.

### Measurement of zooid dimensions

Zooid dimensions were measured using the NIS Elements software (Nikon, Tokyo, Japan) and Photoshop (Adobe Inc., San Jose, CA, USA). Zooid length was measured along the longitudinal axis from the frontal side of the operculum to the most basal tip of the zooid. The width was measured at the center of the longitudinal axis the operculum width along the basal side of the operculum. The length of the brood chamber of a gonozooid was measured from the frontal to the basal tip along the longitudinal axis.

### Resin casts

For the casting of internal cavities, air-dried shell fragments were cleaned in diluted chlorine for 12 h to remove organic remains and then washed with distilled water—before ultrasonication for 30 s. Afterwards, shell fragments were cleaned with distilled water and dried for 48 h at 60 °C. Once the shells were at room temperature, the Smooth-Cast™ 321 (Smooth-On, Inc., Macungie, Pennsylvania, USA) ultra-low viscosity casting resin was applied to the shell fragments. Best results were achieved when samples were placed in a pressure chamber during curing at 0.4 bar for 40 min. To complete curing, samples were then incubated at 60 °C for 6 h. Once the samples re-adjusted to room temperature, 0.015 M hydrochloric acid (HCL) was applied to dissolve the calcareous shell fragments. HCL was exchanged every 2 h if necessary. Once the shell fragments were completely dissolved, casts were rinsed in distilled water and air dried for further investigation.

### Immunocytochemistry and confocal laser scanning microscopy

Samples fixed in 4% paraformaldehyde (1–2-h room temperature) were used for fluorescence staining. To increase tissue permeability, samples were treated with 2% Triton X-100 and 2% dimethyl sulfoxide (DMSO) diluted in phosphate buffer (PBT) overnight at room temperature. Primary mouse antibodies were applied against acetylated alpha-tubulin (Sigma Aldrich, St. Louis, MO, USA) in a concentration of 1:800. Incubation was carried out overnight at room temperature.

Secondary AlexaFluor 568 goat antibodies against mouse (Invitrogen, Carlsbad, CA) were applied in a concentration of 1:300 together with AlexaFluor 488 phalloidin (Invitrogen) for f-actin labeling in a concentration of 1:100 and DAPI in a concentration of 1:100 for cell nuclei staining. Incubation was carried out overnight and at room temperature. Samples were mounted in Flouromount G (Southern Biotech, Birmingham, LA, USA) on object slides and stored at 4 °C until further investigation. Confocal scans were obtained using a Leica SP5II confocal laser scanning microscope (Leica Microsystems, Wetzlar, Germany). Visualization, reconstruction, and editing of confocal stacks were done in Amira version 2020.2 and Fiji version 1.51 (Schindelin et al., 2012). Images were generated using maximum intensity projection or volume renderings.

### Scanning electron microscopy

Colonies fixed in 2.5% glutaldehyde were first dehydrated in a series of gradually increasing acetone concentrations (from 70 to 100%) followed by critical point drying (CPD) with a Leica EM CPD300 critical point dryer. Samples were either gold sputtered for 120 s using a JEOL JFC-2300HR sputter coater (JEOL, Akishima, Tokyo, Japan) or left blank for EDX analyses. Investigations of samples were carried out using the JEOL IT 300 (JEOL, Akishima, Tokyo, Japan) scanning electron microscope using either a secondary or backscattered electron detector at 10–25 keV.

## Results

### Biogeography and distribution

**Fig. 1:**
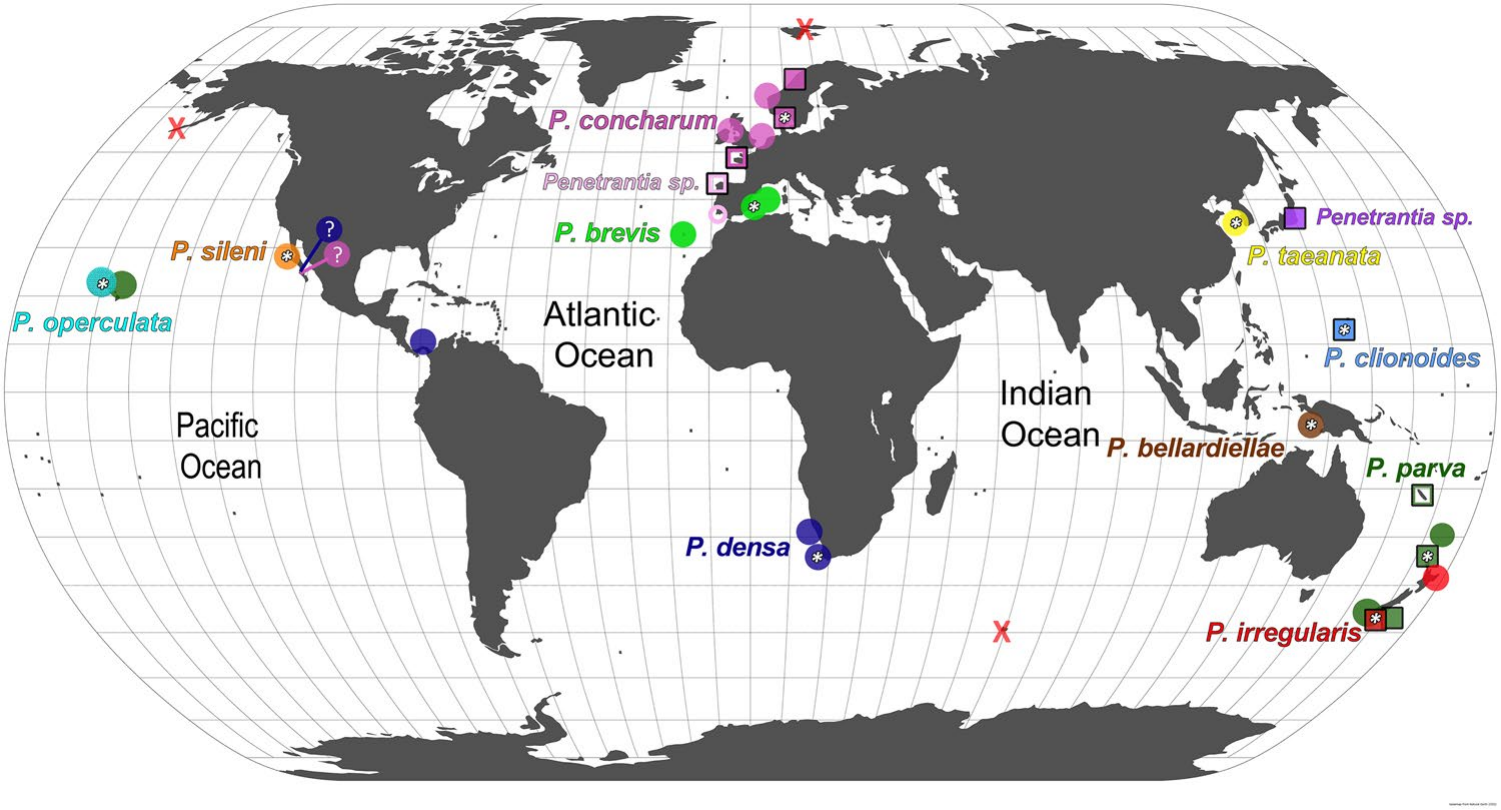
Biogeography of the family Penetrantiidae including all reports of recent species. Map was generated using Khartis 2.1.0 (Sciences Po, Paris, France). Each species is represented in a different color and their type localities are marked with an asterisk. Localities of samples used in this study are indicated as a box and black stroke. (1) Dark blue: *Penetrantia densa* with type locality Cape of Good Hope, South Africa. (2) Bright green: *P. brevis* with type locality Ibiza, Spain. (3) Pink: *P. concharum* with type locality Gullmar Fiord, Sweden. Pink square: *P.* cf. *concharum* from Roscoff, France. (4) Bright pink: *Penetrantia* sp. from the Iberian coast. (5) Dark green: *P. parva* with type locality Hauraki Gulf, New Zealand. Dark green square: *P.* cf. *parva* from New Caledonia. (6) Orange: *P. sileni* with type locality San Benito Islands, Mexico. (7) Red: *P. irregularis* with type locality Otago Peninsula, New Zealand. (8) Cyan: *P. operculata* with type locality Kauai, Hawaii. (9) Bright blue: *P. clionoides* from Guam. (10) Yellow: *P. taeanata* from Taean Coast National Park, South Korea. (11) Brown: *P. bellardiellae* from Papua New Guinea. (12) Purple*: Penetrantia* sp. from Sagami Bay, Kanag, Japan. Red X: no penetrantiids were observed (Aleuten Islands, Svalbard and Kerguelen Islands)

The family Penetrantiidae is distributed globally but restricted to tropical, sub-tropical and temperate seas. No boring traces were found in any of the investigated shells from the Arctic nor the Southern Ocean (Fig. 1).

Most penetrantiid species exhibit strong allopatric distributions with strict regional limits. The only two species with confirmed sympatry are *Penetrantia parva* Silén, 1946 and *Penetrantia irregularis* Silén, 1956 in southern New Zealand (Fig. 1 dark green, red; and Table 2). Nevertheless, *P. parva* and *Penetrantia operculata* Soule & Soule, 1969 were reported from different Hawaiian Islands, which suggests a co-occurrence of these two species in this region (Fig. 1 dark green, cyan; and Table 2). The *Penetrantia concharum* Silén, 1946 species complex in the North Sea of Europe probably includes the sympatric distribution of two or more cryptic species which still needs to be clarified (Fig. 1 pink, bright pink circle; Table 2).

**Table 2:**
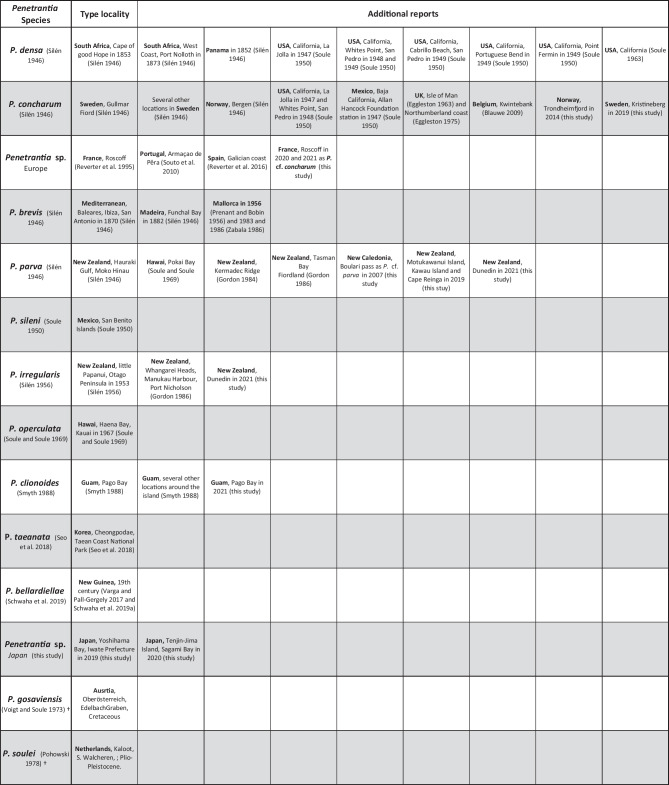
Type locality and all additional reports of each recent penetrantiid species (Eggleston, 1963, 1975; Gordon, 1984; Prenant & Bobin, 1956; Souto et al., 2010; Varga & Pall-Gergely, 2017; Zabala, 1986)

### Biology and substrate diversity

Members of the family Penetrantiidae were predominantly found in shells of molluscs from the intertidal zone to the subtidal zone as deep as 400 m (e.g., *Penetrantia concharum*). In the intertidal, they most commonly occur in living gastropod shells, whereas they frequently colonize dead bivalve shells in the subtidal zone. Overall, extant penetrantiid species or their traces were found in calcareous shells or exoskeletons of 108 different taxa, 105 molluscan species, two cirriped crustaceans and one serpulid polychaete. With 68 different species, gastropods account for the majority of substrates followed by 28 bivalve species and one scaphopod species. Only *P. concharum* from Belgium was reported from non-molluscan shells, the cirripede crustaceans *Balanus balanus* and *Semibalanus balanoides* (De Blauwe, 2009). In Norway *P. concharum* was found in the calcareous tubes of a serpulid polychaete. With 40 different intertidal gastropod species, *Penetrantia clionoides* Smyth, 1988 has the highest range of reported substrates, followed by *P. concharum* with 19 different bored taxa including three different phyla (Mollusca (Gastropoda, Bivalvia), Arthropoda (Crustacea), and Annelida (Polychaeta) (see Online resource S1)).

### Borehole apertures

**Fig. 2:**
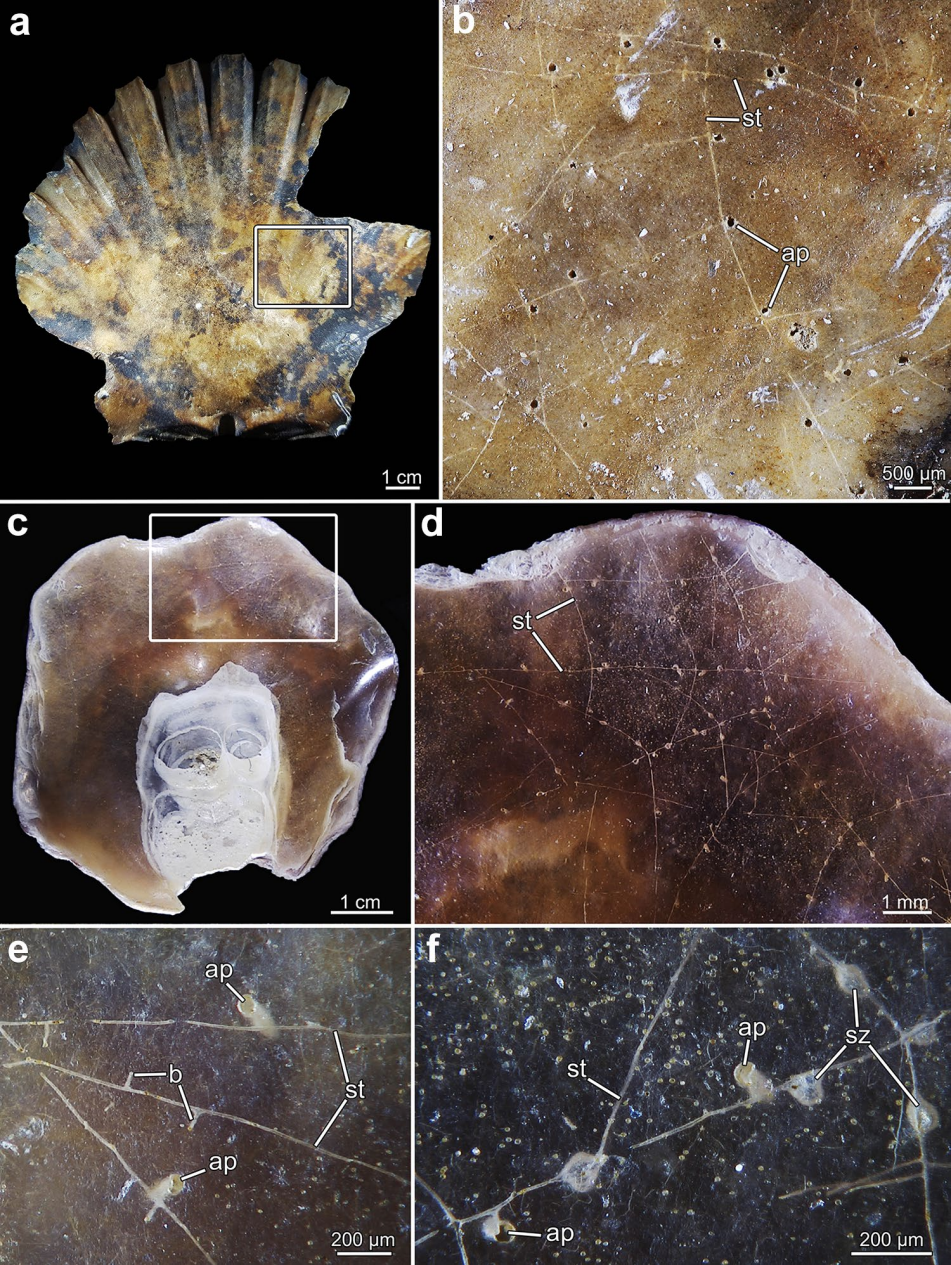
Stereomicroscopic overview of colonies of *Penetrantia concharum* from Sweden in *Pecten maximus* (Linnaeus, 1758) shell **a**, **b**. **b** Close-up of inner shell surface. **c**–**f** *P.* cf. *concharum* from Roscoff, France in *Anomia ephippium* Linnaeus (1758). **d**–**f** Close-up of inner shell surface. **e**, **f** Close-ups of colony shown in **d** with young developing buds in **e** and unique sac zooids in **f**. Squares mark the area of magnification. ap aperture, b bud, st stolon, sz sac zooid

**Fig. 3:**
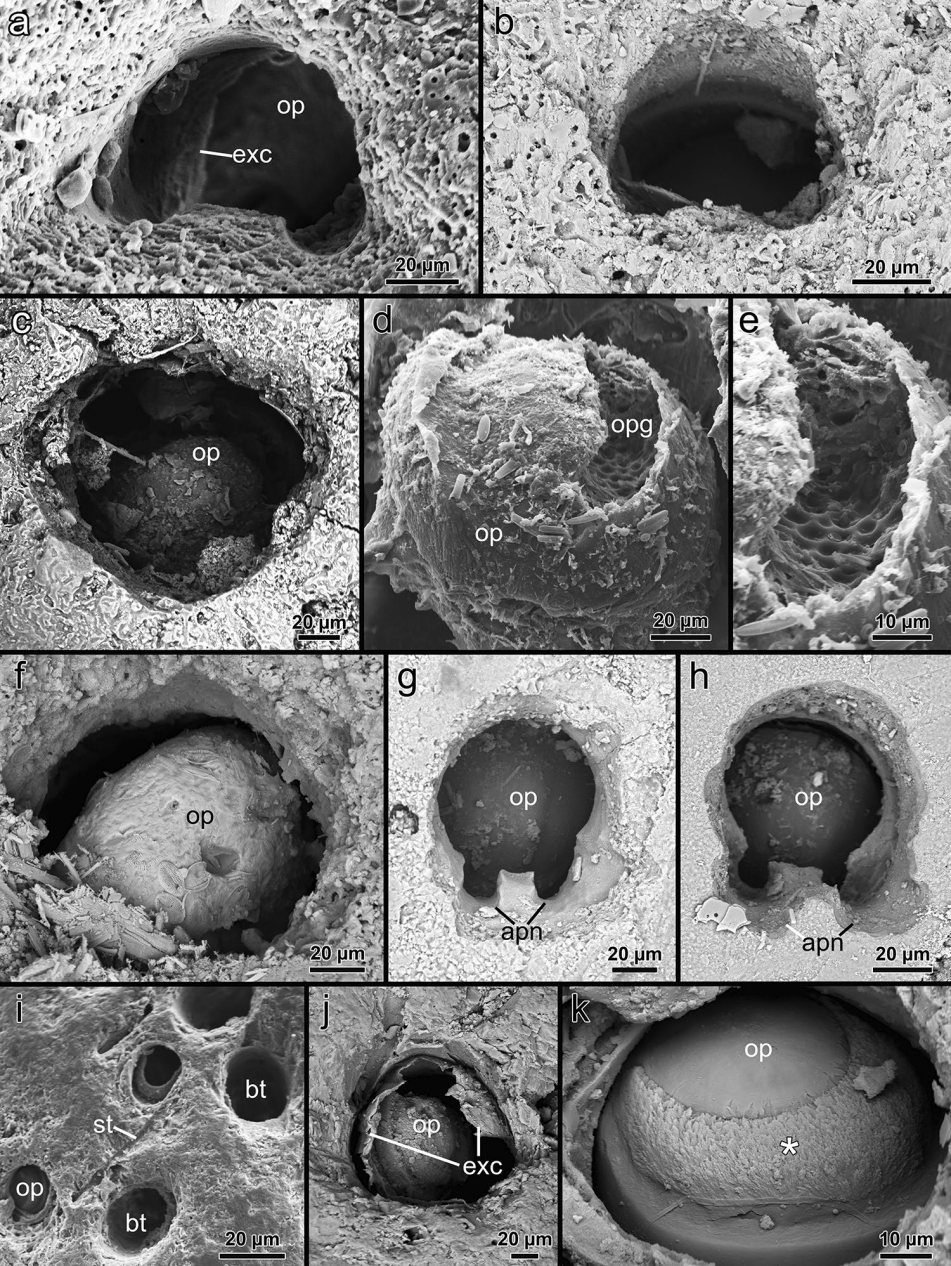
Scanning electron microscopic images of the apertures and opercula of five different penetrantiids. **a** *Penetrantia concharum* from Sweden. **b** *P.* cf. *concharum* from northern France. **c**–**e** *P. irregularis* from southern New Zealand. **f** *P. parva* from northern New Zealand. **g** *P. parva* from southern New Zealand. **h** *P.* cf. *parva* from New Caledonia. **i**–**k** *Penetrantia* sp. from Japan. Asterisk: rough surface area made of calcium carbonate chips. apn apertural notches, bt boring trace, exc exterior cuticle, op operculum, opg opercular groove, st stolon

**Fig. 4:**
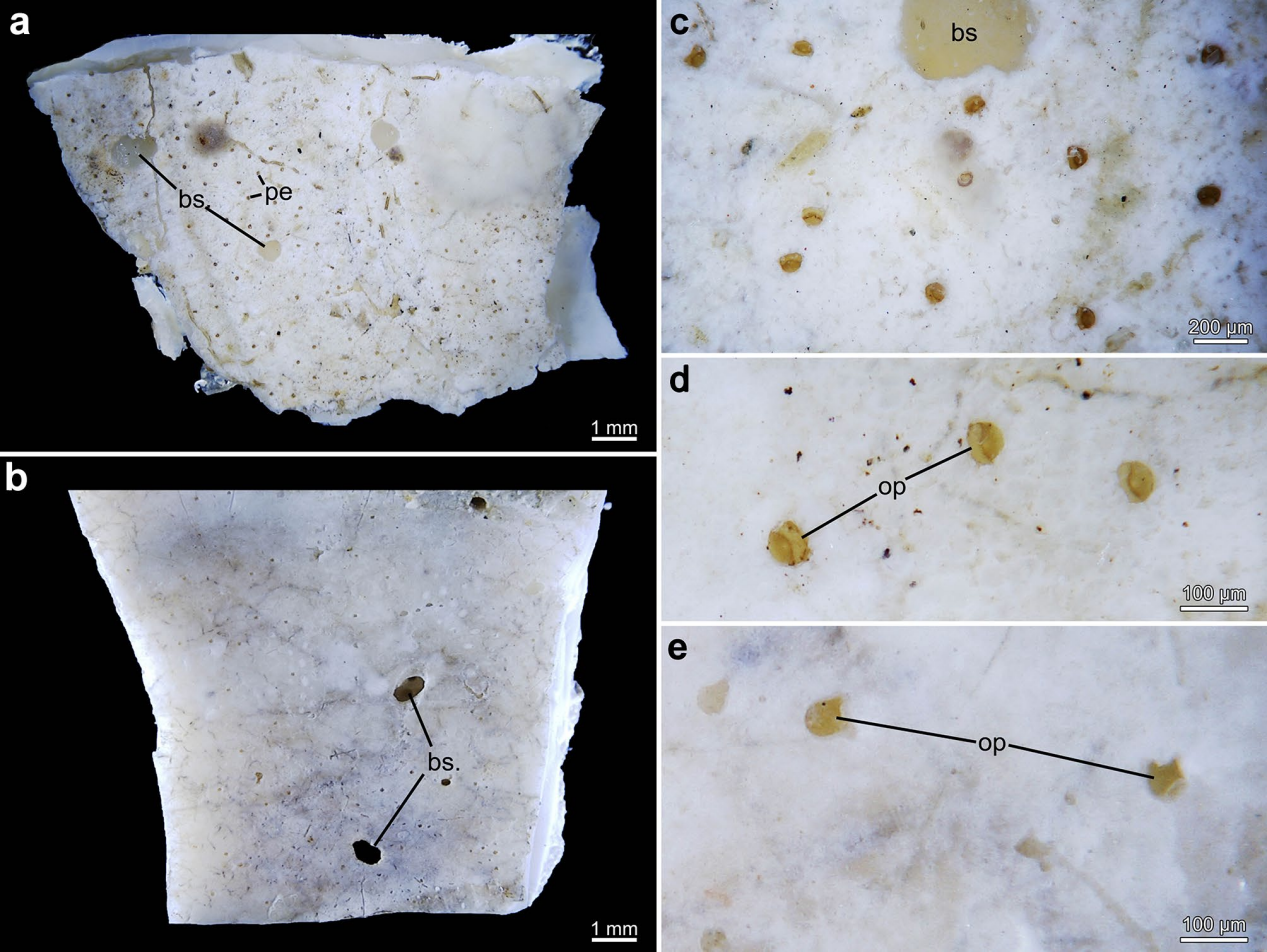
Stereomicroscopic overview of colonies and apertures of *Penetrantia parva* and *P. irregularis* from Southern New Zealand within the same fragment of undetermined bivalve shell. **a** Overview of outer shell surface with *P. irregularis* and sponge borings. **b** Overview of inner shell surface with *P. parva* borings. **c**, **d** Close-ups of *P. irregularis* apertures. **e** Close-up of *P. parva* apertures. bs bioeroding sponge, op operculum, pe *Penetrantia*

**Fig. 5:**
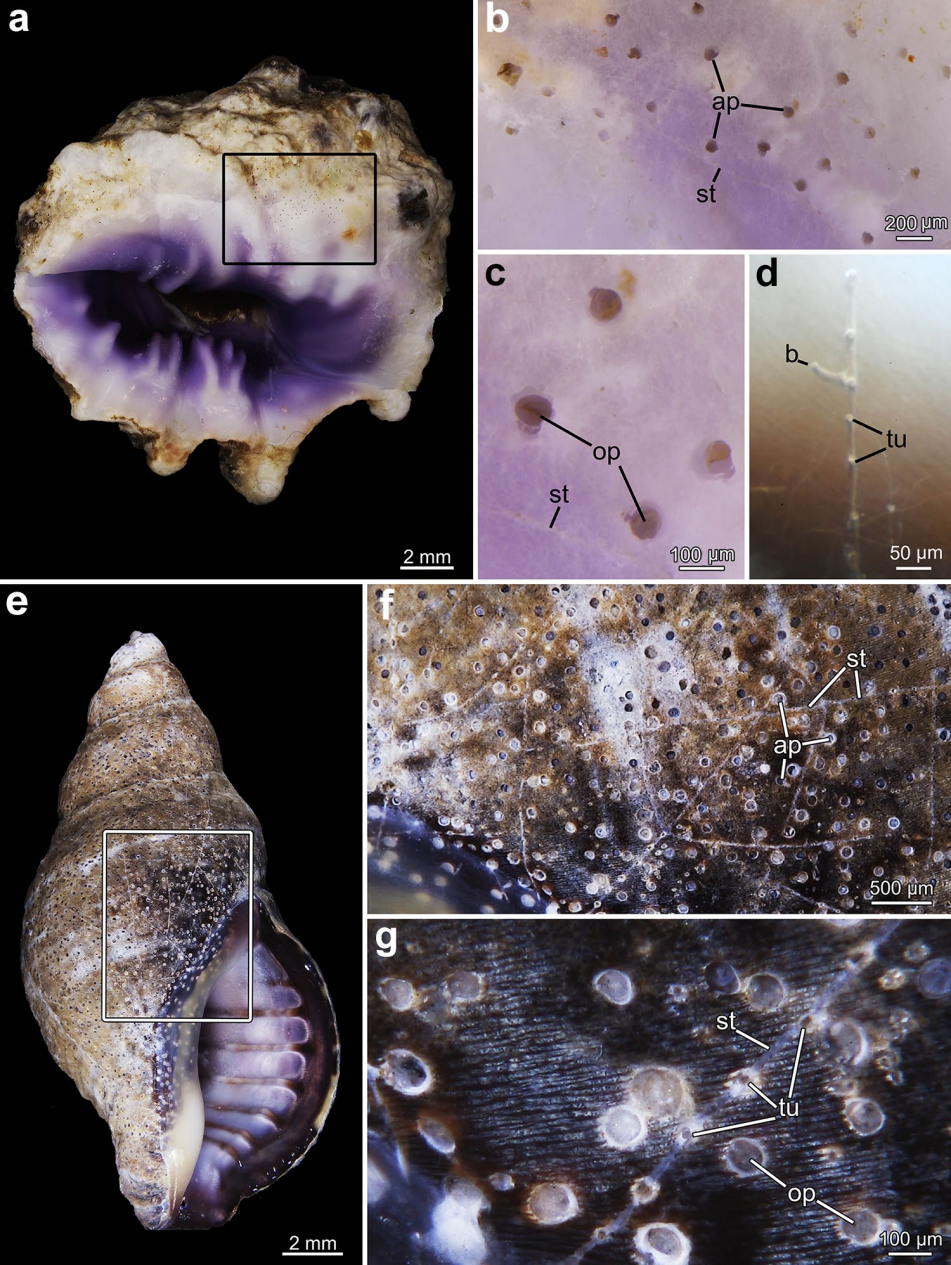
Stereomicroscopic overview of colonies and apertures. **a**–**d** *Penetrantia clionoides* from Guam in *Drupa morum* Röding, 1798. **e**–**g** *Penetrantia* sp. from Japan in *Japeuthria ferrea* (Reeve, 1847). **a** Overview of *Drupa morum* shell. Square marks area of magnification. **b** Detailed overview of borings close to aperture of shell. **c** Close-ups of apertures with opercula. **d** Stolon with tubulets and developing bud. **e** Overview of live *Japeuthria ferrea* shell. Square marks area of magnification. **f** Detailed overview of borings close to aperture of shell. **g** Close-ups of apertures and tubulets. ap aperture, b bud, op operculum, st stolon, tu tubulet

Each zooid creates an opening on the surface of the substrate though which it protrudes its lophophore into the surrounding water. Hereafter, the term aperture refers to the outer margin of the entire borehole. These apertures are the most obvious indication of the presence of a colony and are usually 50–100 µm in width. The shape of the apertures can vary between the different species and is somewhat kidney-shaped in most species particularly in *Penetrantia concharum* and* P*. cf. *concharum* from France (Figs. 2 and 3a, b). The side of the free margin of the operculum is rounded, whereas the opposite side where the operculum is hinged to the zooid is either straight, slightly dented or shows hinge-like notches (Fig. 3). The convex side of the aperture, at the free margin of the operculum, is always directed towards the growth direction of the corresponding stolon. Apertural notches are very prominent in *P. parva* and *P.* cf. *parva* from New Caledonia (Figs. 3f–h and 4e). The apertures of *P. irregularis* are almost circular in shape but are more pointed on the oral side compared to the anal side. No apertural notches were observed and with a mean diameter of 126 µm, these openings were the widest in this study (Figs. 3c and 4c, d). The apertures of *P. clionoides* and *Penetrantia* sp. from Japan are very similar in size and shape and are circular to elliptical with some being unique key-hole shaped with a sinus-like cutout on the oral side (Figs. 3i and 5).

### Colony and stolon morphology

**Fig. 6:**
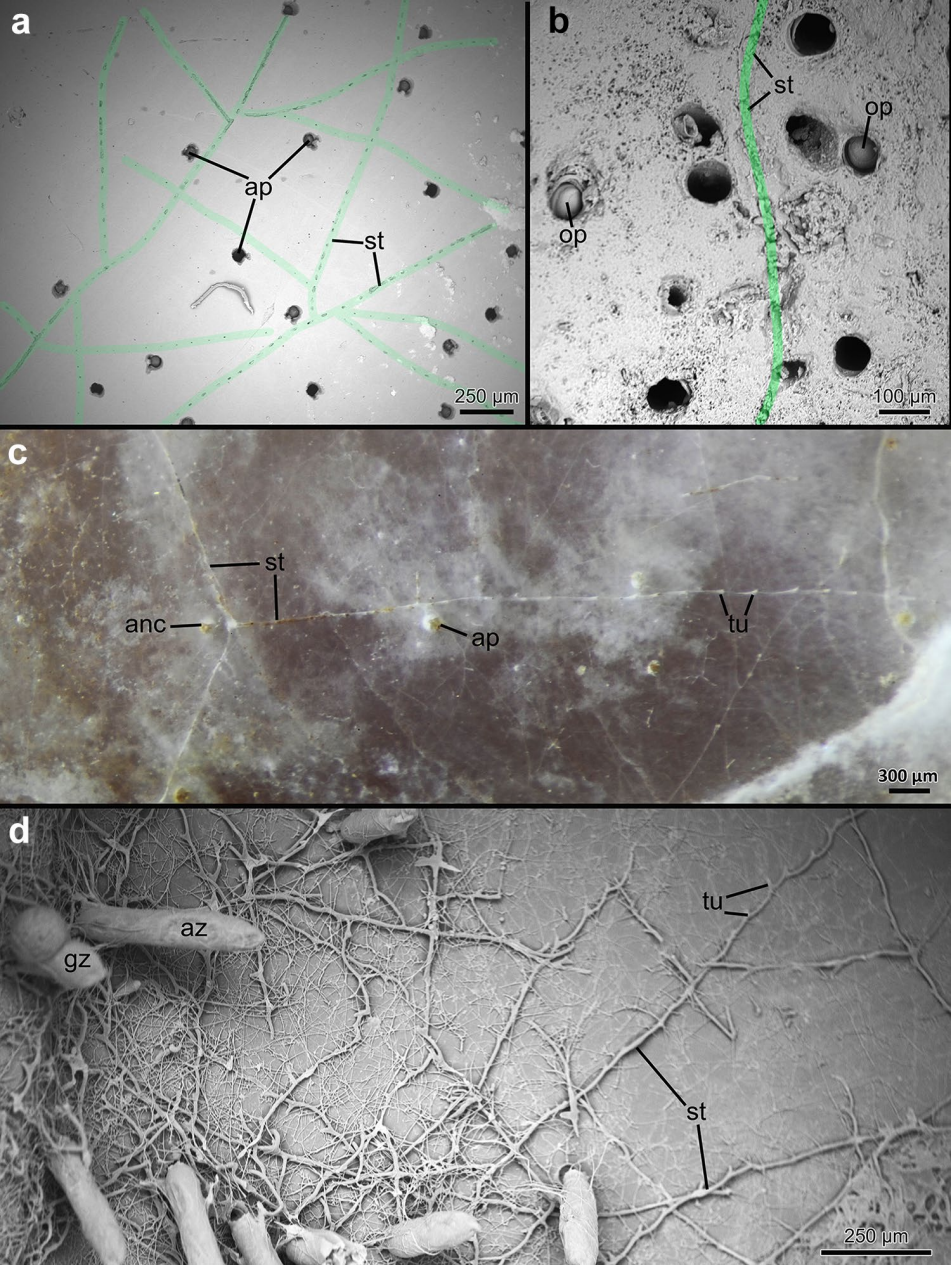
Overview of the stolonal network in the family Penetrantiidae. **a** Scanning electron microscopic image of *Penetrantia* cf. *parva* from New Caledonia. Stolonal network marked in green. **b** Scanning electron microscopic image of *Penetrantia* sp. from Japan. Stolon marked in green. **c** Stereomicroscopic image of a young colony of *P.* cf. *concharum* from France. **d** Scanning electron microscopic image of a resin cast of *P. parva* from northern New Zealand. ap aperture, az autozooid, anc ancestrula, gz gonozooid, op operculum, st stolon, tu tubulet

**Fig. 7:**
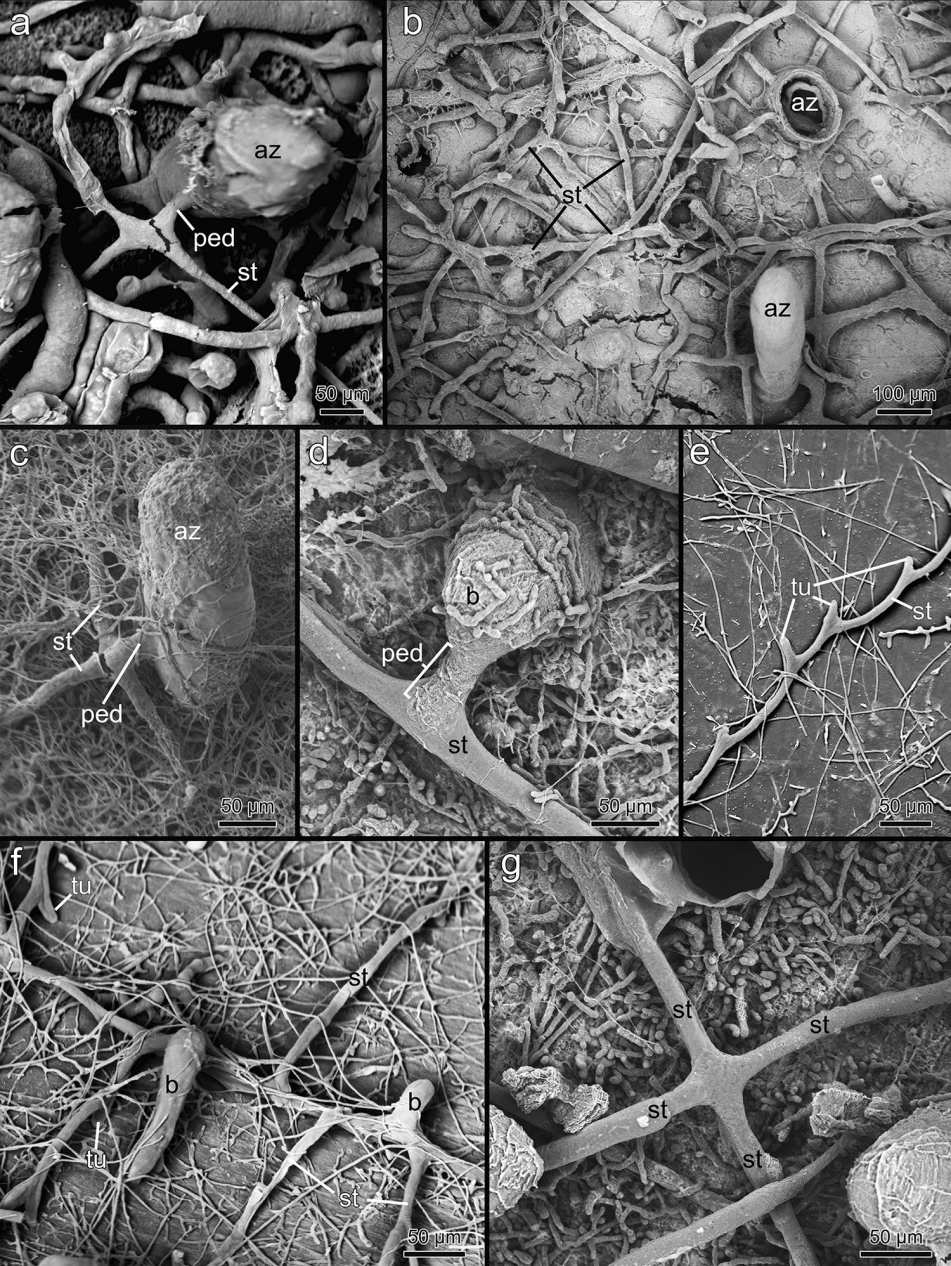
Scanning electron microscopic image of resin casts showing autozooids and their stolonal connection. **a** *Penetrantia concharum* from Sweden. **b** *P. irregularis* from southern New Zealand. **c** *P. parva* from northern New Zealand. **d** Younger bud of *Penetrantia* sp. from Japan. **e** Stolon with tubulets of *P. parva* from northern New Zealand. **f** Branching stolons and two early buds in *P. parva* from northern New Zealand. **g** Branching of the principal stolon in *Penetrantia* sp. from Japan. az autozooid, b bud, ped peduncle, st stolon, tu tubulet

All penetrantiids except for *Penetrantia irregularis* have a very similar basic colonial arrangement with zooids interconnected by a network of kenozooidal stolons. The principal stolon emerges from the ancestrula and parallel to and about 20–100 µm below the surface of the substrate. In *P. concharum* and *P.* cf. *concharum*, three principal stolons radiate from the ancestrula at an angle of 120° to each other creating a star-shaped ancestrula complex (Fig. 6c). The principal stolon creates lateral branches at certain intervals. These branches of the principal stolon emerge orthogonally (Figs. 6a, d and 7f, g). While the lateral branches grow, they usually bend slightly towards the growth direction of the main principal stolon giving the colony its characteristic feather-shaped appearance (Figs. 2d, 5f, g and 6a). However, in larger and older colonies, the stolonal network can be extremely ramified by different stolon branches from different parts of the colony, crossing and interconnecting, giving it a mesh-like appearance (Figs. 2d, 5f and 6d). Autozooids are located along both lateral sides of the stolon in regular intervals and always connect to the stolonal network by short peduncles (Figs. 2e, f, 5 g, 6b and 7a, c, d). In all investigated penetrantiids, autozooids and gonozooids are angled vertically in relation to the surface of the substrate (Figs. 6d and 7a–d). Autozooids of *P. concharum* and *P.* cf. *concharum* from France (Fig. 2b, d) are more widely spaced than in *P. parva* and *Penetrantia* sp. from Japan (Fig. 5f). In older colonies, zooids appear to be more abundant and generally less spaced, as new buds also emerge along older parts of the principal stolons (Fig. 5f, g).

Additional adventitious stolons carry no autozooids and interconnect different parts of the principal stolon, which significantly increases the ramification especially in older colonies. These adventitious stolons branch off randomly and fuse with every stolon they cross and sometimes with autozooids too, which creates secondary non-pedunculate connections to the stolonal network (Figs. 6d and 7b). This mode of growth seems to be the primary condition in colonies of *P. irregularis*, where principal stolons are hardly recognizable. The stolonal network in this species is extremely ramified, and zooids can have several stolonal connections (Fig. 7b).

**Fig. 8:**
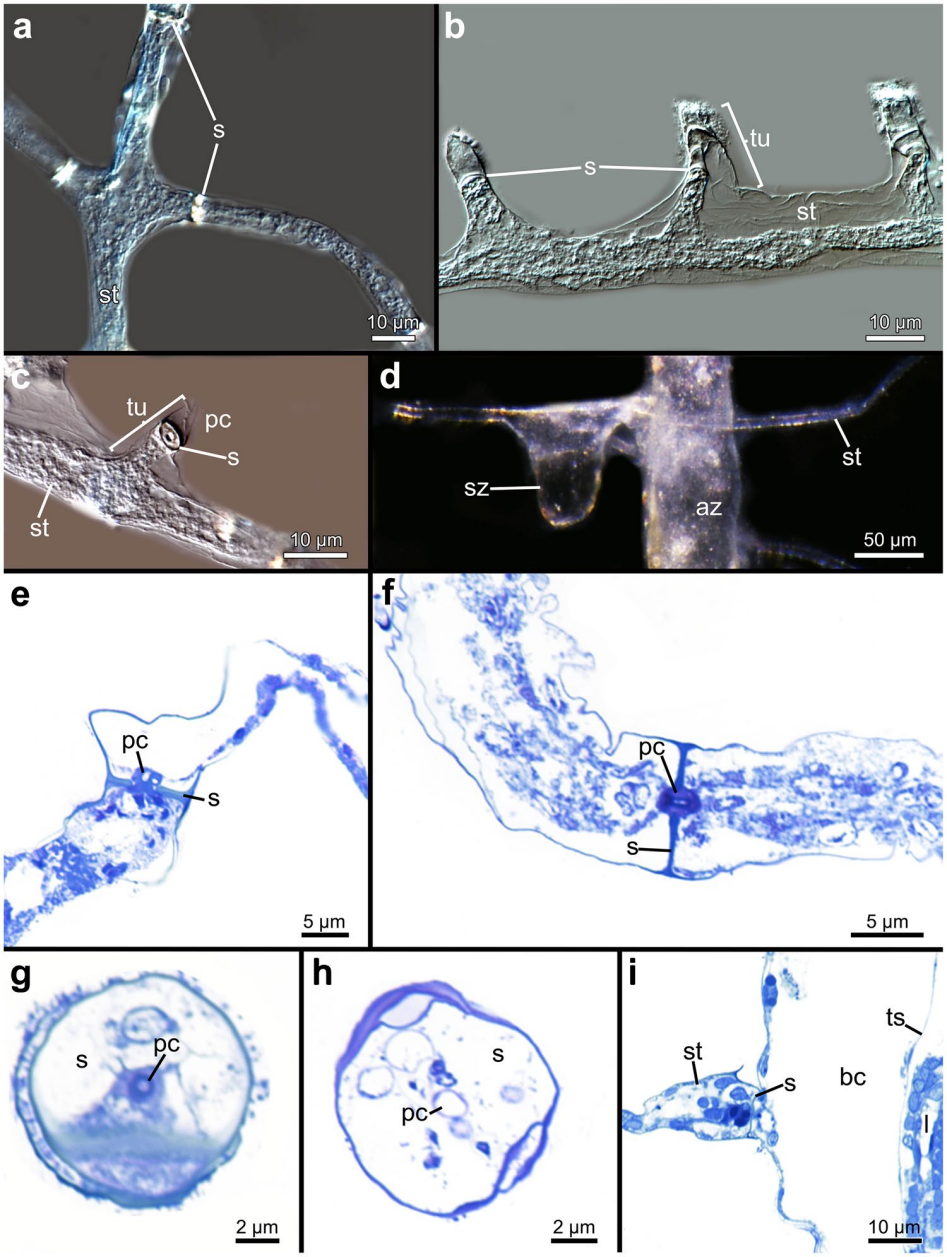
Stolon morphology of the family Penetrantiidae. **a**–**d** Stereomicroscopic images of stolons and tubulets of *Penetrantia* sp. from Japan after decalcification of substrate. **d** Sac zooid in *Penetrantia concharum* from Sweden. **e** Histological semi thin section through the peduncle of *P. clionoides* from Guam. **f** Histological semi thin section through the peduncle of *Penetrantia* sp. from Japan. **g** Histological semi thin cross section through the stolon of *P. concharum* from Sweden. **h** Histological semi thin cross section through the stolon of *Penetrantia* sp. from Japan. **i** Adventitious stolon connecting to an autozooid in *P. concharum* from Sweden. az autozooid, bc body cavity, pc pore complex, s septum, st stolon, sz sac zooid, ts tentacle sheath, tu tubulet

Individual stolonal kenozooids that form the stolonal network are separated by interzooidal septa (or pore-plates), each with a single pore in all investigated penetrantiids (Fig. 8). The pore is 1–2 µm in diameter and is associated with one special cell that plugs the pore (Fig. 8c, e–i). The cystid wall of the stolonal tubes are delicate and thin in all species. Stolons are approximately circular in cross section and vary from 5 to 15 µm in diameter (Fig. 8g, h). Septa can also be present at the junction of the stolonal network, e.g., where the principal stolon branches or where adventitious stolons branch off the principal stolon (Fig. 8a).

**Fig. 9:**
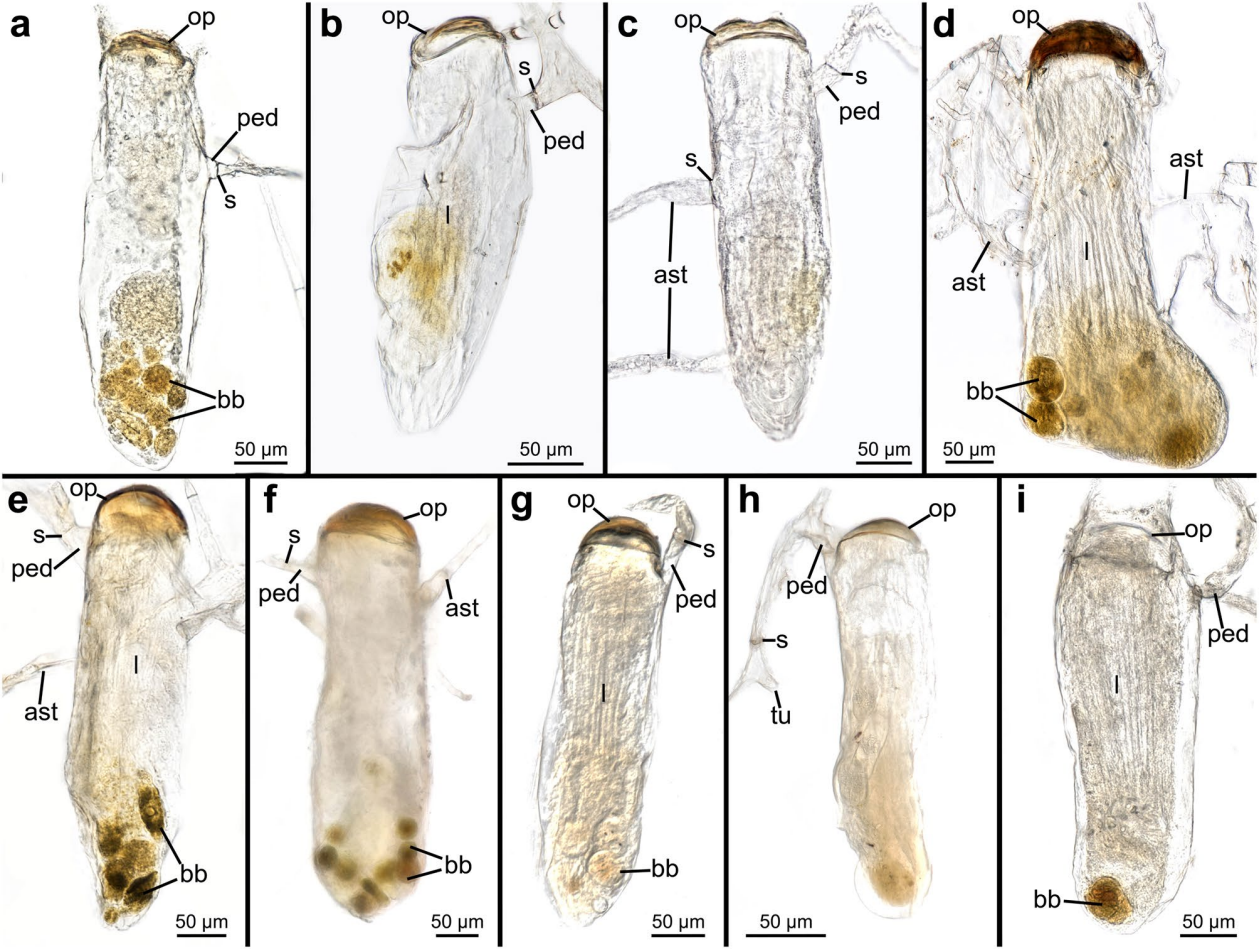
Stereomicroscopic images of whole mounts of autozooids of all here investigated penetrantiids. **a** *Penetrantia concharum* from Sweden. **b** *P.* cf. *concharum* from France. **c** *P. concharum* from Norway. **d** *P. irregularis* from southern New Zealand. **e** *P. parva* from northern New Zealand. **f** *P. parva* from southern New Zealand. **g** *P.* cf. *parva* from New Caledonia. **h** *P. clionoides* from Guam. **i** *Penetrantia* sp. from Japan. ast adventitious stolon, bb brown body, l lophophore, op operculum, ped peduncle, s septum, tu tubulet

The peduncle is part of the autozooid and ends at the septum, which separates it from the stolonal network and attaches orthogonally to the corresponding stolon (Fig. 7a, c, d), with the exception of *P. irregularis* where the angle varies between zooids (Figs. 7b and 9d). When adventitious stolons attach to autozooids and create additional connections to the stolonal network, the septa are located directly at the border between autozooidal body wall and stolon (Figs. 8i and 9c).

In most species, the stolons are not simple straight tubes between zooids, but instead the stolonal tube appears serrated, with short, regular sections called tubulets that curve towards the surface of the substrate where they terminate. These small openings are approximately 5–10 µm in diameter (Figs. 6a, 7e and 8b, c). After each tubulet, the next stolon section starts and sometimes is separated by a septum from the latter. The next section grows in basal direction until half its length before it proceeds as a tubulet towards the surface again. This growth pattern results in a serrated appearance of the stolon branches from a lateral perspective (Figs. 6d, 7e and 8b). This is found in all investigated penetrantiids in this study, but the distance or interval between tubulets varies between species as well as the length of the tubulet itself (Table 3). The largest interval between tubulets ranges from 184 to 300 µm in *P. concharum*, while the distance is significantly shorter in *P. parva*, 73–132 µm. There is also a large difference between *P. clionoides* and *Penetrantia* sp. from Japan (Fig. 5d, g). The latter has a mean interval of 206 µm whereas only 96 µm is present in *P. clionoides* (Table 3). In *Penetrantia* sp. from Japan, a uniporous septum is present just before the end of each tubulet, while the cuticle extends further towards the surface (Fig. 8c).

Sometimes so-called sac-zooids (see Pohowsky, 1978) are attached to the stolons as basal bag-like extensions. These sac-zooids are part of the corresponding stolon and are not separated by septa. They range from about 50–100 µm in basal direction. In *P. parva* from Northern New Zealand, sac zooids are packed with granules (Fig. 8d) (see section on "Stolonal musculature").

**Table 3:**
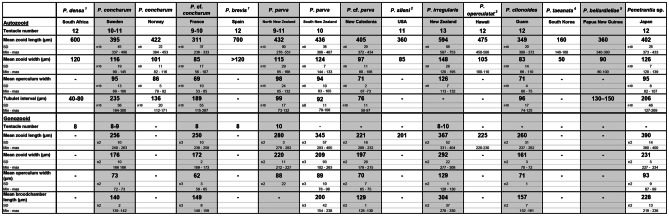
Summary of zooid dimension and tentacle number of all recent members of the family Penetrantiidae

Information about (1) *Penetrantia densa* and *P. brevis* according to Silén (1946). (2) *P. sileni* according to Soule (1950). (3) *P. operculata* according to Soule and Soule (1969b). (4) *P. taeanata* based on Seo et al. (2018). (5) *P. bellardiellae* according to Schwaha et al. (2019a)

### Autozooid morphology

The following characters relate to retracted zooids only.

### Gross morphology

**Fig. 10:**
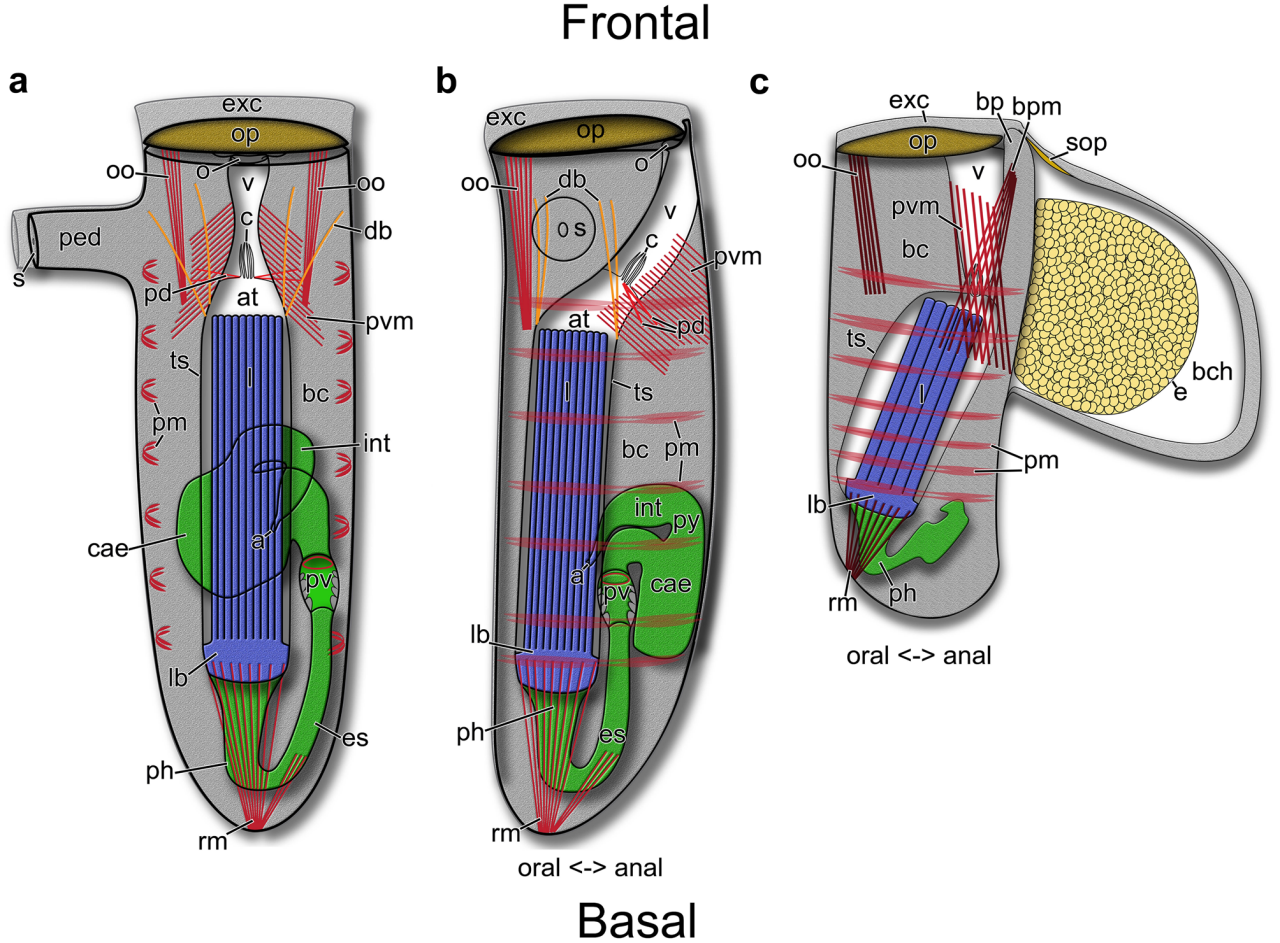
Schematic drawing based on *Penetrantia concharum* from Sweden. **a** Autozooid in longitudinal section from an oral view. **b** Autozooid in longitudinal section from a lateral view with the anal side on the right. **c** Gonozooid in longitudinal section from a lateral view with the anal side on the right. Blue: lophophore, green: digestive system, red: musculature, orange: duplicature bands. a anus, at atrium, bc body cavity, bch brood chamber, bp brood chamber plug, bpm brood chamber plug muscle, c collar, cae caecum, db duplicature band, e embryo, es esophagus, exc exterior cuticle, int intestine, l lophophore, lb lophophoral base, o orifice, oo operculum occlusor, op operculum, pd parieto-diaphragmatic muscle, ped peduncle, ph pharynx, pm parietal muscles, pv proventriculus, pvm parieto-vestibular muscle, py pylorus, rm retractor muscle, s septum, sop secondary operculum, ts tentacle sheath

**Fig. 11:**
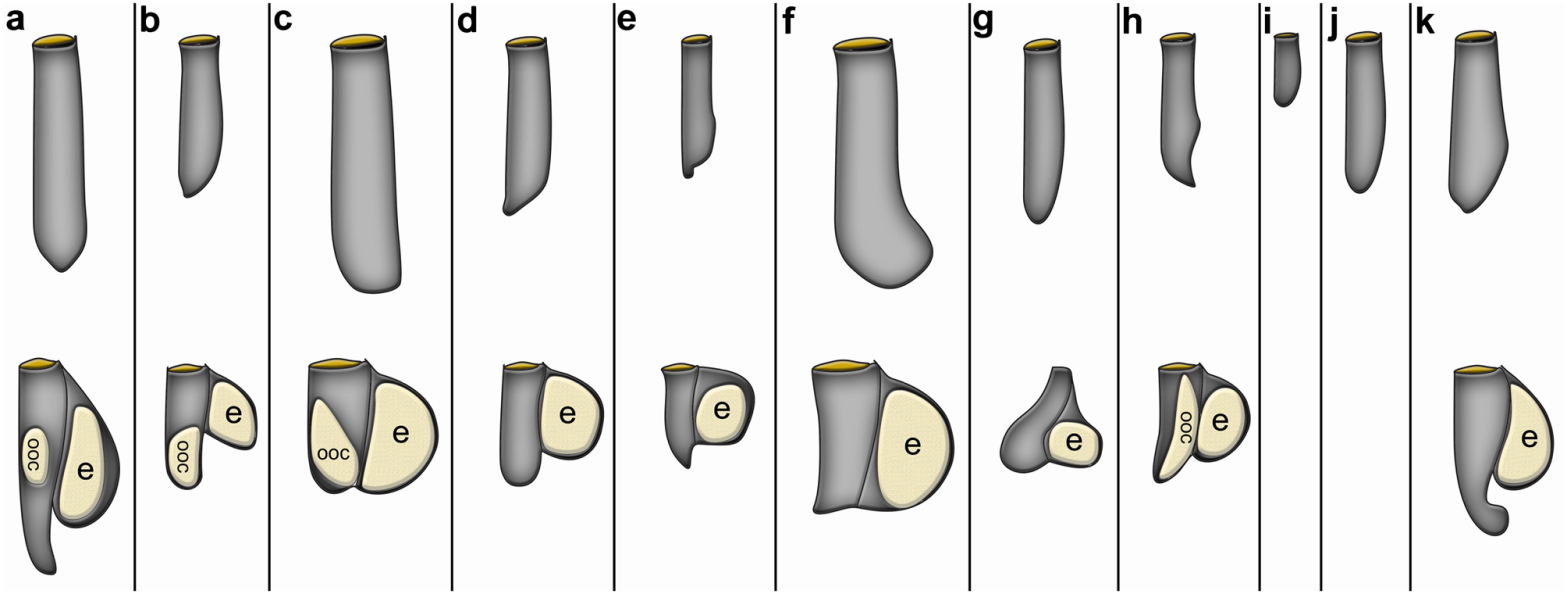
Schematic drawing of all members of the family Penetrantiidae. In the upper row are the autozooids and below the corresponding gonozooid where information was available. **a** *Penetrantia densa* according to Silén (1946). **b** *P. concharum* from Sweden. **c** *P. brevis* according to Silén (1946). **d** *P. parva* from northern New Zealand. **e** *P. sileni* according to Soule (1950). **f** *P. irregularis* from southern New Zealand. **g** *P. operculata* from Hawaii according to Soule and Soule (1969b). **h** *P. clionoides* from Guam. **i** *P. taeanata* from South Korea according to Seo et al. (2018). **j** *P. bellardiellae* from Papua New Guinea according to Schwaha et al. (2019a). **k** *Penetrantia* sp. from Japan. e embryo, ooc oocyte

Autozooids are arranged vertically in the substrate and are shaped like narrow elongated tubes approximately five times longer than wide (Fig. 9). The frontal side is orientated towards the surface where the operculum and orifice are situated. The basal side is positioned deep below the surface. (Figs. 9 and 10). Autozooids vary in size significantly across species, from 140 up to 700 µm in length and from 50 to 148 µm in width (Table 3; Fig. 11). *Penetrantia taeanata* is by far the smallest member, whereas the largest autozooids are found in *P. brevis*, *P. densa*, and *P. irregularis* with 700 µm, 600 µm, and almost 600 µm in mean length, respectively. Most penetrantiids have medium-sized autozooids with a length of 300–450 µm and width of 83–120 µm: *P. concharum*, *P. parva*, *P. sileni*, *P. operculata*, *P. clionoides*, *P. bellardiellae*, and *Penetrantia* sp. from Japan. The highest variation in zooid length was observed in *P. irregularis* with zooids ranging from 507 to 753 µm. A notable difference in size is present between the Swedish *P. concharum* and *P.* cf. *concharum* from France. Autozooids of *P. concharum* are approximately one-third longer and wider on average than autozooids of *P.* cf. *concharum* from France, the same difference as for the operculum width. The Norwegian *P. concharum* shows similar zooid dimensions as *P. concharum* from Sweden. Specimens of *P. parva* from the North and South Islands of New Zealand are very similar in size. Smaller differences are present between the two *P. parva* from New Zealand and *P*. cf. *parva* from New Caledonia. The latter has a mean autozooid length 30 µm shorter, and the autozooids are significantly narrower by about a quarter (Table 3).

The autozooidal tube is either straight or slightly curved towards the anal side as in *P. concharum* and *P. parva* (Fig. 11b, d)*.* In many species, the basal end narrows and is pointed as in *P. concharum*, *P. parva*, and *P. sileni* (Fig. 11b, d, e), whereas the basal end is blunter and sock-shaped in *P. densa*, *P. brevis*, and even enlarged in *P. irregularis* (Fig. 11a, c, f). Interestingly, such a blunt basal end was only found in penetrantiids with a mean zooid length of more than 590 µm, while species with shorter zooids tend to have a pointed basal tip (Table 3).

### Operculum

**Fig. 12:**
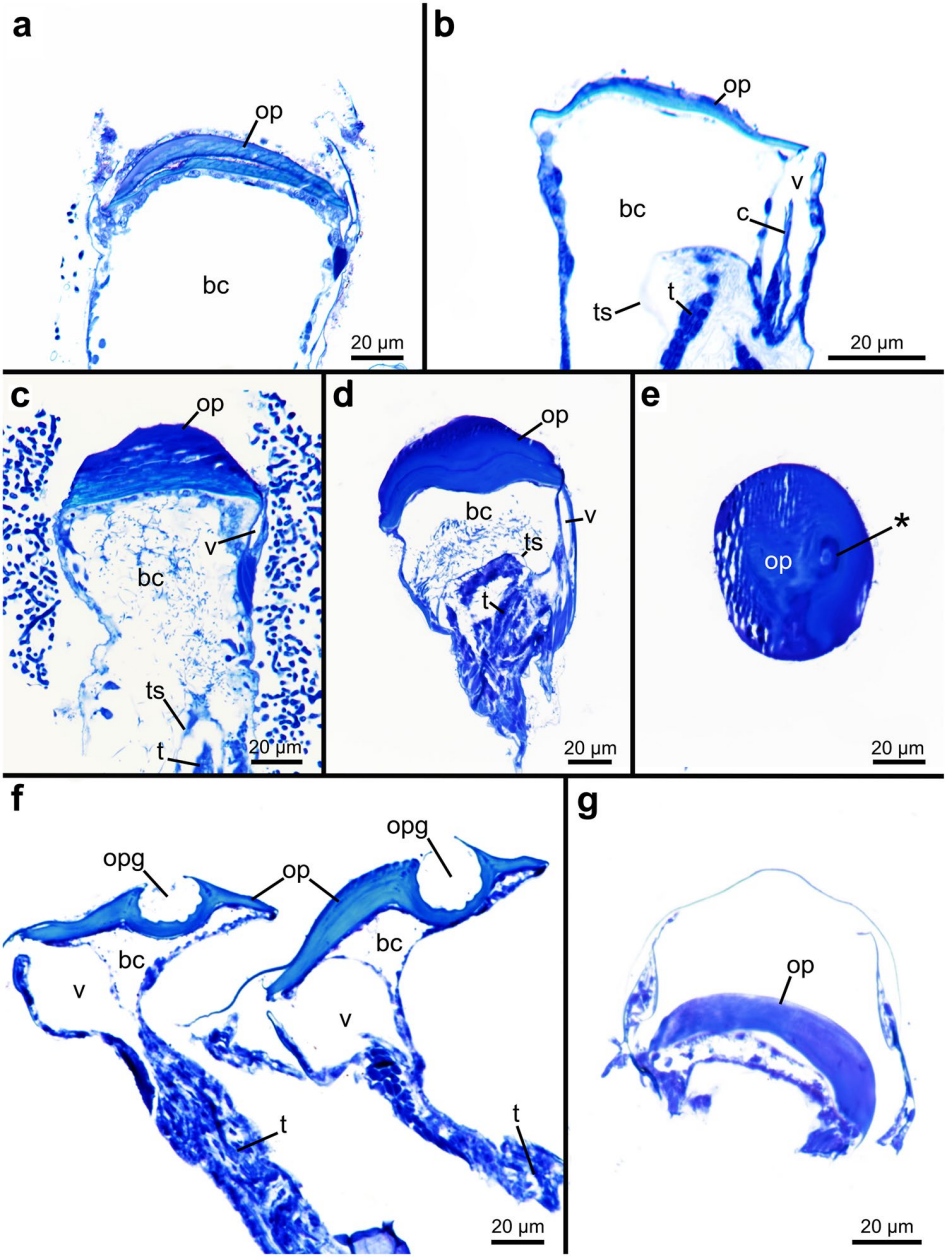
Histological semi thin sections of the opercula of five different penetrantiids. **a** *Penetrantia concharum* from Sweden. **b** *P.*cf. *concharum* from France. **c** *P. parva* from northern New Zealand. **d**–**e** *P. parva* from southern New Zealand. Asterisk marks the opercular pit on the frontal side. **f** *P. irregularis* from southern New Zealand. **g** *P. clionoides* from Guam. bc body cavity, c collar, op operculum, opg opercular groove, t tentacle, ts tentacle sheath, v vestibulum

All penetrantiids have a characteristic operculum that seals the frontal side of the zooid and effectively closes the orifice. Opercula are circular to elliptical and lie slightly below the aperture in most species (Fig. 3). The diameter can vary significantly among species. from 50 to 100 µm (Table 3). In cross section, the opercula are cap-shaped in most species with rather flat edges and increasing height towards the center. The opercula are more dome-shaped in *P. parva* from northern New Zealand, *P. parva* from southern New Zealand, and *P.* cf. *parva* from New Caledonia (Fig. 12c, d). They are heavily cuticularized in most species, unlike the regular body wall (Fig. 12). The opercula of *P. concharum* from Sweden and particularly of *P.* cf. *concharum* from France are less cuticularized (Fig. 12a, b). Additionally, the operculum of *P. concharum* from Sweden has a convex frontal margin in cross section, while in *P.* cf. *concharum* from France, the margin of the operculum is flatter (Fig. 12a, b). The frontal surface of the operculum is smooth and plain in most species. Some opercula of *P. parva* from New Zealand can have a shallow pit in the center of the frontal side (Figs. 3f and 12e). The operculum of *P. irregularis* has a very characteristic groove along its frontal-oral side, about 80 µm long and 20 µm wide, which opens towards the frontal side. Internally, the groove is covered by comb-shaped pits (Figs. 3d, e and 12f). A rough, crescent-shaped patch on the frontal side, almost in identical position, is present in opercula of *Penetrantia* sp. from Japan. The remaining part of the operculum is smooth (Fig. 3k). Additional energy-dispersive x-ray (EDX) spectroscopic data shows that the operculum of *Penetrantia* sp. from Japan is partially composed of calcium (Online Resource S2).

### Body wall

**Fig. 13:**
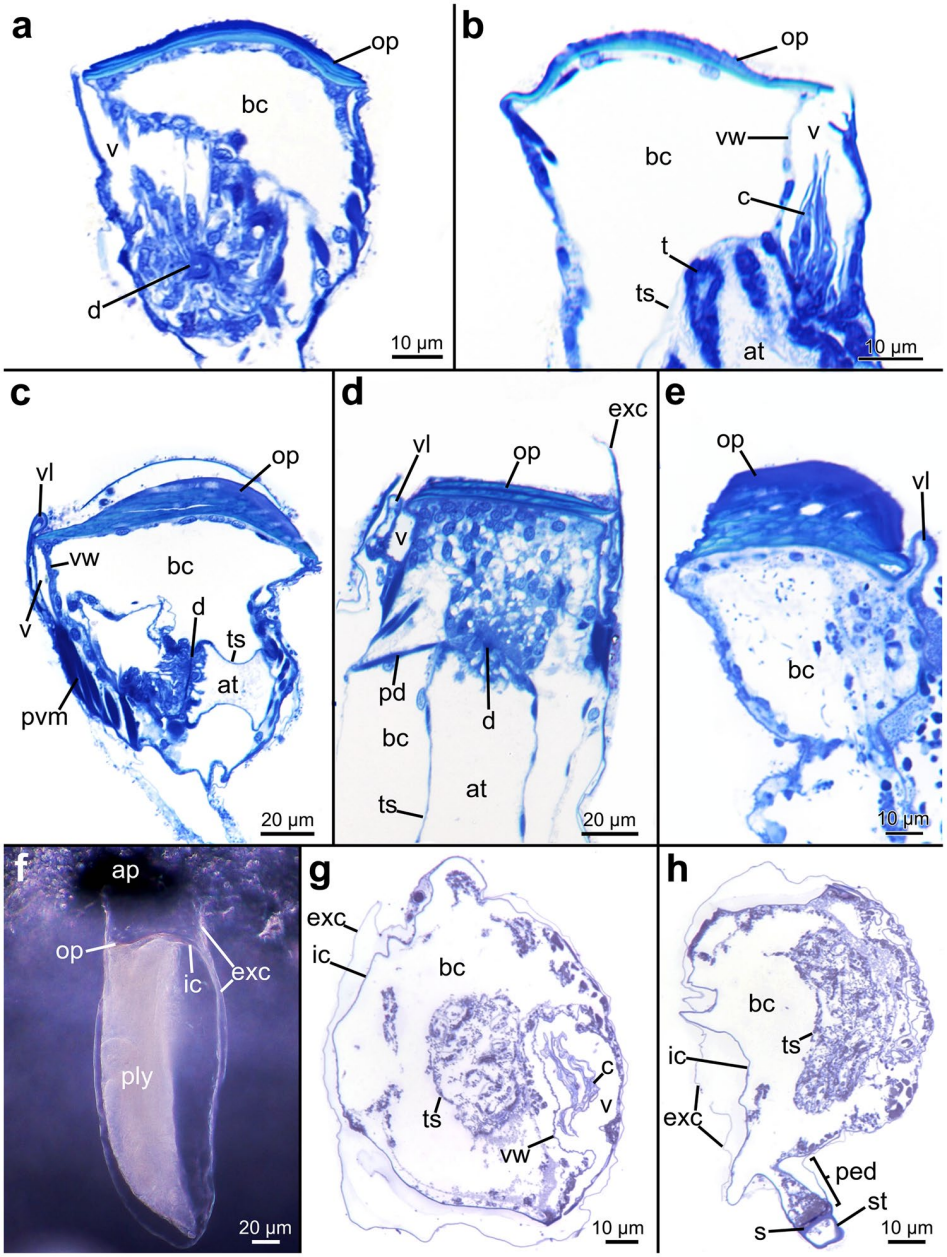
Histological semi thin sections through the orifice and vestibular area of four different penetrantiids. **a**–**b** *Penetrantia* cf. *concharum* from France. **c**–**d** *P. concharum* from Sweden. **e** *P. parva* from Northern New Zealand. **f** Stereomicroscopic image of an autozooid of *Penetrantia* sp. from Japan. **g**–**h** *Penetrantia* sp. from Japan. ap aperture, at atrium, bc body cavity, c collar, d diaphragm, exc exterior cuticle, ic inner cuticle, op operculum, pd parieto-diaphragmatic muscle, ped peduncle, ply polypide, pvm parieto-vestibular muscle, s septum, st stolon, t tentacle, ts tentacle sheath, v vestibulum, vl vestibular lip, vw vestibular wall

The body wall or cystid of all investigated penetrantiids is composed of two cuticles, an exterior and interior one. This structure is most obvious in the frontal area of the autozooids around the orifice. The inner cuticle invaginates into the vestibulum whereas the exterior cuticle runs further in frontal direction to line the most frontal part of the borehole, as in *P. concharum* from Sweden and *Penetrantia* sp. from Japan. In the basal part of the zooids, both cuticles are tightly aligned often rendering their differentiation impossible (Figs. 3j and 13d, f–h). At the peduncle of autozooids, the inner cuticle terminates at the septum but the exterior cuticle proceeds further and becomes the exterior cuticle of the adjoining stolon (Fig. 13h).

### Soft body morphology

#### Orifice

At the orifice, the inner cuticle of the body wall invaginates to become the vestibular wall. In some species, the anal portion of the vestibular wall extends frontally of the orifice to create a clamp-like structure that latches the anal edge of the operculum, a feature particularly obvious in *P. concharum* from Sweden and *P. parva* from northern New Zealand (Fig. 13c–e). The vestibular wall is a uniform, thin epithelium and does not show any signs of vestibular glands. It proceeds basally and terminates at the diaphragm, which separates the vestibular wall from the tentacle sheath (Fig. 10). At the diaphragm, a collar is present in most species investigated here. Partial or complete reductions of the collar are evident in *P. concharum* from Sweden and *P. parva* from northern New Zealand (Figs. 10, 12b, f and 13a–c, g). In contrast, *P.* cf. *concharum* from France, *Penetrantia* sp. from Japan, and *P. clionoides* show a prominent setigerous collar (Figs. 12b and 13b, g). In the last-mentioned, the collar is almost as long as the entire vestibulum (Fig. 13b).

#### Tentacle sheath and lophophore

**Fig. 14:**
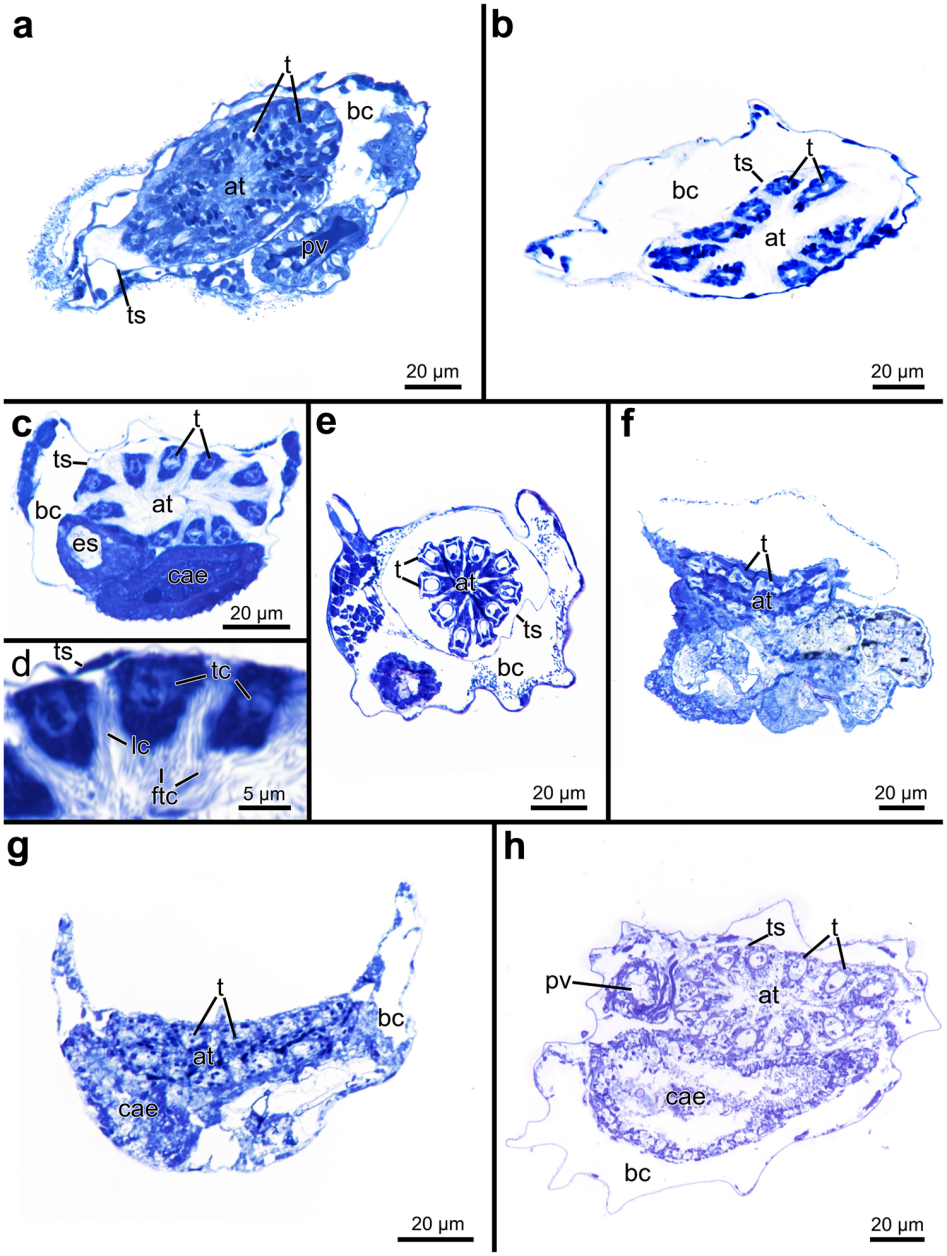
Histological semi thin cross sections through the lophophore and tentacles of the autozooids of six different penetrantiids. **a** *Penetrantia concharum* from Sweden. **b** *P.* cf. *concharum* from France. **c** *P. parva* from northern New Zealand. **d** Detail of cross section through tentacles of *P. parva* from northern New Zealand. **e** *P. parva* from southern New Zealand. **f** *P. irregularis* from southern New Zealand. **g** *P. clionoides* from Guam. **h** *Penetrantia* sp. from Japan. at atrium, bc body cavity, cae caecum, es esophagus, ftc frontal cilia, lc lateral cilia, pv proventriculus, t tentacle, ts tentacle sheath

The tentacle sheath following the diaphragm is very delicate and approximately half as long as the autozooid itself, lying somewhat diagonally within the body cavity in all penetrantiids. It enwraps the lophophore, which carries up to 14 tentacles. The number of tentacles differs between species but most have 10–12 (Table 3; Fig. 14). Intraspecific variation is present but low: *P. irregularis* may have 13 or 14 tentacles (Fig. 14f); *P.* cf. *concharum* from France can have nine or ten tentacles (Fig. 14b). A few autozooids of *P. parva* from northern New Zealand have nine tentacles too, but commonly possess between ten and 11 (Fig. 14c, e). Five species were observed to consistently have 12—*P. densa*, *P. brevis*, *P. operculata*, *P. clionoides*, and *Penetrantia* sp. from Japan (Table 3; Fig. 14g, h). The tentacles are triangular in cross section and about 5–10 µm wide. Cilia are present on the frontal and lateral sides. Abfrontal cilia were not observed (Fig. 14d).

#### Digestive tract

**Fig. 15:**
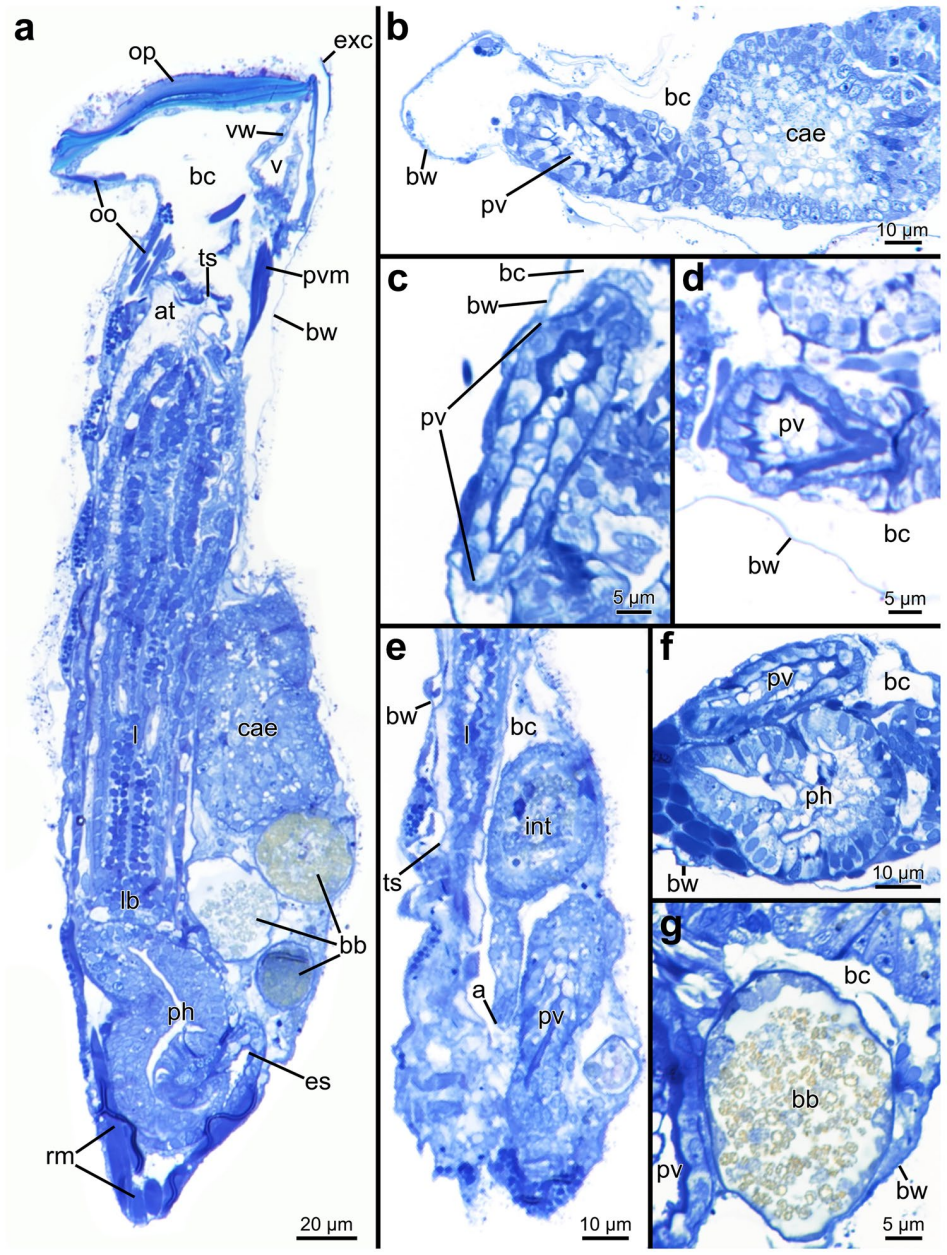
Histological semi thin sections of the digestive tract in *Penetrantia*. **a** Longitudinal section through an autozooid of *P. concharum* from Sweden. **b** Longitudinal section through the proventriculus and caecum of *P. concharum* from Sweden. **c** Proventriculus of *P. concharum* from Sweden. **d** Proventriculus *P.* cf. *concharum* from France. **e** Longitudinal section through intestine of *P. concharum* from Sweden. **f** Cross section through the pharynx of *P. concharum* from Sweden. **g** Cross section through a brown body in *P. concharum* from Sweden. a anus, at atrium, bb brown body, bc body cavity, bw body wall, cae caecum, es esophagus, exc exterior cuticle, int intestine, l lophophore, lb lophophoral base, oo operculum occlusor, op operculum, ph pharynx, pv proventriculus, pvm parieto-vestibular muscle, rm retractor muscle, ts tentacle sheath, v vestibulum, vw vestibular wall

**Fig. 16:**
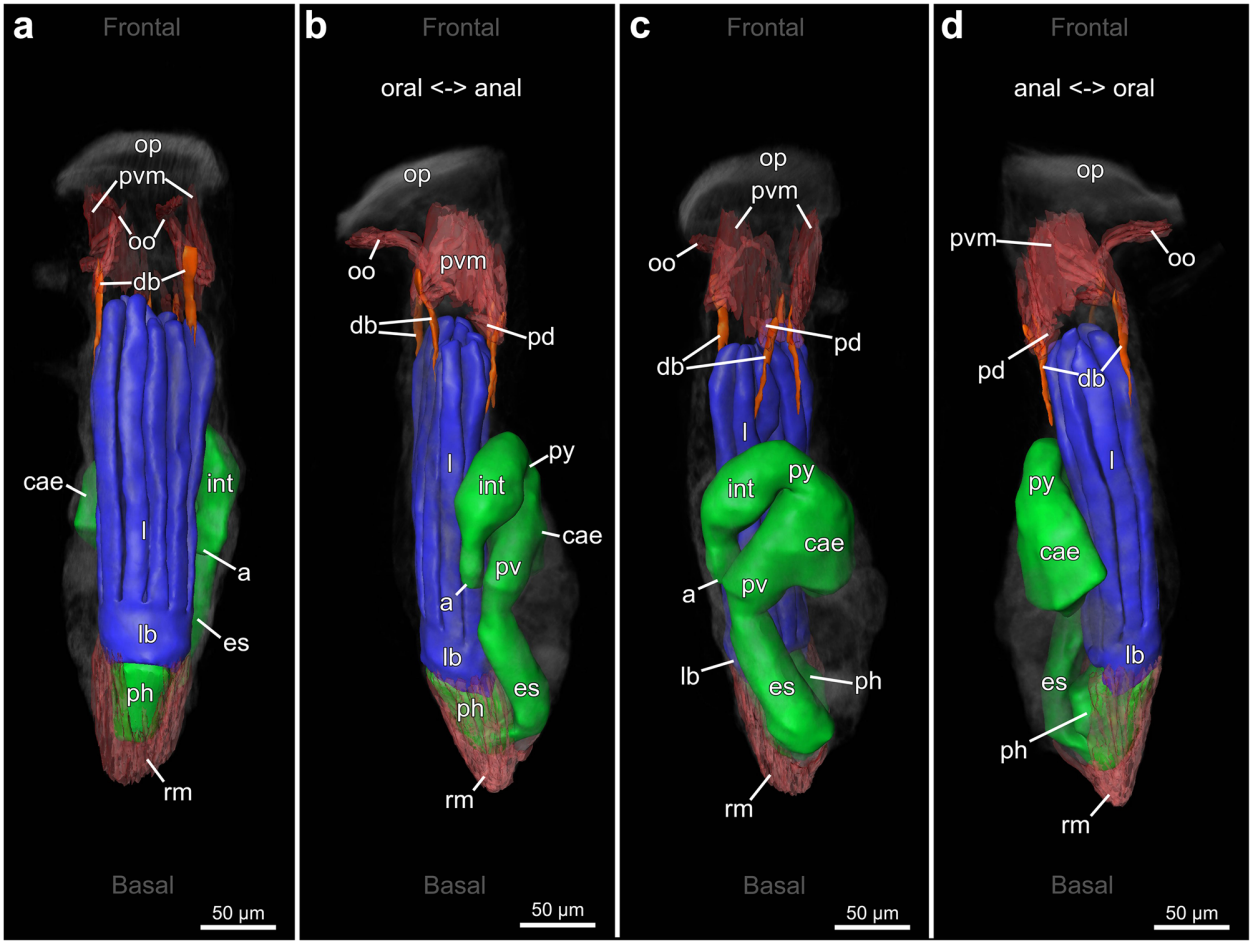
3D-reconstruction based on semi thin section series of *Penetrantia concharum* from Sweden. **a** Oral perspective. **b** Lateral perspective with the anal side on the right. **c** Anal perspective. **d** Lateral perspective with the anal side on the left. Blue: lophophore, green: digestive system, red: musculature, orange: duplicature bands. a anus, cae caecum, db duplicature band, es esophagus, l lophophore, lb lophophoral base, oo operculum occlusor, op operculum, pd parieto-diaphragmatic muscle, ph pharynx, pv proventriculus, pvm parieto-vestibular muscle, py pylorus, rm retractor muscle

**Fig. 17:**
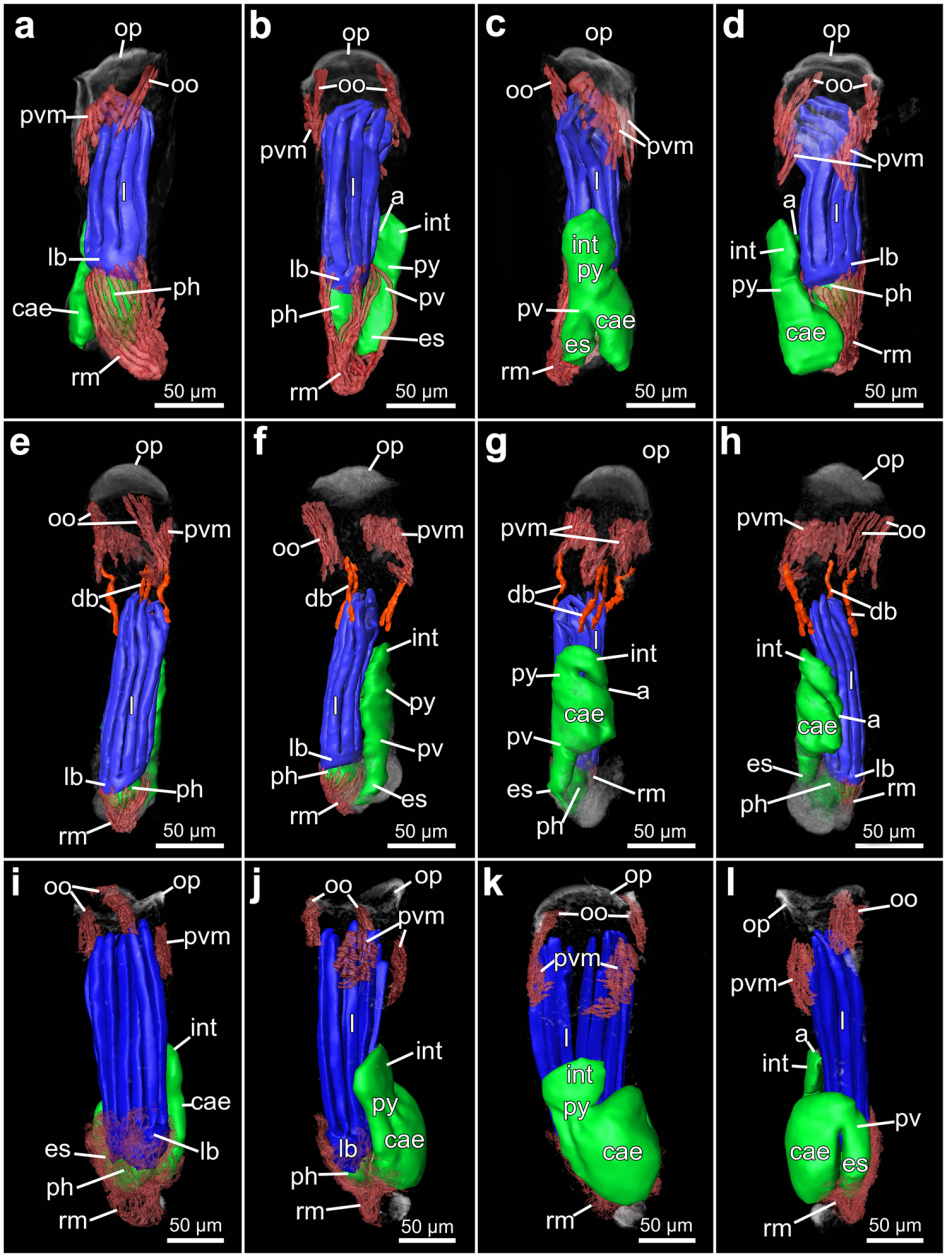
3D-reconstruction based on semi thin section series of three different penetrantiids. **a**–**d** *Penetrantia* cf. *concharum* from northern France. **e**–**h** *P. parva* from northern New Zealand. **i**–**l** *Penetrantia* sp. from Japan. **a**, **e**, **i** Oral perspective. **b**, **f**, **j** Lateral perspective with the anal side on the right. **c**, **g**, **k** Anal perspective. **d**, **h**, **l** Lateral perspective with the anal side on the left. Blue: lophophore, green: digestive system, red: musculature, orange: duplicature bands. a anus, cae caecum, db duplicature band, es esophagus, int intestine, l lophophore, lb lophophoral base, oo operculum occlusor, op operculum, ph pharynx, pv proventriculus, pvm parieto-vestibular muscle, py pylorus, rm retractor muscle

The digestive tract shows a very similar arrangement in all penetrantiids. At the center of the lophophoral base lies the circular mouth opening, which marks the beginning of the digestive system (Fig. 10). The digestive tract is u-shaped and is partitioned into three compartments: foregut (pharynx, esophagus), midgut (cardia, caecum, pylorus), and hindgut (intestine, anus). The pharynx starts just proximally of the mouth opening and proceeds further in a basal direction to the most basal part of the zooid. It is lined by a prominent myoepithelium (Fig. 15a), which forms a triradiate lumen in cross section and is funnel-shaped in longitudinal section (Fig. 15a, f). At the most basal point, the pharynx bends towards the anal side and proceeds in a frontal direction as the esophagus now until it reaches the cardiac valve (Figs. 10, 15a, 16 and 17). The cardia forms a small proventriculus about 20 µm long and 10–15 µm wide. Internally, its epithelium shows a smooth and thin cuticle, whereas externally it is surrounded by a cardiac constrictor (Figs. 10 and 15b–d). The cardia continues into the large bulbous and sac-like caecum, which occupies the majority of the space next to the lophophore (Figs. 10, 15a, 16 and 17). The caecum epithelium is equally thick and inconspicuous (Fig. 15a, b). Towards the orifice, the caecum transitions into the ciliated pylorus, the last part of the midgut. The adjoining intestine bends back basally and proceeds further in a basal direction until the level of the cardia where the anus enters the tentacle sheath. This entry point of the anus is situated at the basal portion of the tentacle sheath approximately at one-third of the length between lophophore base and diaphragm (Figs. 10, 15e, 16 and 17).

Brown bodies were observed in almost every investigated zooid of all penetrantiid species and are generally numerous; up to seven brown bodies were observed within one autozooid. *P. clionoides* and *Penetrantia* sp. from Japan were observed to only have a single brown body. They are most commonly found in the basal portion of the zooidal tube on the anal side of the polypide just next to the caecum. The brown bodies are of different sizes, of spherical shape, and enveloped in a membrane (Figs. 9 and 15a, g).

#### Autozooidial musculature

Fluorescence staining was carried out with specimens of *P. parva* from northern New Zealand, *P. concharum* from Sweden, and *P.* cf. *concharum* from France only. The musculature of all penetrantiid species investigated in this study shows strong similarities. Thus, the following descriptions apply to almost all investigated taxa; any species-specific differences are highlighted. As in all bryozoans, penetrantiids have different sets of muscles associated with different parts of their body: Apertural musculature, musculature associated with the polypide (lophophore, digestive tract, and retractor musculature), and body wall musculature (parietal musculature).

#### Apertural and tentacle sheath musculature

**Fig. 18:**
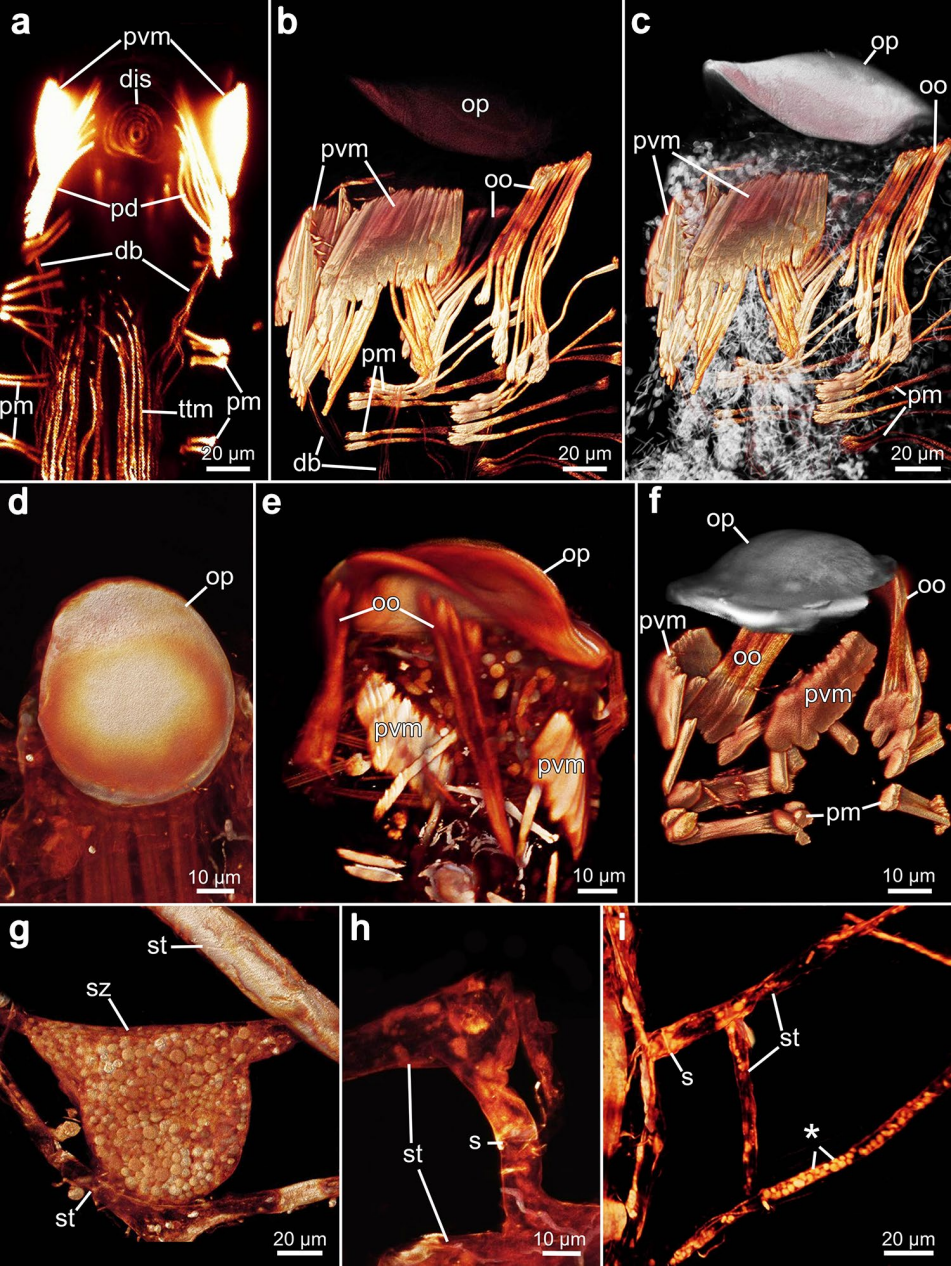
Apertural musculature, operculum, and stolon morphology of *Penetrantia.* **a**–**c** Musculature of aperture area in *Penetrantia parva* from northern New Zealand. **d** Operculum in *P.* cf. *concharum* from northern France. **e** Operculum and underlying musculature in *P. concharum* from Sweden. **f** Operculum and underlying musculature in *P. parva* from northern New Zealand. **g** Sac zooid in *P.* cf. *concharum* from northern France. **h**–**i** Stolonal network in *P.* cf. *concharum* from northern France. Asterisk indicates granules within a stolon. db duplicature band, dis diaphragmatic sphincter, oo operculum occlusor, op operculum, pd parieto-diaphragmatic muscle, pm parietal muscles, pvm parieto-vestibular muscle, s septum, st stolon, sz sac zooid, ttm tentacle muscles

The apertural musculature is associated with the orifice, bilaterally symmetrical, and consists of four sets of muscles: parieto-vestibular muscles, parieto-diaphragmatic muscles, operculum-occlusor muscles, and four duplicature bands (Figs. 10, 16 and 18). The vestibular wall itself lacks musculature. The paired parieto-vestibular muscles originate from the lateral body walls at the level of the diaphragm and attach along the entire length of the lateral sides of the vestibular wall (Fig. 18a–c). The parieto-diaphragmatic muscles have a similar origin, inserting at the diaphragmatic sphincter muscle (Fig. 18a). In all species investigated, the paired operculum-occlusor muscles originate from the lateral walls at the level of the tentacle sheath and extend to the oral side of the operculum (Figs. 10, 16, 17 and 18b, c, e, f).

**Fig. 19:**
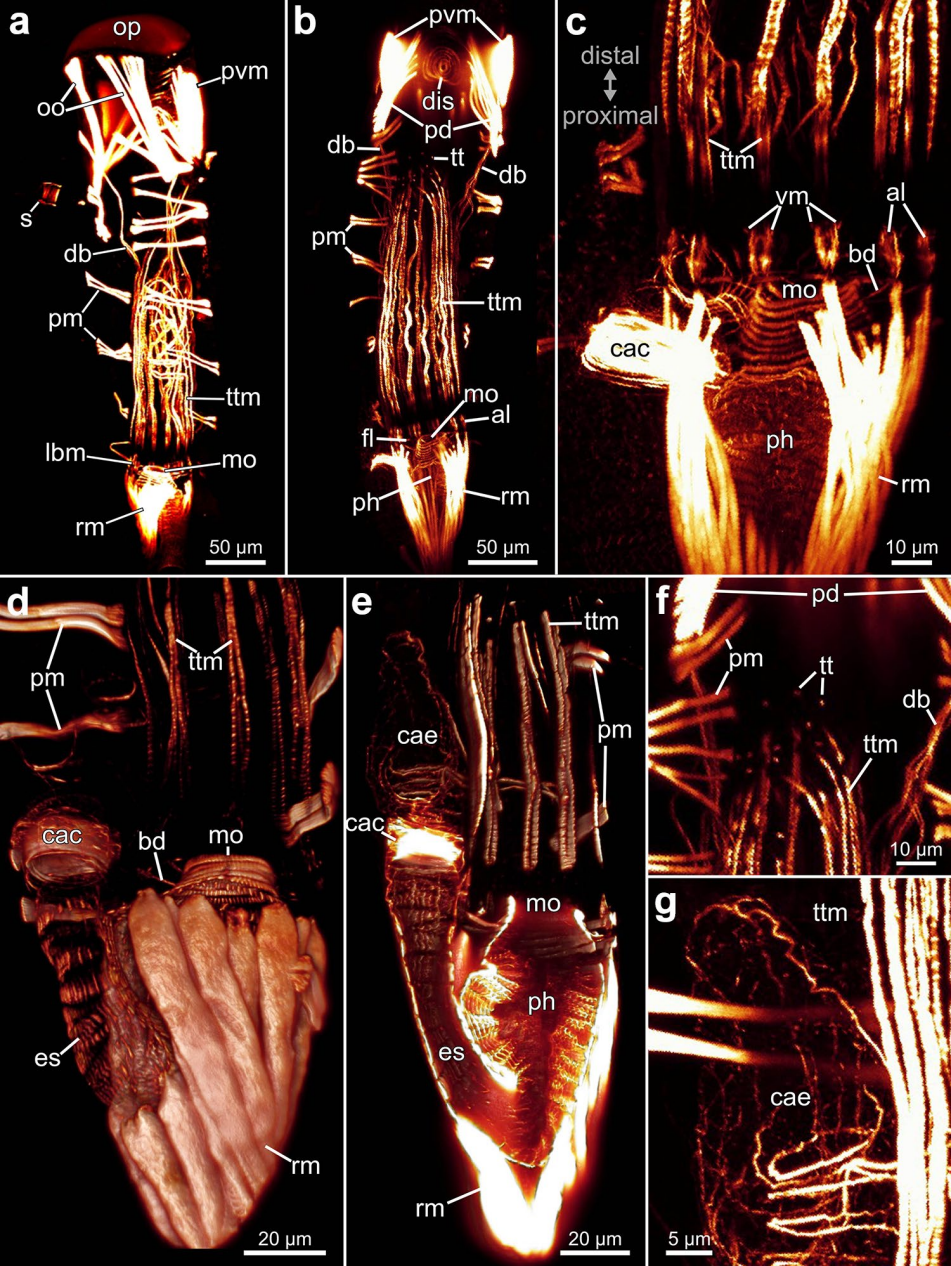
Musculature of the lophophore and digestive tract of *Penetrantia parva* from northern New Zealand. Each image is oriented with the frontal side upwards and the basal side downwards. **a**, **b** Overview of an entire autozooid. **c** Lophophore base. **d**–**e** Details of the basal part of the polypide. **f** Tentacle tip musculature. **g** Caecum musculature. al abfrontal lophophoral base muscle, bd buccal dilatator, cac cardiac constrictor, cae caecum, db duplicature band, dis diaphragmatic sphincter, es esophagus, fl frontal lophophoral base muscle, lbm lophophoral base muscles, mo mouth opening, op operculum, pd parieto-diaphragmatic muscle, ph pharynx, pm parietal muscles, pvm parieto-vestibular muscle, rm retractor muscle, s septum, tt tentacle tips, ttm tentacle muscle, vm “v” shaped lophophoral base muscles

A diaphragmatic sphincter is located at the basal end of the vestibulum and is composed of 5–7 delicate circular muscles. The diaphragmatic sphincter marks the transition from the vestibulum into the tentacle sheath, that houses the lophophore (Fig. 18a). The tentacle sheath itself has very delicate longitudinal muscle fibers, which extend from the diaphragm basally until the lophophore base. At the frontal margin of the tentacle sheath, some longitudinal muscle fibers coalesce and extend as duplicature bands frontally. A set of four duplicature bands was observed in *Penetrantia concharum* from Sweden and *P. parva* from northern New Zealand (Figs. 16, 17e–h, 18a and 19a, b).

#### Lophophore musculature

Since muscles associated with the lophophore traditionally termed according to the frontal and abfrontal side of the tentacles, these terms contradict the general body axis of penetrantiid zooids: The lophophore base is the proximal part while the tentacle tips are the distal part of the lophophore. The frontal side of the tentacles is facing inwards while the abfrontal side faces outwards of the lophophore.

The lophophoral base contains four sets of muscles: (1) A frontal lophophoral base muscle that encircles the inner side of the lophophoral base as an almost continuous bundle. (2) Buccal dilatators that run between each pair of tentacles from the pharynx outwards within the ring canal. Their number thus corresponds to the number of tentacles. (3) A longitudinal abfrontal lophophoral base muscle that runs on the outer side in proximo-distal direction of the lophophoral base. (4) The “v”-shaped muscles consisting of two (sometimes three) relatively small f-actin-rich elements on the disto-lateral side of each abfrontal lophophoral base muscle. These f-actin elements do not resemble a “v” (Fig. 19c).

Each tentacle is equipped with two cross-striated longitudinal muscle bundles, a frontal and abfrontal one. They commence approximately 10 µm distally of the abfrontal lophophoral base muscles and extend through almost the entire tentacle, ending approximately 10 µm below its tip (Fig. 19b–e). At each tentacle tip, f-actin-rich elements are present, sometimes in a circular arrangement (Fig. 19f).

#### Digestive tract musculature

Overall, the digestive tract is not heavily invested with muscles except for the pharynx and cardia region. A prominent myoepithelium lines the entire pharynx and shows cross-striated f-actin elements in the lateral sides of the epithelial cells and outer, surrounding circular musculature. The musculature of the esophagus has a similar appearance but possesses a much looser arrangement of muscles that becomes less prominent towards the cardia (Fig. 19e). The cardiac region itself has prominent circular musculature in the form of a cardiac constrictor (Fig. 19c–e). The caecum incorporates a mesh of delicate and inconspicuous circular as well as a few longitudinal muscle fibers (Fig. 19g). Even more delicate is the musculature of the hindgut, which is composed of a few longitudinal muscle fibers that proceed from the end of the pylorus over the entire length of the intestine.

#### Retractor musculature

The retractor muscle is the most prominent muscle in each zooid and enables a fast retraction of the polypide into the cystid. The retractor muscle fibers originate from the basal part of the zooid. They attach around almost the entire lophophore base, leaving only a gap on the anal side. A few additional muscle fibers of the retractor muscle attach at the basal part of the esophagus (Figs. 16, 17 and 19c–e).

#### Body wall musculature

The parietal musculature is part of the body wall musculature and facilitates the protrusion of the polypide. It is organized as paired bundles that traverse the body cavity from the anal to the oral side. Parietal muscles are distributed almost over the entire length of the zooid along the lateral body walls from the basal end of the vestibulum to the level of the lophophore base. Usually six to eight pairs are present on each lateral side, but the number can vary even among different individuals of the same species (Fig. 19a, b).

#### Stolonal musculature

There is no musculature within the stolons themselves, but f-actin-rich elements are present at the septa (Figs. 18h, i and 19a). In some parts of the stolonal network, granules are incorporated (Fig. 18i), which are of similar appearance to the granules within sac zooids (Fig. 18g).

#### Autozooidal nervous system

The same axis as described for the musculature associated with the lophophore will be used here (see chapter "Lophophoral musculature"). Useful data for nervous system staining were only possible with antibody staining against acetylated alpha-tubulin from *Penetrantia parva* of northern New Zealand. Thus, the following results relate to autozooids of this species only.

**Fig. 20:**
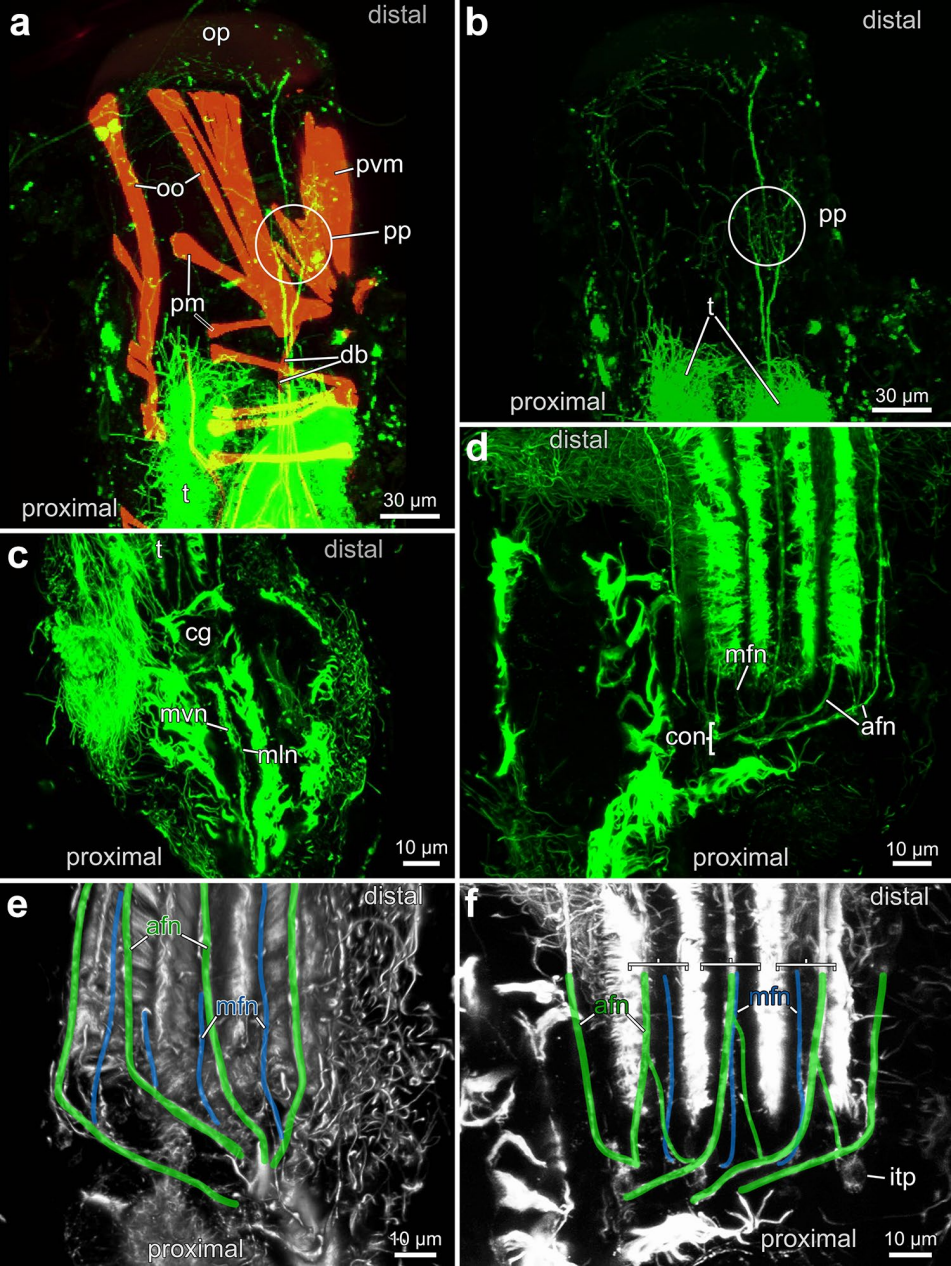
Nervous system of an autozooid in *Penetrantia parva* from northern New Zealand. Based on staining against acetylated alpha-tubulin indicated in green. **a**–**b** Apertural innervation. **c** Visceral innervation. **d** Lophophoral base innervation. **e**–**f** Tentacle innervation. afn abfrontal neurite bundle., cg cerebral ganglion, con circum-oral nerve ring, db duplicature band, itp intertentacular pit, mfn mediofrontal neurite bundle, mln medio-lateral visceral neurite bundle, mvn medio-visceral neurite bundle, oo operculum occlusor, op operculum, pm parietal muscles, pp parietal plexus, pvm parieto-vestibular muscle, t tentacle

The nervous system shows the highest accumulation of neurons in the cerebral ganglion on the anal side of the lophophoral base (Fig. 20d). From the cerebral ganglion, a circum-oral nerve ring encircles the mouth opening. From these two structures, the tentacle neurite bundles emerge. Each tentacle has two neurite bundles, a medio-frontal one along the frontal side and an abfrontal one on the opposite side. The medio-frontal neurite bundle branches off directly at the base of each tentacle, whereas the abfrontal neurite bundle root arises intertentacularly to fuse with its corresponding neighbor root (Fig. 20d–f).

From the proximal side of the cerebral ganglion, a medio-visceral neurite bundle and two medio-lateral visceral neurite bundles proceed basally along the pharynx (Fig. 20c).

Along the duplicature bands, neurite bundles proceed distally as extensions of parts of the compound tentacle sheath neurite bundle. The exact origin could not be observed in this study. The neurite bundles associated with the duplicature bands on the anal side separate further into two main branches. One of the branches innervates the parieto-vestibular musculature; the second branch is continuous in distal direction towards the diaphragmatic sphincter. The neurite bundles associated with the duplicature bands on the oral side terminate at the basal part of the operculum and areas of the vestibular wall (Fig. 20a, b).

### Gonozooid morphology

#### Gross morphology

**Fig. 21:**
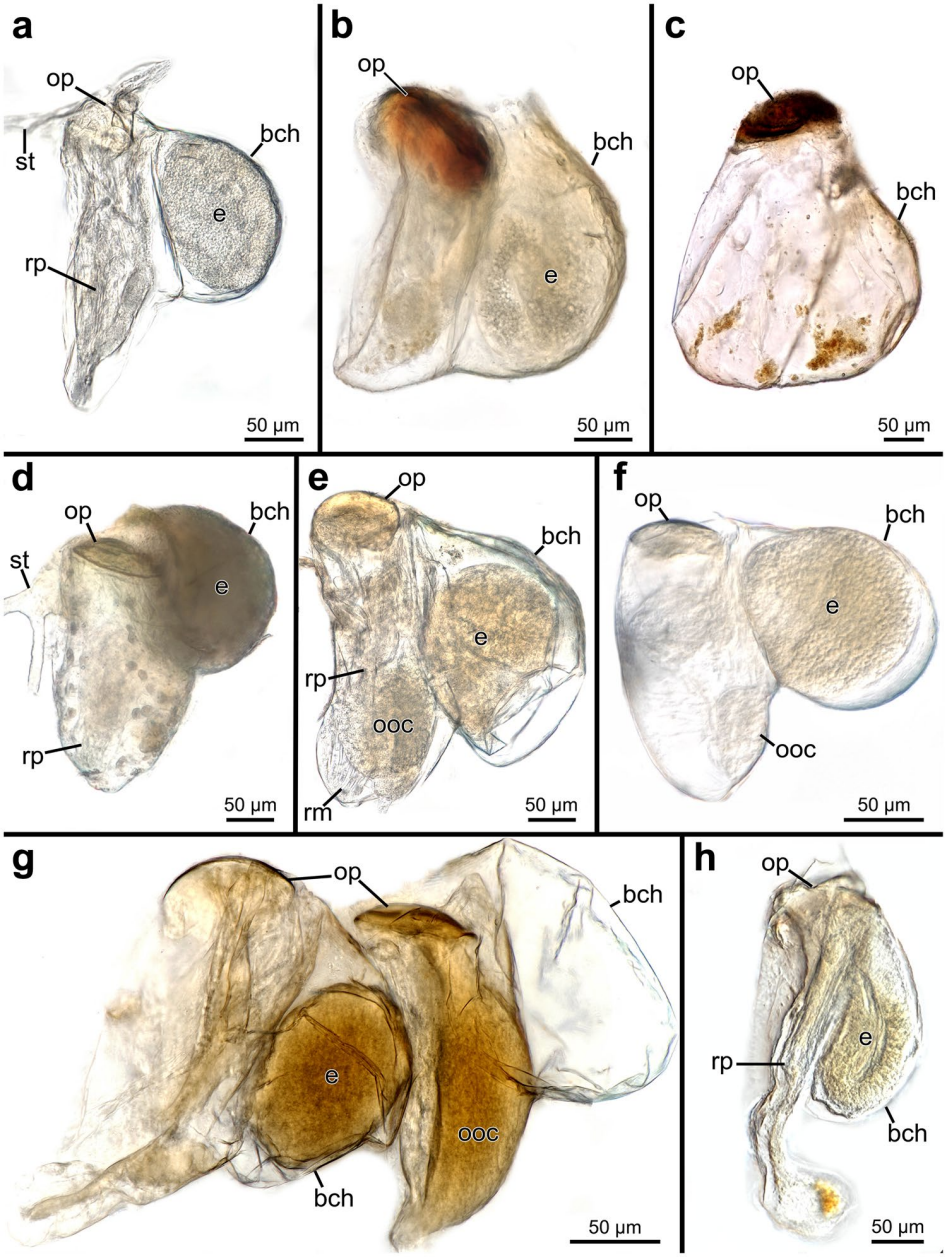
Stereomicroscopic images of whole mounts of gonozooids of six different penetrantiids. **a** *Penetrantia concharum* from Sweden. **b**–**c** *P. irregularis* from southern New Zealand. **d** *P. parva* from northern New Zealand. **e** *P. parva* from southern New Zealand. **f** *P.* cf. *parva* from New Caledonia. **g** *P. clionoides* from Guam. **h** *Penetrantia* sp. from Japan. bch brood chamber, e embryo, ooc oocyte, op operculum, rm retractor muscle, rp remains of polypide, st stolon

**Fig. 22:**
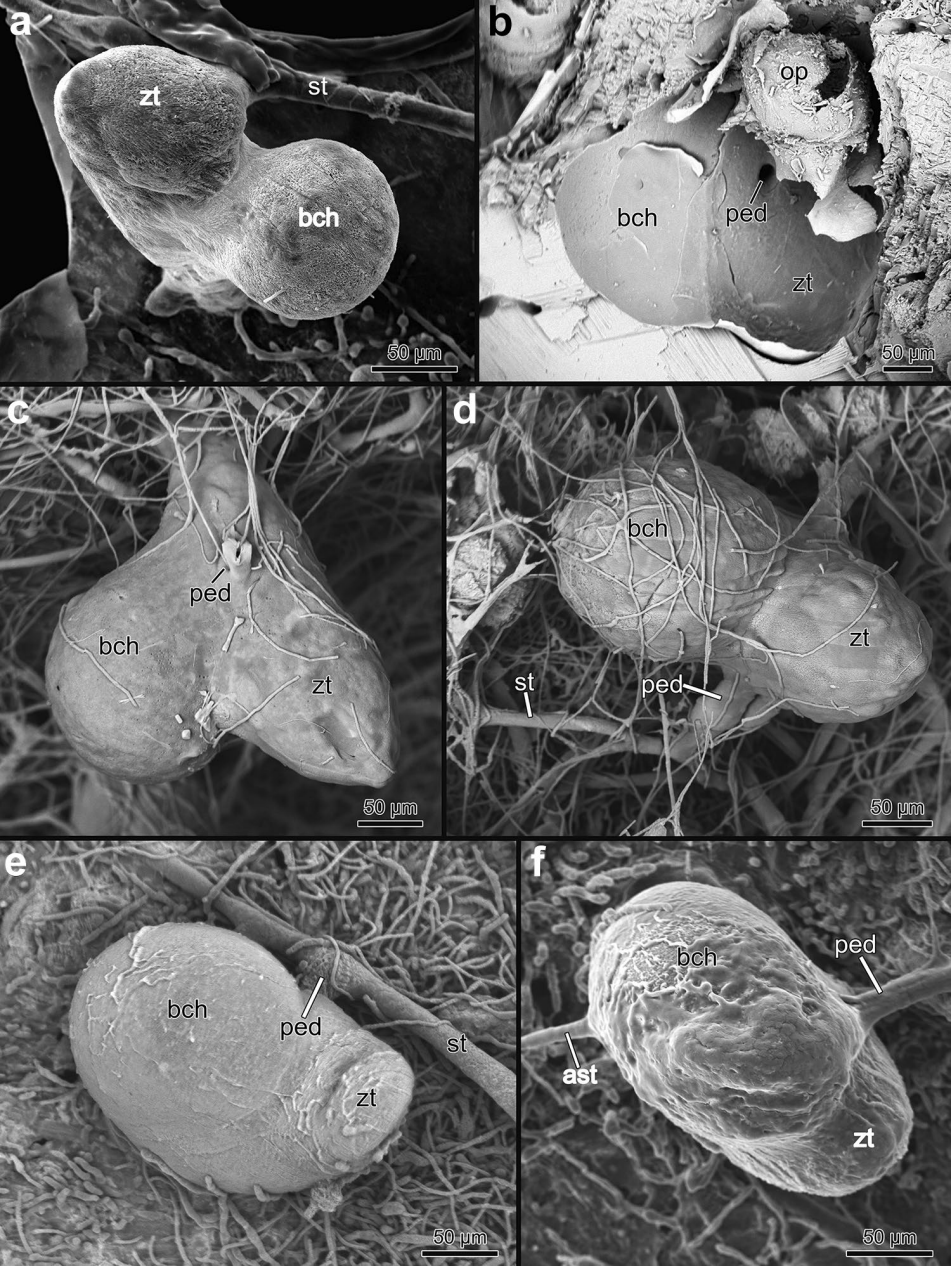
Scanning electron microscopic images of resin casts of gonozooids in four different penetrantiids. **a** *Penetrantia* sp. from Spain. **b** *P. irregularis* from southern New Zealand. **c**–**d** *P. parva* from northern New Zealand. **e**–**f** *Penetrantia* sp. from Japan. ast adventitious stolon, bch brood chamber, op operculum, ped peduncle, st stolon, zt zooidal tube

Penetrantiids have gonozooids that are typically half to two-thirds the size of the corresponding autozooids, 201–600 µm in length. Only gonozooids of *Penetrantia densa* and *Penetrantia* sp. from Japan are the same size as their autozooids. *P*. *densa* has by far the largest gonozooids with a length of about 600 µm followed by *Penetrantia* sp. from Japan with a mean length of 390 µm. *P. sileni* has the smallest gonozooids with a mean length of 201 µm. Most species have gonozooids with a mean length between 225 and 345 µm, but detailed information is missing for several species (*P. densa*, *P. concharum* from Norway, *P. brevis*). Not a single gonozooid has been observed in *P. taeanata* and *P. bellardiellae* (Table 3; Fig. 11)*.* The largest differences in size between the gonozooids of *P. parva* from southern New Zealand (mean length: 345 µm), *P. parva* from northern New Zealand (mean length: 280 µm), and *P.* cf. *parva* from New Caledonia (mean length: 221 µm) (Table 3; Figs. 11 and 21). Gonozooids have a typical spherical brood chamber on their anal side (Fig. 10c) and two different basic types can be distinguished:

- Type 1: the brood chamber is as long as the gonozooid itself and the basal part of the zooidal tube does not reach below the brood chamber. This condition was observed in *P. brevis*, *P. irregularis*, and in *P. operculata* (Figs. 11c, f, g, 21b, c and 22b).
- Type 2: The brood chamber is about half as long as the gonozooid itself, and the basal part of the zooidal tube reaches below the brood chamber. This type occurs in all remaining penetrantiid species (Figs. 11a, b, d, e, h, k, 21a, d–h and 22a, c, d, e). The basal extension of gonozooids can differ between species. It can be relatively long and slender and bent towards the brood chamber, as in *Penetrantia* sp. from Japan (Figs. 11k, 21h; and 22e) or shorter and pronounced, as in *P. parva* from northern New Zealand, *P. parva* from southern New Zealand, and *P.* cf. *parva* from New Caledonia (Figs. 21d–f and 22c). In *P. clionoides*, this basal extension of the gonozooids proceeds to the oral side away from the brood chamber (Figs. 11h and 21g). The chambers themselves are either globular as in *P. concharum*, *P.* cf. *concharum* from France, *Penetrantia* sp. from Spain, *P. parva* from southern New Zealand, and *P.* cf. *parva* from New Caledonia (Figs. 11b, d, 21a, d–f and 22a, c, d) or more pear-shaped in longitudinal section as in *P. densa*, *P. brevis*, *P. irregularis*, *P. clionoides*, and in *Penetrantia* sp. from Japan (Figs. 11a, c, f, h, k, 21b, c, g, h, and 22b, e).

#### Soft body morphology

The opercula of all investigated gonozooids corresponds in size, shape, and orientation to the respective autozooid of a given species. The only difference was observed between autozooids and gonozooids of *P. concharum* from Sweden, where opercula of the gonozooid are on average 20 µm smaller in diameter (Table 3; Fig. 21).

**Fig. 23:**
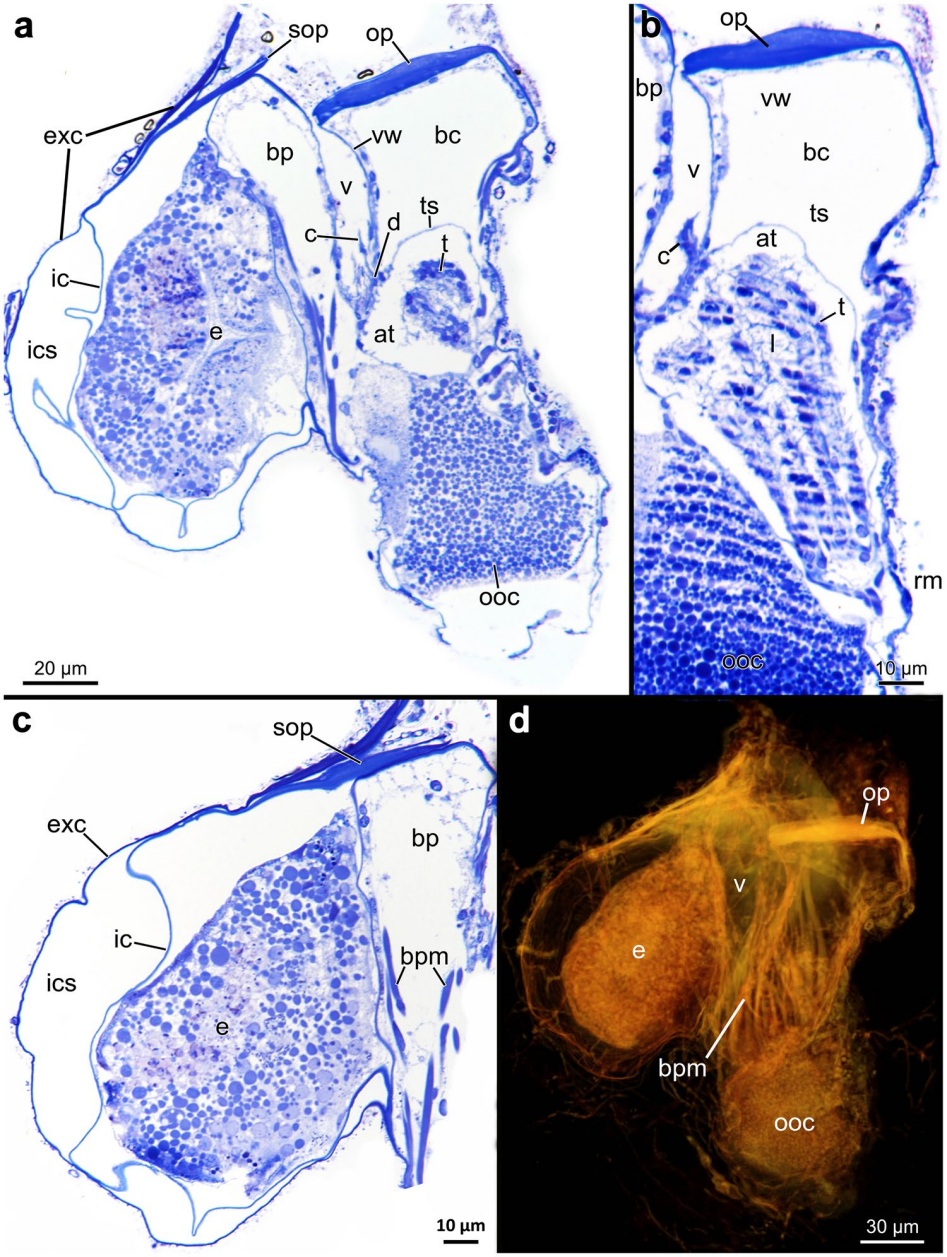
Histology of the gonozooid in *Penetrantia concharum* from Sweden. **a**–**c** Longitudinal semi thin sections through a gonozooid with the brood chamber and anal side on the left. **b** Close-up of lophophore and tentacles. **c** Close-up of brood chamber and embryo. **d** Volume rendering based on a section series with the anal side on the left. at atrium, bc body cavity, bp brood chamber plug, bpm brood chamber plug muscle, c collar, d diaphragm, e embryo, exc exterior cuticle, ic interior cuticle, ics intercuticular space, l lophophore, ooc oocyte, op operculum, rm retractor muscle, sop secondary operculum, t tentacle, ts tentacle sheath, v vestibulum, vw vestibular wall

The unique double cuticle, with an interior and exterior cuticle, is also present in gonozooids of all investigated species. This feature is particularly apparent in the apertural area, at the opening of the brood chamber, of *P. concharum* from Sweden. Both cuticles line the brood chamber and split in the area close to the orifice, where they are more strongly cuticularized. The exterior cuticle proceeds further frontally to line the borehole, while the inner cuticle acts as a secondary operculum for the brood chamber. At the basal end of the brood chamber, both cuticles fuse again and line the rest of the body wall (Fig. 23a, c).

The orifice sits on the anal side of the gonozooid between operculum and brood chamber. The vestibular wall between vestibulum and brood chamber forms a plug and effectively seals the brood chamber. It extends further frontally than the operculum, as described for autozooids but more prominently (Figs. 10c and 23a, b, d). At the basal part of the vestibular wall, an inconspicuous collar is sometimes present on the diaphragm (Fig. 23a, b).

**Fig. 24:**
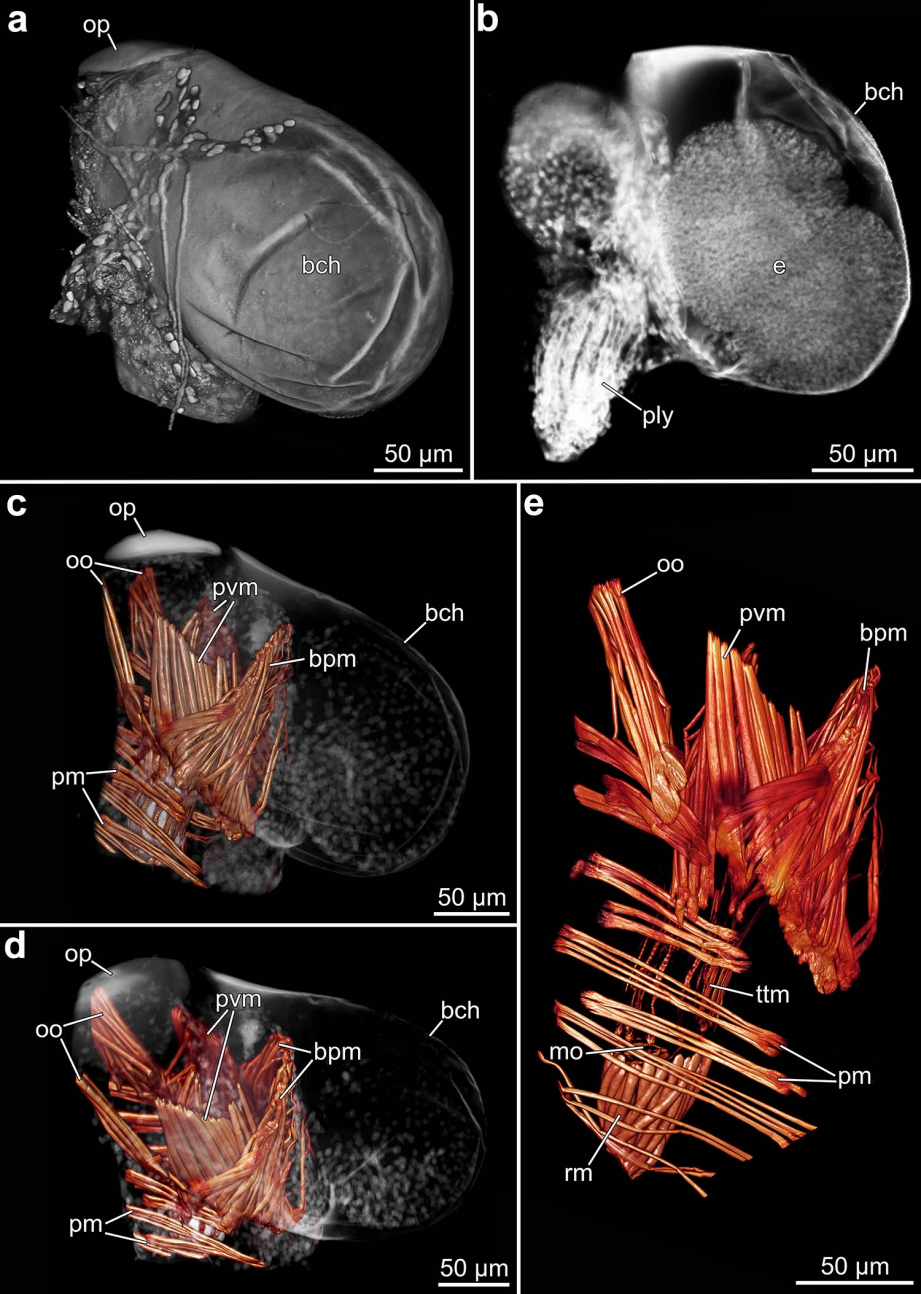
Musculature of the gonozooid in *Penetrantia parva* from northern New Zealand. **a** Overview of the gonozooid and brood chamber and their exterior cuticle. **b** Polypide within zooidal tube and embryo within brood chamber. **c**–**d** Musculature of gonozooid and brood chamber. Note: the additional brood chamber plug muscle. bch brood chamber, bpm brood chamber plug muscle, **e** embryo, mo mouth opening, oo operculum occlusor, op operculum, ply polypide, pm parietal muscles, pvm parieto-vestibular muscle, rm retractor muscle, ttm tentacle muscle

Functional lophophores were present in gonozooids of *P. concharum* from Sweden and *P. parva* from northern New Zealand (Figs. 23b and 24b, e). In most gonozooids, the polypide is reduced or completely absent (as in some brooding gonozooids of *P. clionoides*) (Fig. 21g). When present, the lophophore is similar to the corresponding autozooid, but generally smaller in size with fewer tentacles. *P. concharum* from Sweden and *P. brevis* have eight tentacles, and the gonozooids of *P. parva* from northern New Zealand have ten tentacles (Table 3).

A digestive system was observed in gonozooids of *P. concharum* from Sweden, but the midgut and hindgut are dramatically reduced in size, rendering its functionality questionable (Fig. 23b). Functional lophophores with tentacles and muscular pharynx were observed in gonozooids of *P. parva* from northern New Zealand, but information on its mid- and hindgut is missing as well (Fig. 24e).

#### Gonozooid musculature

Overall, the musculature of all investigated gonozooids (*Penetrantia concharum* from Sweden and *P. parva* from northern New Zealand) is similar to the corresponding autozooids. The musculature is miniaturized in most cases, too, except for the apertural musculature. In addition to the regular muscle system of autozooids, an additional set, the plug retractor muscle, is associated with the vestibular wall of the brood chamber (Figs. 10c and 24c–e).

As in the autozooids, three sets of paired muscle bundles are present in the apertural area of gonozooids: parieto-vestibular muscles, parieto-diaphragmatic muscles, and a set of operculum occlusor muscles. The arrangement is identical to the corresponding autozooid. The plug-retractor muscle appears similar to the parieto-vestibular muscles with an origin from both lateral body walls at the basal end of the vestibular wall. Contrary to the parieto-vestibular muscles, the plug retractor runs to the anal side of the vestibular wall to insert at the frontal end of the brood plug (Figs. 10c and 24c–e).

The musculature associated with the polypide corresponds to the autozooids except for: (1) musculature or f-actin-rich elements in the tentacle tips were not observed and (2) digestive tract musculature is strongly reduced and even the prominent cardiac musculature is absent (Fig. 24c–e).

#### Brooding

Oocytes, early embryos, and larvae were observed only in gonozooids. Embryos and larvae were observed exclusively within the brood chamber, and the high concentration of yolk indicates that larvae are lecithotrophic of the coronate type (Figs. 21a, b, d–h, 23a, c, d and 24b). Unfertilized oocytes of different sizes containing different amounts of yolk were observed in the body cavity of the gonozooid in several species (*Penetrantia concharum* from Sweden, *P. parva* from New Zealand, *P. irregularis*, and *P. clionoides*) (Figs. 21e, g and 23a, b).

#### Emended diagnosis of family Penetrantiidae

Colonies, commonly found in shells of molluscs, completely immersed into substrate with borehole openings and apertures, as distinct traces. Apertures often D- or kidney-shaped, 70–126 µm in width. Zooids placed vertically in substrate on both lateral sides of interconnecting kenozooidal stolons. Stolonal network feather-shaped or mesh-like with principal stolon and secondary branches, often orthogonal on both lateral sides of principal stolon—crisscross-like. Stolons with tubulets in different intervals: 40–235 µm, creating openings on surface of substrate: 5–10 µm. Sac zooids sometimes present bag-like extensions of stolon. Zooids always connected by lateral peduncle to stolonal network. True polymorphic gonozooids with external brood chamber. All zooids with cuticularized or partially calcified operculum countersunk in borehole. Body wall with double cuticular lining, interior and exterior cuticle, particular prominent in frontal area. Autozooids: tubular shaped 160–700 µm in length, 50–148 µm in width, tentacles 9–13, four sets of apertural muscles, small proventriculus. Gonozooids: shorter than autozooids 201–390 µm in length, 161–292 µm in width, tentacles 8–10, reduced polypide, five sets of apertural muscles with paired brood chamber plug muscle, spherical brood chamber sometimes with secondary operculum.

## Discussion

### Biogeography and distribution

The family Penetrantiidae exhibits an almost cosmopolitan distribution, with higher diversity in tropical regions, where the majority of penetrantiid species occur, although the distribution extends into the temperate seas of the Northern and Southern Hemispheres. However, the data on the geographical range of certain species rely on few accounts and are unlikely to represent the entire distribution of a species (Pohowsky, 1978; Soule & Soule, 1969a). The absence of *Penetrantia* records from polar and subpolar regions could indicate a latitudinal limit in the distribution, an idea supported by the lack of borings in a large amount of material from three different localities within the polar and subpolar regions. Most reported species occur exclusively in a rather small area, and few have been reported to have an overlapping distribution. For example, two penetrantiid species are known to co-occur in New Zealand (*P. parva* and *P. irregularis*) and can be found within the same shell, as shown herein. Consequently, species identification needs to be carefully conducted (Gordon, 1986). While *P. irregularis* is restricted to New Zealand, *P. parva* was also reported from Hawaii where it probably co-occurs with *P. operculata* (Gordon, 1986; Silén, 1946, 1956; Soule & Soule, 1969b). However, *P. parva* might resemble a complex of cryptic species, as very similar specimens from New Caledonia showed minor, but distinct morphological differences. Such differences were also observed between specimens from northern and southern New Zealand (see section of "Autozooidal morphology").

Three different penetrantiid species have been reported from the Pacific coasts of California and Mexico, but there is some doubt about species identity especially for records of *P. concharum* (Soule, 1950; Soule & Soule, 1969b). This species was originally described from Sweden and otherwise seems restricted to Northern Europe (Silén, 1946). Since all analyzed penetrantiids most likely have short-lived lecithotrophic larvae, such a broad distribution seems very unlikely. Nevertheless, another predominantly Atlantic species, *P. densa*, was also described from the Californian and Mexican Pacific coasts. This identification seems more likely since *P. densa* was described originally from South Africa and Caribbean Panama. An expansion of its distribution through the Panama Canal is plausible and would result in the broadest geographical distribution of all penetrantiids (Silén, 1946; Soule, 1950). Only future sampling along the Californian and Mexican Pacific coasts can shed light on the true distribution, particularly of *P. concharum*.

The true distribution of *P. concharum* in Europe also remains questionable. While it has been reported from many locations in the North Sea, specimens collected from the Atlantic coast of northern France show some morphological differences (see section on "Autozooidal morphology") and might indicate a cryptic species. *Penetrantia* was previously reported from this area, but not identified at the species level (Reverter-Gil et al., 1995). There are additional reports of penetrantiids in European waters along the Iberian coast, which were considered to be neither of the two European penetrantiids (*P. concharum* or *P. brevis*) and might represent undescribed species (Reverter-Gil & Suoto, 2014; Reverter-Gil et al., 2016). These findings may parallel the potentially cryptic species from northern France or represent yet another unknown species.

This study includes the very first report of a penetrantiid species in Japan, also the first report of any boring bryozoan from Japan. As this penetrantiid species does not correspond to *P. clionoides* from Guam or *P. taeanata* from South Korea (see section on "Autozooidal morphology"), which are the closest penetrantiid locations to Japan, this species probably represents an undescribed species awaiting description.

There are still many open questions about the true distribution of most penetrantiid species, as well as on the co-occurrence of cryptic species. However, this study expands the knowledge on the distribution of penetrantiids, which is important information particularly for species identification. Sequencing of certain markers like cytochrome C oxidase subunit and ribosomal RNA subunits of various populations from different areas will shed more light on this issue (e.g., Baptista et al., 2022).

Additionally, there are more boring traces which probably belong to *Penetrantia* in recent and fossil material and could add information about their geographic as well as stratigraphic distribution (Pohowsky, 1978). However, since most of these records are boring traces with no information about important diagnostic soft body features, it is not possible to assign these reports to penetrantiids with certainty. The same holds true for both fossil *Penetrantia* species (*Penetrantia goasaviensis* Voigt & Soule, 1973 and *Penetrantia soulei* Pohowsky, 1978), which represent trace fossils with no information about soft body parts (see Voigt & Soule, 1973; Pohowsky, 1978) and may be reassigned from the penetrantiids as separate ichnotaxa similar to *Iramena* (see Boekschoten, 1970).

### Biology and substrate diversity

All boring bryozoan taxa seem to be restricted to calcified substrates, in particular biogenic calcium-carbonate structures, such as shells of molluscs, as reported in the current and all previous studies. As microeroders, endolithic boring bryozoans, including species of the genus *Penetrantia*, probably use chemical dissolution of the calcareous substrate (Schönberg et al., 2017). Mechanical erosion can be ruled out for the family Penetrantiidae and probably for all endolithic boring bryozoans since no organs associated with mechanical removal of substrate are present. A mechanical erosion apparatus is only known for removal of softer material such as polychaete tubes, as in the ctenostome *Hypophorella expansa* (Pröts et al., 2019). It has been previously suggested that *Penetrantia densa* applies phosphoric acid to dissolve calcareous substrates, but this hypothesis was not supported by subsequent experiments (Pohowsky, 1978; Silén, 1947).

Penetrantiids seem highly adapted to use molluscan shells, since the majority of specimens colonize shells of dead and living molluscs. Given the sheer abundance and diversity of marine molluscs, using molluscan shells would seem advantageous (Pohowsky, 1978). However, penetrantiids are not restricted to molluscan shells and at least a few species retain a flexibility in their substrate preferences, since they were occasionally observed in calcified structures of other phyla as well. Penetrantiids seem to prefer dead shells over the shells of living molluscs. Shells of certain molluscan species appear not to be colonized at all (e.g., the gastropods, *Phorcus lineatus*, and *Steromphala cineraria*), which suggests that different shell compositions are critical for substrate selection. Similar substrate preferences were previously observed before and some might be even species-specific, indicating that certain penetrantiids might be adapted to certain shell types (Pohowsky, 1978; Silén, 1947).

Molluscan shells possess an outer protective organic layer, the periostracum, which makes colonization more difficult, particularly of living molluscs (Marin et al., 2012; Pohowsky, 1978). Indeed, penetrantiids tend to colonize older shell parts of living gastropods, where the periostracum is partly removed, and access to the prismatic layer is unimpeded. Moreover, areas close to the aperture of the gastropod shell where the periostracum is presumably weaker are also targeted by penetrantiids (Silén, 1947).

Interestingly, there is only a single report of a penetrantiid occupying a living bivalve. *Penetrantia irregularis* was once observed to colonize shells of the living mytilid *Perna canaliculus* (Silén, 1956). In dead bivalves, the lack of internal soft tissue gives access to the inner shell surface, which does not feature a periostracum. Possibly, the periostracum is impenetrable when the bivalve is alive, and colonization is only possible after its death. Most bivalve shells were in fact colonized on their inner side; only in older and more eroded shells were the outer surfaces also penetrated.

Overall, a higher number of gastropod than bivalve species are recorded as colonized by penetrantiids. This difference could have several reasons: (1) Species richness: Gastropoda has a much higher species richness than Bivalvia and therefore more species as potential substrate. (2) Sampling bias: *Penetrantia clionoides* from Guam was found in 40 different gastropod species alone, which are two-thirds of all reported colonized gastropod species (Smyth, 1988). However, even if more gastropod species are suitable as substrate, the number of colonized bivalve shells was much higher, especially in subtidal habitats. More comprehensive studies are required to get a better understanding of substrate preferences in penetrantiids, particularly in subtidal habitats. So far, the vertical distribution of penetrantiids ranges from the intertidal into the subtidal zone as deep as 400 m, with the majority being restricted to the intertidal and only a few species in both zones.

Altogether, further information on substrate preferences and vertical distribution limits will shed more light on global distribution patterns of certain penetrantiids and provide additional information for species identification.

### Borehole apertures

The borehole apertures of penetrantiids are distinctive and often serve as a diagnostic character to distinguish them from other boring bryozoans. In some cases, they give a first indication of species identity, which is particularly helpful in the field since these apertures are usually the only external signs of the presence of a boring bryozoan colony (Silén, 1947). Most penetrantiids have kidney-shaped apertures with the operculum being somewhat countersunk (Pohowsky, 1978; Silén, 1946, 1947). Younger apertures of live *Penetrantia concharum* are kidney-shaped with the free margin of the operculum being on the curved side which corresponds to the anal side of the zooid, while the opposing oral side of the aperture is slightly bent (Silén, 1946). In this study, colonies of *P.* cf. *concharum* from France and Norway show very similar apertures to *P. concharum* from Sweden.

In older parts of a colony or in dead colonies, however, the shape can be more circular making comparisons difficult. The alteration of apertures by abrasion is a common problem in the identification of boring bryozoans, especially in genera where zooids are positioned horizontally close to the surface of the substrate (e.g., *Terebripora*). The destruction of the thin surface layer of the substrate can change the outline of the aperture dramatically (Pohowsky, 1978).

The most distinct apertures of the penetrantiids we examined are found in *P. parva* from New Zealand. On the oral side of the aperture, some hinge-like notches or median cusps are commonly found (Gordon, 1986). These apertural notches were considered to resemble a lyrula and lateral cardelles, as found in some cheilostome bryozoans (Pohowsky, 1978; Soule & Soule, 1969a). However, these peculiar apertural notches are the remains of the substrate and are formed indirectly by dissolution of the substrate and are thus not part of the zooid. In cheilostomes such denticles, including the lyrula, are part of the zooid itself and formed by, for example, gymnocystal calcification. However, the lyrula and other supraopercular orificial structures of cheilostomes are not necessarily homologous to those observed in *P. parva*, and may have evolved independently several times (Berning et al., 2014).

The function of apertural notches or denticles remains unclear and might not be crucial since this structure is less prominent in *P. concharum* and, indeed, absent in most penetrantiids. Nonetheless, such characteristic apertural notches serve as diagnostic features to distinguish the *P. parva* species complex from other penetrantiids. However, as mentioned, abrasion can likewise alter the shape to be kidney-shaped and circular. Based on this peculiar feature, it is very likely that specimens with similar apertures from the North and South Islands of New Zealand as well as from New Caledonia are the same or closely related species. The only other species with similar apertural notches and similar-sized apertures is *P. operculata* from Hawaii, which otherwise is easily distinguished by its knobbed operculum (Soule & Soule, 1969b).

The second penetrantiid species found in New Zealand, *Penetrantia irregularis*, shows significant differences in its aperture outlines when compared to *P. parva*. The apertures differ in size and shape and apertural notches are always absent. These differences were previously reported and are a very helpful feature to distinguish the two species, particularly in the field (Gordon, 1986).

A calcareous apertural rim surrounding the aperture was reported for *Penetrantia densa* (Silén, 1947)*,* but could not be found in any species in this study*.* This apertural rim may result from erosion, as the substrate surrounding the zooids may be reinforced. If the remaining substrate erodes faster, raised rims will be left around the apertures (Silén, 1947). A second possibility could be the secretion of a calcareous layer by the zooid itself creating the raised apertural rim (Voigt & Soule, 1973). The latter hypothesis would be intriguing for a ctenostome bryozoan, since no other ctenostome is known to secrete calcium carbonate or to have a mineralized cuticle, otherwise typical for cheilostomes among Gymnolaemata (Schwaha, 2020b). Interestingly, similar apertural rims were reported in a few specimens of *P. clionoides* and *P. bellardiellae.* They are uncommon in both species, but, when present, the apertural rim is clearly separated from the remaining substrate by a small gap and often raised above the surface, which suggests either the secretion of calcareous material or the remodeling and displacement of dissolved calcium carbonate from the aperture itself (Schwaha et al., 2019a; Smyth, 1988).

A few apertures of *Penetrantia* sp. from Japan, *P. clionoides* and *P. bellardiellae*, are also key-hole shaped, with a sinus-like cutout or opercular articulation on the oral side of the aperture (Schwaha et al., 2019a; Smyth, 1988). This shape might also be the remains of the substrate in younger apertures or secretion of some calcareous material. Since the opercula of *P. densa*, *P. clionoides*, *Penetrantia* sp. from Japan, and most likely *P. bellardiellae*, are at least partially composed of calcium carbonate, there must be an underlying mechanism of calcification at least in these species (Pohowsky, 1978; Schwaha et al., 2019a; Smyth, 1988).

### Colony and stolon morphology

Since stolons are clearly separated from the autozooids and gonozooids by interzooidal septa in all penetrantiids, they are considered true polymorphic and individual kenozooids (Schack et al., 2019). True kenozooidal stolons are otherwise only known from the Walkerioidea and Vesicularioidea among ctenostomes (Jebram, 1973; Schwaha, 2020d). Dense stolonal networks increase the interconnectivity within the colony, enable distribution of metabolites and nutrients throughout the colony, and play a crucial role in the asexual proliferation of the colony (Mukai et al., 1997; Pohowsky, 1978). Considering the gross colony structure, penetrantiid colonies show a close resemblance to members of the Walkerioidea, such as *Mimosella bocki* (Silén, 1942) or *Farrella repens* (Farre, 1837). However, walkerioideans have transverse stolonal muscle fibers at the distal end of their stolons and kenozooidal interzooids between the stolons and autozooids, both features are absent in penetrantiids (Jebram, 1973; Schwaha & Wanninger, 2018; Schwaha, 2020d). Unique are pedunculate zooids which are also known from the walkerioideans *Farella repens* and the family Triticellidae. However, in all penetrantiids, the peduncle develops on the lateral side in the frontal area of the zooidal tube, whereas the peduncle in *Farella repens* and triticellids is a basal extension of the zooid (Jebram, 1973; Schwaha, 2020d). By comparing all these characters, penetrantiid colonies and especially the stolonal connection mechanism are more similar to vesicularioideans than walkerioideans.

Two other boring ctenostome families, Terebriporidae and Spathiporidae, are also considered vesicularioideans, and also form creeping, endolithic colonies with the stolonal network being horizontally submerged in the substrate. Autozooids of spathiporids are also pedunculate (Schwaha, 2020d).

The basic colony structure and early astogeny is fairly similar between all penetrantiids. The specific stolon branching pattern along with pedunculate zooids is therefore not considered to be particularly diagnostic of species within the genus *Penetrantia*. Only the early stolonal branching pattern of the ancestrula was considered different between penetrantiids, something that could not be verified in this study (Pohowsky, 1978; Silén, 1947). Another useful character to distinguish the four recent boring bryozoan taxa is the placement of the zooids in relation to the surface. All penetrantiids have their autozooids more or less oriented vertically, whereas terebriporids and spathiporids have horizontally and oblique zooids, respectively. This difference is particularly useful in distinguishing the two pedunculate clades (Penetrantiidae and Spathiporidae) (Pohowsky, 1978; Schwaha, 2020d; Silén, 1947). Only in *Penetrantia bellardiellae* bent autozooids were observed. However, as these unique zooids appear to develop in thinner shell areas, they could be the result of limited space (Schwaha et al., 2019a).

The only other boring bryozoan taxon to have a penetrantiid-like colony morphology is the extinct genus *Haimeina,* with the sole species *H. michelini.* This species is known from the Triassic/Jurassic and had pedunculate, vertically oriented zooids. This close resemblance in colony morphology together with similarities in astogeny has led to the assumption that *Penetrantia* and *Haimeina* are closely related (Pohowsky, 1978).

Some penetrantiids show adventitious stolons that branch off from the principal stolon at random positions and do not often carry any autozooids (Pohowsky, 1978; Silén, 1947). These adventitious stolons are particularly abundant in older colonies, and their differentiation from branches of the principal stolon becomes challenging since young branches of the latter may not have formed autozooids yet. The function of these adventitious stolons is not completely clear, but since they increase the connectivity within a colony, they are considered to improve distribution of metabolites within the colony (Pohowsky, 1978).

The only penetrantiid which possesses a significantly different stolonal branching pattern is *Penetrantia irregularis* from New Zealand. Its colonies are extremely ramified with stolons branching irregularly and with autozooids being attached to one or several adventitious stolons (Gordon, 1986; Silén, 1956).

The stolons themselves are approximately circular in cross section in all investigated species, as reported for *P. concharum* and *P. parva.* Stolons of *P. densa* and *P. brevis* are laterally compressed and thus higher than wide (Silén, 1947). However, all species investigated here showed high variability in the shape and size of their stolons, which might be influenced by different substrate parameters. Shape and size of cross sectioned stolons do not seem to serve as a reliable diagnostic character.

A peculiar feature of the stolonal network in penetrantiids is the formation of tubulets, tubular extensions of the stolonal network towards the surface. (Pohowsky, 1978; Silén, 1947). Their function remains unknown, but it was speculated that tubulets may serve for substance exchange (e.g., oxygen) or to remove dissolved calcium carbonate created by the boring process. None of these ideas could be verified, but since tubulets occur exclusively in boring bryozoan taxa, they are clearly an adaptation to an endolithic lifestyle (Marcus, 1938; Pohowsky, 1978; Silén, 1947). In tubulets of *Penetrantia* sp. from Japan, a septum pierced with a single pore is present just before it terminates at the surface of the substrate, indicating that a transfer of certain molecules could be possible. However, tubulets are absent on stolons that are located close to the surface of the substrate and can be elongated on stolons that are located deeper within the substrate. This pattern suggests that tubulets might play a role in establishing the orientation of the stolonal network and the entire colony within the substrate, enabling all boring bryozoan taxa to have their colonies oriented parallel to the surface of the substrate, regardless of the orientation of the surface itself (Pohowsky, 1978).

A species-specific difference might be the distance between subsequent tubulets, although information on certain species is still scarce. Nevertheless, the differences are quite remarkable between species. For example, in *P. concharum* from Sweden, the interval can be three times longer than in *P. parva*. However, the diagnostic value is minimal, since rather large variation within a single species was observed, which might reflect different environmental conditions and substrate characteristics. Additionally, tubulets may be completely absent under certain conditions, as in *Penetrantia clionoides*. While this species was described as lacking tubulets entirely (Smyth, 1988), this study confirms their presence in this species as well as significant differences in interval length compared to *Penetrantia* sp. from Japan, a feature that might indicate different species affinities.

### Septa and pore complexes

Septa and associated pore complexes are usually situated between neighboring zooids and enable the transfer and exchange of nutrients and metabolites throughout a colony (Bobin, 1977; Mukai et al., 1997). These interzooidal septa are found along the stolonal network in all investigated penetrantiids, which renders them true kenozooids (Schack et al., 2019; Schwaha, 2020d). Each septum is pierced by a single pore, which is the case for most ctenostome bryozoans (Schwaha, 2020d). Usually, these pores are plugged and associated with specialized cells, creating a pore complex. However, such cells were not encountered in penetrantiids, in which only a single special cell persists and passes through the interzooidal pore (Bobin, 1977; Mukai et al., 1997).

### Sac zooids

Sac zooids were considered special kenozooidal heterozooids, resembling bag-like eversions of the principal stolon in *Penetrantia* (Pohowsky, 1978). These unique sac zooids often have granules incorporated into their cavity, something also reported for sac zooids of *Spathipora comma* (Bobin & Prenant, 1954). Such zooids were also reported from several other endolithic bryozoan taxa including fossil species, but also, for example, *Hypophorella expansa* (Pohowsky, 1978; Schwaha, 2020d). The function of sac zooids and their granules remains unclear (Bobin & Prenant, 1954; Pohowsky, 1978). However, the true polymorphic status of sac zooids in *Penetrantia* is called into question, since they might simply resemble modified stolons rather than specialized zooids; and granules were also observed in other parts of the stolonal network. Additionally, sac zooids represent the only non-pedunculate zooid in *Penetrantia*, which differs from the pedunculate sac zooids of *S. comma* (Bobin & Prenant, 1954). This fundamental difference between sac zooids in the genera *Penetrantia* and *Spathipora* indicates that sac zooids probably evolved independently in boring bryozoans and resemble analogous rather than homologous structures.

### Autozooid morphology

#### Gross morphology

Penetrantiids in general resemble the other two stolonate clades of ctenostomes (Walkerioidea and Vesicularioidea). In contrast, all penetrantiids are connected to the stolonal network with their lateral body wall (Schwaha, 2020d), which had led to a different definition of body axes in penetrantiids. Since the side of the orifice is referred to as frontal and the opposite as basal in most bryozoans, the body axis definition for penetrantiids was adapted accordingly (Schwaha, 2020d).

The size differences between the autozooids of penetrantiid species are substantial and provide additional information for species identification. Nevertheless, since intraspecific differences can be large as well, autozooid size should not be considered diagnostic without further corroborating characters. In this study, the largest intraspecific differences in autozooid length were observed in *Penetrantia irregularis*. Interestingly, two different size ranges for *P. irregularis* have been reported suggesting high variation in zooid dimension for this species (Gordon, 1986; Silén, 1956). Differences in zooid size between *P. concharum* from Sweden and *P.* cf. *concharum* from France might be the first indication of separate species status of the French material, especially as specimens of *P. concharum* from Sweden and Norway are very similar in size and correspond well to reported zooid dimension for the type material (Pohowsky, 1978; Silén, 1946, 1947). By comparison, zooids of *P.* cf. *concharum* from France are always significantly smaller in length and width. Less significant differences in zooid size were observed among *P. parva* from northern New Zealand and southern New Zealand and *P.* cf. *parva* from New Caledonia. The last of these has the narrowest zooids. These differences are probably the result of different environmental conditions such as changes in food availability and, particularly, water temperature, which can alter zooid size in bryozoans quite drastically (O’Dea & Okamura, 2000; Stępień et al., 2017). Such an explanation may also apply to the extremely small zooids reported for *P. taeanata* from South Korea, which is by far the smallest penetrantiid. Interestingly, autozooids of a second boring bryozoan species reported from that region, *Immergentia cheongpodensis*, are of similar size to *P. taeanata* and are smaller than most other immergentiids as well (Seo et al., 2018). Noteworthy, both species were erected based on their boring traces only (aperture shape and resin casts of colonies) with no soft body information given (Seo et al., 2018). Consequently, these two species represent ichnospecies and not true biotaxa (Bertling et al., 2006; Rosso, 2008).

Most autozooids are simple elongated tubes, sometimes curved on their anal side with the oral side being slightly bent. This condition was reported before in *Penetrantia concharum* and *P. parva* (Silén, 1946, 1947). The most obvious difference in shape of the autozooidal tubes between penetrantiids is the shape of the autozooidal basal tip. A pointed basal tip is most common among penetrantiids, especially in *P. sileni*, *P. parva*, and *P. concharum* (Silén, 1946, 1947; Soule, 1950). Only the three penetrantiids with the longest autozooids (*P. densa, P. brevis*, and *P. irregularis*) have blunt basal tips, which are sometimes even enlarged and swollen as in *P. brevis* and *P. irregularis* (Gordon, 1986; Silén, 1946, 1947, 1956). This condition could be an adaptation to limited space in the substrate, as may be the case for the bent zooids in *P. bellardiellae* which were most frequently observed in regions with limited space (Schwaha et al., 2019a).

#### Operculum

The most striking feature of all penetrantiids is the prominent operculum, which also clearly distinguishes them from all other recent boring bryozoan taxa (Pohowsky, 1978; Silén, 1946, 1947; Soule & Soule, 1969a). The operculum lies somewhat submerged or countersunk within the borehole, an additional feature thought to be of taxonomic relevance and characteristic for the family (Pohowsky, 1978). The penetrantiid operculum can give important information about species identification, although detailed information for a few species is still missing.

The size of the operculum correlates with the zooid width of the given species and is circular to elliptical in most species, which again correlates with the tubular cylindrical shape of the autozooids (Silén, 1946, 1947). The operculum of all investigated penetrantiids is strongly cuticularized, usually more so than the body wall. As it seals the borehole, it most likely has a protective function (Pohowsky, 1978; Silén, 1947). The opercula of *P. parva* from New Zealand are especially heavily cuticularized, and some have a characteristic shallow pit at the center of the frontal side. This pit is somewhat similar in position and size to the raised knob described in *P. operculata* (Soule & Soule, 1969b).

The operculum of *P. irregularis* has a peculiar groove on its frontal side that serves as a diagnostic character for this species, which has been previously used for distinguishing the two New Zealand species (Gordon, 1986; Silén, 1956). In addition, the operculum of *P. irregularis* is not as strongly cuticularized as in *P. parva* and is browner compared to the more yellow-colored opercula of *P. parva* and most other penetrantiids (Silén, 1956). The different cuticle thickness and the comb-like pits in the groove of *P. irregularis* might be the reason for different coloration. However, this unique groove, with its comb-like surface structure, is not situated on the inner basal side of the operculum as reported previously (Gordon, 1986). Rather, it is an invagination of the outer frontal surface. The least cuticularized and thinnest operculum was observed in *P.* cf. *concharum* from France, and it differs clearly from the operculum found in *P. concharum* from Sweden, which is more strongly cuticularized and is differently shaped in cross section.

The rough area on the frontal surface of the operculum in *Penetrantia* sp. from Japan looks strikingly similar to *P. clionoides* from Guam and *P. bellardiellae* from Papua New Guinea (Schwaha et al., 2019a; Smyth, 1988). In *P. clionoides*, this rough crescent-shaped area is composed of chips of calcium carbonate that are partially overlapping, giving it a “toothed” appearance (Smyth, 1988). Additionally, the opercula of all three species are at least partially composed of calcium carbonate (Schwaha et al., 2019a; Smyth, 1988). Based on the similar operculum morphology, along with very similar aperture shapes, these three species seem more closely related than to any other penetrantiid species and may form a separate clade. Intriguingly, at least four penetrantiids have their operculum partially calcified, a condition which is not reported in cheilostome bryozoans (Schwaha, 2020d). The only calcifications cheilostome opercula sometimes show are internal, cryptocystal ones, whereas the primary operculum remains uncalcified (Banta et al., 1997; Perez & Banta, 1996). Opercula were also reported from the fossil cyclostome family Eleidae. The operculum in penetrantiids is most likely not homologous to that of either of these groups, which implies that opercular structures evolved at least thrice (Taylor & Zágoršek, 2011). The underlying musculature of the operculum in penetrantiids differs from the cheilostome one as well and will be discussed later in more detail.

#### Body wall

Another unique character of penetrantiids is the presence of a double cuticle. This feature was considered apomorphic for penetrantiids and separates the family not only from the other recent boring bryozoan taxa but also from all other bryozoans (Pohowsky, 1978; Schwaha, 2020d; Silén, 1946, 1947). The function of the double cuticle is not yet fully understood, but since the exterior cuticle is the first to develop during the budding process and lines the entire borehole—even frontally of the operculum—it may be involved in the dissolution process of the substrate as the colony grows. This explanation finds support in the fact that the entire stolonal network is formed by the exterior cuticle and it is also involved in zooidal budding. In addition, the exterior cuticle is considered to play a crucial role in zooid ontogeny and during polypide recycling as new zooidal buds will develop within it. This feature is particularly prominent in gonozooids, where it seems to be involved in the formation of the brood chamber (Silén, 1947).

The interior cuticle is considered the original cuticle of the zooid and lines almost the entire borehole until the operculum; it is probably involved in the formation of the latter (Pohowsky, 1978; Silén, 1947). In the basal part, the differentiation between cuticles is difficult as they lie very tightly packed. Hence, differentiation and presence of the double cuticle is more easily observed in the frontal area (Silén, 1947). In this study, the double cuticular lining was observed in most penetrantiids, although the decalcification process together with the extraction out of the extracellular matrix of the molluscan shell may have affected the integrity of the delicate exterior cuticle in some species.

The cuticle in *Penetrantia densa* has been thought to be mineralized or to secrete calcium carbonate layers (Pohowsky, 1978). Consequently, *P. densa* would be the first ctenostome known to secrete calcium carbonate, an ability not hitherto reported for any other ctenostome (Schwaha, 2020d). However, it is not completely understood if the calcium carbonate is secreted via the cuticle itself or if the calcium-carbonate layer is a deposit of remodeled calcium carbonate previously dissolved during the boring process (Pohowsky, 1978). Possibly a process similar to that creating the apertural rims is involved. Mechanisms of calcification in penetrantiids should be investigated by future studies, as it seems increasingly likely that certain penetrantiid species are able to mineralize parts of the body or to secrete calcium carbonate.

### Soft body morphology

#### Orifice

The orifice is the external opening through which the polypide can protrude or retract. It is shifted to the anal side in penetrantiids, since the operculum seals the entire borehole and only leaves a free margin on the anal side of the zooid (Pohowsky, 1978; Silén, 1946, 1947).

A peculiar feature of penetrantiids not previously reported is a clamp-like structure formed by the vestibular wall over the free margin of the operculum. It may aid in sealing the orifice by holding the operculum in place, as this “clamp” reaches frontally of the operculum. This feature is even more prominent in gonozooids (see section on "Gonozooid morphology").

Type 1 vestibular glands have been reported in the apertural area for some penetrantiids, but no particular species was named (Soule & Soule, 1975). No penetrantiid species in this study were observed to have vestibular glands. Vestibular glands are considered typical of cheilostomes, and so the absence of these glands may point towards a ctenostome affinity (Lutaud, 1964; Schwaha et al., 2020).

The vestibular wall ends basally with a collar which shuts off the vestibulum in retracted zooids. The collar was once considered apomorphic for ctenostomes but it is also present in cheilostomes and consequently represents a plesiomorphic character of gymnolaemates (Schwaha et al., 2020). Loss or reduction of collars most likely correlates with the advent of other protective and closing mechanisms of the orifice, such as opercula (Banta, 1975; Schwaha et al., 2020). Despite being operculate, some penetrantiids have a collar, a feature previously thought to be absent in penetrantiids (Banta, 1975; Pohowsky, 1978; Silén, 1947). Indeed, *Penetrantia concharum* from Sweden has the collar reduced or lacking entirely. By contrast, we confirm a prominent, setigerous collar in three penetrantiid species. It is not entirely clear why the collar is reduced in some species, but perhaps it a consequence of being operculate. Some other ctenostomes, such as the genera *Pherusella*, *Flustrellidra*, and *Elzerina*, have evolved cuticular lips for closing the orifice and are often considered to have a reduced collar (Banta, 1975). However, a strongly modified, so-called vestibular collar is still present in these genera (Decker et al., 2021; Schwaha, 2021). The species-specific occurrence of the collar renders it also as an additional diagnostic character within penetrantiids. This feature also implies that the French *P.* cf. *concharum* and the Swedish *P. concharum* are separate species, owing to the presence or lack of a collar, respectively.

#### Lophophore

Within the tentacle sheath lies the lophophore. The number of lophophore tentacles has been considered characteristic of a species (Silén, 1946, 1947). This study shows, however, that the number of tentacles can vary within a single species, and several species share the same number, which limits its taxonomic usefulness. Although the majority of species consistently have 12 tentacles, variation in tentacle number was particularly common in *Penetrantia* cf. *concharum* from France and *P. concharum* from Sweden, something not hitherto reported in the latter (Pohowsky, 1978; Silén, 1946, 1947). Variation in tentacle number has only been reported previously for the New Zealand species *P. parva* and *P. irregularis* (Gordon, 1986; Silén, 1946, 1947, 1956). Nevertheless, although variation in tentacle number was observed in *P. parva* from New Zealand, our specimens of *P. irregularis* consistently had 13 tentacles. Interestingly, the type material of *P. irregularis* has 14 tentacles, and later investigations noted 11–13 tentacles (Gordon, 1986; Silén, 1956). Suffice to say, all observations agree that *P. irregularis* is the only penetrantiid with potentially more than 12 tentacles. The lowest number is nine tentacles and was observed in this study in *P.* cf. *concharum* from France. Since *P. irregularis* has the largest zooids of the penetrantiid we investigated and *P.* cf. *concharum* from France has the smallest, tentacle number probably correlates with polypide size, which is common in bryozoans (Winston, 1978).

#### Digestive tract

The most peculiar feature of the penetrantiid digestive tract is a proventriculus in the cardiac region. A gizzard with cuticularized or chitinous denticles has been previously described (Pohowsky, 1978; Schwaha, 2020d; Silén, 1946, 1947), but the presence of a true gizzard was cast into doubt, since denticles (as in the gizzard of vesicularioideans such as the genus *Amathia*) are absent (Markham & Ryland, 1987; Soule & Soule, 1975). Hence, the cuticularized proventriculus found in pentrantiids more closely resembles the condition found in hislopiid ctenostomes (Schwaha, 2020d).

The anus enters the tentacle sheath closer to the lophophoral base than to the vestibulum in all penetrantiids examined. This positioning corresponds closely to that in vesicularioidean ctenostomes, which usually have a lophophoral to mid-lophophoral anus (Schwaha, 2020e). The only penetrantiid possibly possessing a vestibular anus, as shown in drawings, is *Penetrantia parva* (Gordon, 1986). However, *P. parva* from both northern and southern New Zealand were found to have a mid-positioned anus in this study, since the intestine in all investigated species bends back in basal direction.

#### Brown bodies

Brown bodies are the remains of cyclic polypide degeneration events. They are either ejected from the zooid or accumulated in the proximal part. Usually, one brown body (sometimes two) develops after a degeneration-regeneration cycle, with the number of brown bodies thus correlating with the number of polypide regeneration events (Gordon, 1977). In *Penetrantia*, several brown bodies can occur within a single zooid, even in very young colonies. This observation implies either the formation of several brown bodies at a single cycling event or very rapid polypide regeneration cycles and hence multiple cycles even in young colonies. A previous study showed that autozooids of *Penetrantia densa* contain only one large brown body, whereas autozooids of *P. concharum* contain multiple but smaller brown bodies. These findings support the first hypothesis (Silén, 1946). The number of brown bodies might be species-specific, but this requires additional confirming observations.

### Autozooidial musculature

#### Apertural and tentacle sheath musculature

Some ctenostome bryozoans including vesicularioideans and walkerioideans have distinct circular and longitudinal musculature in the vestibular wall (*Mimosella*, *Amathia*) (Schwaha & Wanninger, 2018), a feature missing in all of the penetrantiids investigated here. This absence probably correlates with the presence of an operculum, as most cheilostomes also lack such musculature (Schwaha, pers. observation). Likewise, ctenostomes with bilabiate apertural closure mechanisms (Schwaha, 2021) and various other genera (e.g. Decker et al., 2020) do not show regular vestibular wall musculature.

The most unusual apertural muscle in penetrantiids is the operculum occlusor (Pohowsky, 1978; Silén, 1947; Soule & Soule, 1975). Its presence was previously used as support for a cheilostome affinity, along with the notion of the penetrantiid operculum being homologous to the cheilostome one (Soule & Soule, 1975). However, the operculum occlusor muscles in cheilostomes are now considered modified parieto-vestibular muscles, since they have a similar traversal, and parieto-vestibular muscles are otherwise absent (Schwaha et al., 2011). In penetrantiids, by contrast, the occlusors are present in addition to the parieto-vestibular musculature. Consequently, the operculum occlusor muscles in penetrantiids seem to have evolved independently from cheilostomes, although the occlusor was falsely considered to be unpaired (Silén, 1947). Whether the operculum occlusor muscles represent modified parietal muscles, as previously suggested, remains an open question, but the insertion point on the lateral body wall is different (Silén, 1947).

How closure of the operculum is realized with the insertion point of the occlusor muscle at the most oral side of the operculum remains an open question. Could it be that the occlusor muscle is actually involved in the opening process rather than in the closure of the operculum? Similar paired muscles were reported for the cheilostome genus *Cellaria*, in which a so-called divaricator muscle inserts at the proximal side of the operculum and is considered to function as an operculum opener. However, in *Cellaria*, the typical operculum occlusor muscle is also present and facilitates the closure of the operculum (Perez & Banta, 1996). In the penetrantiids, the operculum is attached to the vestibular wall, and a passive closure of the operculum by withdrawal of the vestibulum back into the cystid is conceivable. However, more data, especially comparisons to muscular systems of protruded zooids, are necessary to confirm this idea.

All penetrantiids have a set of just two parieto-vestibular muscles, in contrast to most ctenostomes, which usually have a set of four. Interestingly, ctenostomes, with a special orifice-closing mechanism (e.g., *Flustrellidra* and *Elzerina*), have two parieto-vestibular muscle bundles only. Likewise, cheilostomes do have only one pair modified as an operculum occlusor muscles, which renders the second pair of other ctenostomes redundant when a specialized closing mechanism of the orifice is present (Mukai et al., 1997; Schwaha & Wanninger, 2018; Schwaha et al., 2011). The same applies to the parieto-diaphragmatic muscle which is present as a single pair in all penetrantiids and the above mentioned taxa with two parieto-vestibular muscles (Schwaha & Wanninger, 2018). The diaphragmatic sphincter muscle consists of circular bundles and closes the atrium in all bryozoans and is homologous in all representatives (Schwaha, 2020b). The duplicature bands (formerly called parieto-vaginal bands in gymnolaemates (Schwaha et al., 2011)) are present as a set of four bundles at least in *Penetrantia concharum* from Sweden and in *P. parva* from northern New Zealand and most likely in the remaining penetrantiids as well. The organization into four duplicature bands is considered the ancestral condition in gymnolaemates (Schwaha & Wanninger, 2018; Schwaha et al., 2011, 2020).

#### Tentacle sheath musculature

Only longitudinal muscle fibers were observed, which is most common in ctenostomes (Schwaha et al., 2011). Interestingly, other ctenostome taxa, such as Victorelloidea and Walkerioidea, have diagonal muscle fibers located within their tentacle sheath, which is considered a synapomorphy for these two clades (Schwaha & Wanninger, 2018).

#### Lophophoral musculature

The circular frontal lophophoral base muscle is present either as a complete ring or as a series of individual patches (Schwaha & Wanninger, 2018). In the latter case, there are prominent muscle bundles, termed basal transversal muscles, located between the base of each tentacle. This structure was first described in the cheilostome *Cryptosula pallasiana* (Gordon, 1974). The complete or almost complete frontal muscle ring is present in several ctenostomes and is considered the ancestral condition (Schwaha & Wanninger, 2018; Schwaha et al., 2011). As it forms almost a continuous ring, the frontal lophophoral base muscle in penetrantiids resembles that in ctenostomes more than in cheilostomes. The buccal dilatators seem to be homologous among all gymnolaemates and correspond to the number of tentacles (Schwaha & Wanninger, 2018; Schwaha, 2020b).

The length of the abfrontal lophophoral base muscle is relatively short in *Penetrantia*, as is the case in other ctenostomes (e.g., *Victorella*, *Mimosella*) (Schwaha & Wanninger, 2018). The “v”-shaped muscles are inconspicuous in *Penetrantia*, and form rather patchy elements at the distal end of the abfrontal lophophoral base muscle. Such a condition is found in most other ctenostomes, and likewise does not resemble a “v” (Schwaha & Wanninger, 2018). These muscles were first described in *Hislopia malayensis*, in which they form a distinct “v” (Schwaha et al., 2011). Consequently, the term “v”-shaped muscles should be avoided, although its function still remains unknown. In *Penetrantia*, the number of f-actin-rich elements of the “v”-shaped muscle seems to vary even within the same specimen. Possibly, the small size of these elements in penetrantiids make differentiation between these f-actin-rich elements and the abfrontal lophophoral base muscle impossible.

The tentacle musculature is uniform among all bryozoans and consists of two longitudinal muscle bundles. These muscles are either smooth or striated. A gap between the tentacle muscles and the corresponding abfrontal lophophoral base muscle seems to be ancestral for all myolaemates, as this gap is not present in any phylactolaemate (Schwaha & Wanninger, 2018; Schwaha et al., 2011). Contractile elements in the tentacle tips were observed in *Hislopia malayensis* (Schwaha et al., 2011) and *Victorella pavida* (Schwaha & Wanninger, 2018), and resemble the condition found in *Penetrantia*. Such musculature is most likely associated with sperm release with the contractile elements enabling closure and/or opening of terminal tentacle pores (Schwaha & Wanninger, 2018; Silén, 1966). This hypothesis is also supported by the finding that these f-actin-rich elements were only observed in autozooids of *Penetrantia* and never in the oocyte-bearing gonozooids.

#### Digestive tract musculature

The general morphology of the digestive tract of penetrantiids is similar to other gymnolaemates (Schwaha, 2020b, 2020e). As in all myolaemates, the pharynx is lined by a myoepithelium that creates a suction-pump for food-uptake (Nielsen, 2013, Schwaha et al., 2020). Externally, it is covered by regular, cross-striated circular musculature. The esophagus shares the latter but is less prominent as in most gymnolaemates (Schwaha et al., 2011; Schwaha, 2020b). Longitudinal muscle fibers incorporated into the foregut epithelium are absent in *Penetrantia* and have been observed only in a few gymnolaemates (e.g., *Hislopia malayensis*), in which they seem to be restricted to the esophagus (Schwaha et al., 2011).

The most prominent muscle of the digestive tract in *Penetrantia* is the dense cardiac constrictor. Prominent musculature of the cardia is known from several ctenostomes and can form a cardiac constrictor, proventriculus, or a gizzard, always supplied with prominent ring musculature (Jebram, 1973; Schwaha & Wanninger, 2018). Many stolonate ctenostomes, such as vesicularioideans, have a prominent gizzard with cuticular teeth (Markham & Ryland, 1987), whereas some walkerioideans simply have a constrictor (Schwaha & Wanninger, 2018). Penetrantiids correspond well to ctenostomes that form a proventriculus. In this case, a gizzard is absent, but there is a strong cuticular lining (see section “Digestive tract”). The caecum is supplied by a mesh of very loose circular and longitudinal muscle fibers as in most gymnolaemates (Schwaha & Wanninger, 2018).

#### Retractor muscle

The retractor muscle is the most prominent muscle within each zooid and is crucial for the fast retraction of the lophophore (Mukai et al., 1997; Schwaha & Wanninger, 2018). In most myolaemates, the retractor muscle originates from the proximal or lateral body wall and attaches to the lophophore base (Schwaha & Wanninger, 2018). A similar arrangement is present in all penetrantiids. In several ctenostomes, an additional bundle also inserts at the foregut (e.g., *Flustrellidra hispida*, *Elzerina binderi*, and *Bockiella arcatumida*. (Schwaha, 2021) or in *Pherusella* sp. (Decker et al., 2020)). In some exceptional cases, additional fibers also attach to the caecum (*Aethozooides uraniae* (Schwaha et al., 2019b)). In all investigated penetrantiids, the retractor muscle bundles appear to be smooth, which is the case for most bryozoans (Schwaha et al., 2011; Schwaha, 2020b).

#### Body wall musculature

All gymnolaemates have parietal muscles which are derivatives of the body wall musculature and are crucial for polypide protrusion. Since they traverse the body cavity to insert at the frontal and basal body wall (oral and anal body wall in penetrantiids), contraction will lead to an increase in pressure of the body cavity, which then squeezes out the polypide; such a mechanism is fundamental to all gymnolaemates (Jebram, 1986; Schwaha & Wanninger, 2018; Schwaha, 2020b, 2020c). Penetrantiids do not differ in this aspect and show a series of bundles throughout their body cavity from approximately the (retracted) lophophoral base to the area of the diaphragm (Soule & Soule, 1969a). Since at least a part of the body wall needs to be flexible for this protrusion mechanism, the body wall of penetrantiids cannot be entirely fixed to its borehole, which indicates that the shape of the cavity does not exactly match the shape of the zooid.

#### Autozooidal nervous system

Most bryozoans including gymnolaemates have a set of six or, more commonly, four longitudinal neurite bundles within each tentacle. These bundles can be differentiated into at least one medio-frontal, one medio-abfrontal, and two latero-frontal neurite bundles (Gruhl & Schwaha, 2015; Schwaha, 2020b). Penetrantiids seem to lack latero-frontal neurite bundles, and a set of only two neurite bundles was detected in *Penetrantia parva* from northern New Zealand. The vesicularioid *Amathia gracilis* shares a similar situation, with two neurite bundles per tentacle. However, a pair of latero-frontal neurite bundles are present at the lophophoral base in *A. gracilis*, which merge with the mediofrontal neurite bundle to form a single bundle on the frontal side of each tentacle (Temereva & Kosevich, 2016). The mediofrontal neurite bundle of *P. parva* from northern New Zealand branches off directly from the circumoral ring nerve or ganglion whereas the abfrontal neurite bundle has intertentacular roots that laterally fuse to the median tentacle plane at each tentacle base. This condition is found in most gymnolaemate bryozoans (Schwaha, 2020b). The apparent reduction of tentacle nerves in both *A. gracilis* and *P*. *parva* could be a consequence of small zooid size.

Up to three different types of neurite bundles, medio-visceral, medio-lateral, and latero-visceral, can be associated with the innervation of the foregut (Schwaha, 2020b). In *Penetrantia parva* from northern New Zealand, only the medio-visceral and medio-lateral neurite bundles were encountered. They originate from the proximal edge of the cerebral ganglion to proceed along the foregut as found in most gymnolaemates (Schwaha, 2020b).

The apertural area is innervated by neurite bundles, which proceed along the tentacle sheath. In most gymnolaemates, the compound tentacle sheath neurite bundle ramifies in the distal part of the tentacle sheath to innervate the parieto-diaphragmatic and parieto-vestibular muscles via the vestibular wall. Some of the branches also extend more distally via the duplicature bands to extend to the body wall and to innervate the parietal musculature (Gruhl & Schwaha, 2015; Schwaha, 2020b). A similar situation was observed in *Penetrantia parva* from northern New Zealand, with the neurite bundle traversing the duplicature bands on the anal side ramifying to innervate most of the apertural musculature while the bundles on the oral bands proceeding to the most frontal part of the vestibule.

### Gonozooid morphology

#### Gross morphology

Gonozooids in all penetrantiids are considered true polymorphic heterozooids and are specialized for reproduction, especially for brooding of developing embryos (Pohowsky, 1978; Silén, 1947). Polymorphs are rather scarce among ctenostomes and largely restricted to kenozooids (Jebram, 1973; Schack et al., 2019). Among ctenostomes, gonozooids have only been reported in the genera *Spathipora* and *Immergentia*: in the fossil *S. cheethami*, the recent *S. elegans* and in *I. cheongpodensis* (Pohowsky, 1978; Seo et al., 2018). However, documentation of these “gonozooids” is rather meager, with no information about soft-body morphology. Detailed histological investigations are necessary to confirm the state of gonozooids in *S. elegans* and *I. cheongpodensis*, and to compare them to the penetrantiid form.

Zooidal shape and size of gonozooids in relation to autozooids have been held to be species-specific and useful for species discrimination within Penetrantiidae (Pohowsky, 1978; Silén, 1946; Soule & Soule, 1969a). This study confirms their diagnostic value, as they truly show unique and species-specific characteristics, particularly, the size of the brood chamber in relation to the zooidal tube of the gonozooid is very useful. In some species, the basal portion of the zooidal tube extends beyond the brood chamber, whereas in others, it has the same length as the zooidal tube (Pohowsky, 1978; Silén, 1946, 1947). The shape of the brood chamber itself and the shape of the basal extension of the zooidal tube show distinct differences between species and can be considered diagnostic as well. A second noteworthy difference is the size of the gonozooid in relation to their corresponding autozooids. In most species, gonozooids are half as long as the autozooids, with only a few species having zooids of equal size (Pohowsky, 1978). Gonozooids appear to be highly seasonal and may not be encountered regularly in colonies, particularly in younger colonies. Hence, their usefulness in discriminating penetrantiid species is sometimes limited. In addition, we still lack any information about the gonozooids of *Penentrantia taeanata* and *P. bellardiellae* (Schwaha et al., 2019a; Seo et al., 2018).

Interestingly, the size of the gonozooids in *P. parva* from northern New Zealand, southern New Zealand, as well as from New Caledonia showed some considerable differences. Size differences related to different developmental stages are unlikely, as all specimens had brooding gonozooids and thus were mature (Silén, 1947). Possibly, gonozooidal size differences could indicate different species.

Gonozooids of *Penetrantia* sp. from Spain correspond well with specimens from France and other members of the *P. concharum* complex; and since gonozooids of *P. brevis are very different* (Silén, 1947), *Penetrantia* sp. from Spain is probably closer related with *P.* cf. *concharum* from France.

#### Soft body morphology

The general size of the gonozooids is reduced in most penetrantiids, but the operculum remains of similar size. Only the gonozooids of *Penetrantia concharum* from Sweden showed smaller opercula than their corresponding autozooids, a difference not previously recorded. Differences in the apertural area were only reported for gonozooids of *P. densa,* which has strikingly oval apertures with an anal rim. Differences in the operculum structure are not present (Pohowsky, 1978).

The double cuticle of the body wall is present in all analyzed gonozooids and probably plays a crucial role in the formation and sealing of the brood chamber. To date, only the morphology of gonozooids of *P. densa* and *P. brevis* has been described in great detail (Silén, 1947). In these two species, the brood chamber is lined solely by the exterior cuticle, whereas both cuticular layers are involved in *P. concharum* from Sweden. The exterior cuticle lines the entire borehole; the interior shuts the entrance of the brood chamber with a strong cuticularized region that appears like a secondary operculum. This feature was not described previously and aids in protection of embryos within the brood chamber.

The plug of the brood chamber and separation from its zooidal tube are facilitated by the vestibular wall. Additional musculature also inserts at the vestibular wall to retract the plug of the brood chamber (see section on "Gonozooid musculature"). The plug is functionally comparable to the ooecial vesicle found in many ovicellate cheilostomes, which likewise seals the opening of the ovicell and has muscles for its retraction. However, the ooecial vesicle in cheilostomes is formed by the distal part of the maternal cystid (Ostrovsky, 2008, 2013) and not by the vestibular wall as in *P. concharum*. Since ovicells evolved several times independently within cheilostomes, their independent evolution in penetrantiids is feasible (Ostrovsky, 2008). However, the term ovicell, as previously sometimes used, should be avoided for the penetrantiid brood chamber, since doing so would suggest a homology and an erroneous link to cheilostomes (Ostrovsky, 2008). The term ovicell was previously introduced for the brood chambers in penetrantiids probably because the cuticle in *P. densa* was assumed to be partially calcified, which is a typical feature of some parts of cheilostome ovicells (Pohowsky, 1978). However, even if the exterior cuticle of *P. densa* can calcify, the two typical ovicell walls, ectooecium and entooecium, are absent (Ostrovsky, 2008; Silén, 1947).

The polypide of the gonozooids in most penetrantiids is reduced in size, and functionality of their digestive tracts has also been called into question (Pohowsky, 1978; Silén, 1947). This study confirms that the polypides are reduced in size but gives no clues about possible functions. The lophophores of four species have fewer tentacles than their corresponding autozooids, with the majority having eight tentacles (Pohowsky, 1978; Silén, 1946, 1947). *P. parva* from northern New Zealand is exceptional in that both gonozooids and autozooids can have ten tentacles, indicating that a reduction in size does not necessarily result in a reduction in tentacle number.

A lophophore and zooidal musculature are present in the gonozooids of two species investigated here (*P. concharum* and *P. parva* from northern New Zealand). The ability of gonozooids to protrude their lophophores in these species, however, could not be confirmed. Gonozooids of *P. irregularis*, at least, can do so, since several gonozooids were observed with protruded lophophores. The digestive tract is also reduced in this species, which renders feeding rather unlikely (Silén, 1956).

#### Gonozooid musculature

The musculature of the gonozooids is very similar to the corresponding autozooid. Comparable to the general reduction of the polypide, these muscular systems are also reduced. There are two major differences from the autozooidal musculature: (1) an additional paired set of apertural musculature associated with the brood chamber plug and (2) absence of tentacle tip muscles. We report the additional set of vestibular plug muscles for the first time in this study. Contraction of these muscles bends the anal portion of the vestibular wall plugging the brood chamber in a basal-oral direction and thereby opens the frontal area of the brood chamber. Similar muscles for larval release are found in cheilostome ooecial vesicles (Ostrovsky, 2008). This muscle is unpaired and less prominent in the cheilostome *Bicellariella ciliata* (Moosbrugger et al., 2012), whereas penetrantiids have a pair of prominent muscles similar in appearance to parieto-vestibular muscles.

The lack of tentacle tip musculature is probably the consequence of the absence of sperm in gonozooids. By contrast, oocytes and brooded embryos were found exclusively in gonozooids. Similar reproductive separations are present in several other bryozoans and are referred to as zooidal gonochorism, i.e., sexual dimorphism involving male and female zooids (Ostrovsky, 2013; Reed, 1991), most obviously in cyclostomes (e.g., Nekliudova et al., 2021).

#### Brooding and larvae

Ctenostomes employ a variety of different brooding mechanisms, often in the tentacle sheath or vestibular wall. The brood chambers of *Penetrantia* would be unique among ctenostomes (Ostrovsky, 2013; Reed, 1991). Unfertilized oocytes have so far only been observed in the zooidal tube of gonozooids. Fertilization is generally internal in bryozoans (Ostrovsky, 2020), which indicates that oogenesis and fertilization also occur within the zooid, close to the ovary in penetrantiids. Oviposition has not been observed in any penetrantiid, but in other bryozoans with brood chambers, oviposition is effected via a supraneural pore at the anal side of the lophophoral base (Ostrovsky & Porter, 2011), a process that would explain the necessity of a lophophore in gonozooids. Several brooding bryozoans, including a few ctenostomes, have extra-embryonic nutrition associated in the external brood chamber (Ostrovsky, 2013, 2020; Ostrovsky et al., 2009). This phenomenon implies active nutrition via an embryophore at the oeecial plug or vesicle in cheilostomes, and also a drastic increase in size of brooded progeny in general (e.g., Schwaha et al., 2019c). In *Penetrantia*, neither growth of embryos nor a specific epithelial proliferation of the vestibular plug epithelium is present. Consequently, extra-embryonic nutrition can probably be ruled out in penetrantiids.

The gonozooids seem to be capable of brooding several embryos sequentially, since both developing oocytes within the zooidal tube and embryos in the brood chamber were observed at the same time in gonozooids. No fully developed larvae or free-swimming larvae were observed, but the yolky embryos suggest lecithotrophic larvae of the coronate-type.

## Conclusion

This study increases the known geographical range of the family Penetrantiidae, as it adds several new reports, indicating a much broader distribution of certain species and, hence, the entire family. Likewise, this study adds many species of molluscs as suitable substrates and increases substrate diversity of the family, which will help future studies encountering penetrantiids.

Furthermore, this study supports the ctenostome affinity of penetrantiids, confirming the apomorphic state of several important morphological characters. The opercula and the ovicell-like brood chambers, especially, were morphologically distinct from their cheilostome counterparts. The underlying musculature of the apertural area with its unique four sets of muscles and the brood chamber plug musculature showed significant differences. Likewise, the composition of the operculum itself differs and can be calcified in some species. The lyrula-like apertural notches are clearly not part of the zooids but rather incisions in the substrate. Altogether, our observations support the idea that these structures are the result of convergent evolution and not homologous within gymnolaemates. Furthermore, the stolons are true polymorphic kenozooids, a proventriculus as well as a collar is present, as it is in many other ctenostomes. To confirm their ctenostome affinity and their closest relatives, a molecular phylogenetic reconstruction including penetrantiids and additional ctenostome taxa is required. However, there still currently are very few genetic data for both taxa. Such a study will be particularly important in resolving the systematic position of penetrantiids within the ctenostomes. A close relationship to the stolonate superfamilies Vesicularioidea and Walkerioidea seems very likely. However, the kenozooidal stolonal network might also have developed a third time independently within ctenostomes, which could explain some of the fundamental differences and would consequently separate the penetrantiids from all other ctenostomes.

Finally, our study will help in future work dealing with penetrantiid species, as it provides much new species-specific information and catalogs all vital characters for species discrimination. This work also led to the discovery of a possible new species in Japan and potential cryptic species in northern France and New Zealand. In particular, the opercula morphology seems to be a very useful character, especially when combined with information on gonozooid morphology, such as brood chamber dimensions, autozooidal and gonozooidal size ranges, as well as tentacle number. However, future molecular analysis of these potential cryptic species is required to confirm these hypotheses and to get a true picture of the diversity of this currently neglected group of bioeroders.

### Supplementary Information

Below is the link to the electronic supplementary material.

Online Resource S1—Reported substrates of each penetrantiid species. (PDF 247 KB)

Online Resource S2 – EDX analysis of *Penetrantia* sp. from Japan. (PDF 2066 KB)

## Data Availability

Data is available from the corresponding author upon reasonable request.
