# Supplementary material for: Boring bryozoans: an investigation into the endolithic bryozoan family Penetrantiidae
Source: Org Divers Evol. 2023 May 24;23(4):743–85. doi: 10.1007/s13127-023-00612-z (PMC10689564; doi:10.1007/s13127-023-00612-z)
Supplement: Supplementary file 1 — Online Resource S1—Reported substrates of each penetrantiid species. (PDF 247 KB) [file 13127_2023_612_MOESM1_ESM.pdf]

# Online resource S1

## Boring bryozoans: An investigation into the endolithic bryozoan family Penetrantiidae

Organisms Diversity & Evolution

Sebastian H. Decker[1§], Masato Hirose[2], Sarah Lemer[3], Piotr Kuklinski[4], Hamish G. Spencer[5], Abigail M. Smith[6], Thomas Schwaha[1]

[1]University of Vienna, Department of Evolutionary Biology, Schlachthausgasse 43, 1030 Vienna, Austria

[2] School of Marine Biosciences, Kitasato University, Kitasato 1-15-1, Sagamihara-Minami, Kanagawa 252-0373, Japan

[3]Marine Laboratory, UOG Station, Mangilao, Guam 96923, USA

[4]Institute of Oceanology, Polish Academy of Sciences, Sopot, Poland

[5]Department of Zoology, University of Otago, Dunedin, New Zealand

[6]Department of Marine Science, University of Otago, Dunedin, New Zealand

[§]corresponding author: sebastian.decker@univie.ac.at

**Table S1** - All reported substrates of every penetrantiid species. Predominantly molluscan shells, consequently molluscan species are listed in systematic order for each species and location.
1- Silén 1946; 2-Soule 1950; 3- Silén 1956; 4- Soule and Soule 1969; 5- Smyth 1988; 6- De Blauwe; 7- Schwaha et al. 2019; 8- Gordon 1986; 9- Voigt and Soule 1973; 10- Pohowsky 1978; * This study

| ***P. clionoides*** | | |
|---|---|---|
| **Gastropoda** | | |
| Trochoidea | | |
| *Rochia nilotica* (Linnaeus, 1767) [5] | - | **Guam** |
| *Tectus pyramis* (Born, 1778) [5] | - | **Guam** |
| *Trochus maculatus* Linnaeus, 1758 [5] | - | **Guam** |
| *Trochus ochroleucus* Gmelin, 1791 [5] | - | **Guam** |
| *Trochus* sp. [5] | - | **Guam** |
| *Turbo setosus* Gmelin, 1791 [5] | - | **Guam** |
| Hipponicoidea | | |
| *Sabia conica* (Schumacher, 1817) [5] | - | **Guam** |
| Stromboidea | | |
| *Strombus* sp [5] | - | **Guam** |
| Tonnoidea | | |
| *Annaperenna verrucosa* (Sowerby, 1825) [5] | - | **Guam** |
| *Bursa bufonia* (Gmelin, 1791) [5] | - | **Guam** |
| *Bursa* sp. [5] | - | **Guam** |
| *Casmaria ponderosa* (Gmelin, 1791) [5] | - | **Guam** |
| *Lampasopsis cruentata* (Sowerby, 1835) [5] | - | **Guam** |
| *Monoplex nicobaricus* (Röding, 1798) [5] | - | **Guam** |
| Cerithioidea | | |
| *Cerithium nodulosum* Bruguière, 1792 [5] | - | **Guam** |
| *Cerithium* sp. [5] | - | **Guam** |
| Buccinoidea | | |
| *Cantharus* sp. [5] | - | **Guam** |
| *Latirus barclayi* (Reeve, 1847) [5] | - | **Guam** |
| *Pollia undosa* (Linnaeus, 1758) [5] | - | **Guam** |
| Conoidea | | |
| *Conus eburneus* Hwass in Bruguière, 1792* | - | **Guam** |
| *Conus* sp. [5] | - | **Guam** |
| Muricoidea | | |
| *Chicoreus brunneus* (Link, 1807) [5] | - | **Guam** |
| *Chicoreus* sp. [5] | - | **Guam** |
| *Coralliophila violacea* (Kiener, 1836) [5] | - | **Guam** |
| *Drupa ricinus* (Linnaeus, 1758) [5] | - | **Guam** |
| *Drupa clathrata* (Lamarck, 1816) [5] | - | **Guam** |
| *Drupa morum* Röding, 1798 [5] | - | **Guam** |
| *Drupa rubusidaeus* Röding, 1798 [5] | - | **Guam** |
| *Drupa* sp. [5] | - | **Guam** |
| *Menathais intermedia* (Kiener, 1835) [5] | - | **Guam** |
| *Menathais tuberosa* (Röding, 1798) [5] | - | **Guam** |
| *Mancinella armigera* Link, 1807 [5] | - | **Guam** |
| *Morula uva* (Röding, 1798) [5] | - | **Guam** |
| *Morula* sp. [5] | - | **Guam** |
| *Nassa serta* (Bruguière, 1789) [5] | - | **Guam** |
| *Tenguella granulata* (Duclos, 1832) [5] | - | **Guam** |
| *Thais* sp. [5] | - | **Guam** |
| Turbinelloidea | | |
| *Vasum ceramicum* (Linnaeus, 1758) [5] | - | **Guam** |
| *Vasum turbinellus* (Linnaeus, 1758) [5] | - | **Guam** |
| *Vasum* sp. [5] | - | **Guam** |

| ***P. concharum*** | | |
|---|---|---|
| **Gastropoda** | | |
| Haliotoidea | | |
| *Haliotis cracherodii* Leach, 1814 [2] | D | **USA**/California/San Pedro |
| Trochoidea | | |
| *Tegula ligulata* (Menke, 1850) [2] | L | **USA**/California/ La Jolla |
| Littorinoidea | | |
| *Littorina littorea* (Linnaeus, 1758) [1] | - | **Sweden**/ Gullmarn |
| Buccinoidea | | |
| *Buccinum undatum* Linnaeus, 1758 [1] | - | **Sweden**/ Gullmarn |
| *Neptunea antiqua* (Linnaeus, 1758) [1] | - | **Sweden**/ Gullmarn |
| Cerithioidea | | |
| *Turritellinella tricarinata* (Brocchi, 1814) [1] | - | **Sweden**/ Gullmarn |
| **Bivalvia** | | |
| Anomioidea | | |
| *Pododesmus patelliformis* (Linnaeus, 1761) [1] | D | **Sweden**/ Gullmarn |
| Mytiloidea | | |
| *Modiolus modiolus* (Linnaeus, 1758)* | D | **Sweden**/ Kristineberg |
| *Mytilus edulis* Linnaeus, 1758 [1] | D | **Sweden**/ Gullmarn |
| *Mytilus californianus* Conrad, 1837 [2] | D | **Mexico**/Baja California |
| Pectinoidea | | |
| *Pecten maximus* (Linnaeus, 1758) [1] | D | **Sweden**/ Gullmarn |
| *Aequipecten opercularis* (Linnaeus, 1758) [1] | D | **Sweden**/ Gullmarn |
| *Pseudamussium peslutrae* (Linnaeus, 1771) [1] | D | **Sweden**/ Gullmarn |
| Arcticoidea | | |
| *Arctica islandica* (Linnaeus, 1767)* | D | **Sweden**/ Kristineberg |
| Cardioidea | | |
| *Cerastoderma edule* (Linnaeus, 1758) [1] | D | **Sweden**/ Gullmarn |
| Crassatelloidea | | |
| *Astarte sulcata* (da Costa, 1778) [1] | D | **Sweden**/ Gullmarn |
| **Crustacea** | | |
| *Balanus balanus* (Linnaeus, 1758) [1] | - | **Sweden**/ Gullmarn |
| *Semibalanus balanoides* (Linnaeus, 1767) [6] | - | **Belgium**/ Kwintebank |
| **Polychaeta** | | |
| Serpulid* | D | **Norway**/Trondheimfjord |

| ***P. cf. concharum* (France)** | | |
|---|---|---|
| **Gastropoda** | | |
| Calyptraeoidea | | |
| *Crepidula fornicata* (Linnaeus, 1758)* | L | **France**/ Roscoff |
| Buccinoidea | | |
| *Buccinum undatum* Linnaeus, 1758* | D | **France**/ Roscoff |
| **Bivalvia** | | |
| Anomioidea | | |
| *Anomia* cf. *ephippium* Linnaeus, 1758* | D | **France**/ Roscoff |
| *Pododesmus patelliformis* (Linnaeus, 1761)* | D | **France**/ Roscoff |
| Arcoidea | | |
| *Glycymeris glycymeris* (Linnaeus, 1758)* | D | **France**/ Roscoff |
| Ostreoidea | | |
| *Ostrea edulis* Linnaeus, 1758* | D | **France**/ Roscoff |
| Pectinoidea | | |
| *Mimachlamys varia* (Linnaeus, 1758)* | D | **France**/ Roscoff |
| *Pecten maximus* (Linnaeus, 1758)* | D | **France**/ Roscoff |
| Cardioidea | | |
| *Acanthocardia echinata* (Linnaeus, 1758)* | D | **France**/ Roscoff |
| *Laevicardium crassum* (Gmelin, 1791)* | D | **France**/ Roscoff |
| Mactroidea | | |
| *Lutraria lutraria* (Linnaeus, 1758)* | D | **France**/ Roscoff |
| Solenoidea | | |
| *Ensis siliqua* (Linnaeus, 1758)* | D | **France**/ Roscoff |
| *Solen capensis P. Fischer, 1881** | D | **France**/ Roscoff |
| Veneroidea | | |
| *Callista chione* (Linnaeus, 1758)* | D | **France**/ Roscoff |
| *Polititapes rhomboides* (Pennant, 1777)* | D | **France**/ Roscoff |
| *Venus verrucosa* Linnaeus, 1758* | D | **France**/ Roscoff |

| ***Penetrantia sp.* (Iberia)** | | |
|---|---|---|
| **Bivalvia** - Anomioidea | | |
| *Anomia* cf. *ephippium* Linnaeus, 1758* | D | |

| ***P. parva*** | | |
|---|---|---|
| **Gastropoda** | | |
| Calyptraeoidea | | |
| *Sigapatella tenuis* (Gray, 1868)* | D | **New Zealand**/ South |
| Buccinoidea | | |
| *Cominella adspersa* (Bruguière, 1789)* | D | **New Zealand**/ North |
| *Cominella maculosa* (Martyn, 1784) [1] | L | **New Zealand**/ North |
| Conoidea | | |
| *Conus ebraeus* Linnaeus, 1758 [4] | - | **Hawai**/ Pokai Bay |
| **Bivalvia** | | |
| Arcoidea | | |
| *Glycymeris modesta* (Angas, 1879)* | D | **New Zealand**/ North |
| Pectinoidea | | |
| *Pecten novaezelandiae* Reeve, 1852* | D | **New Zealand**/ South |
| Ostreoidea | | |
| *Ostrea chilensis* Küster, 1844* | D | **New Zealand**/ South |
| Hiatelloidea | | |
| *Panopea smithae* Powell, 1950* | D | **New Zealand**/ North |
| Veneroidea | | |
| *Austrovenus stutchburyi* (W. Wood, 1828)* | D | |
| *Tawera phenax* (Finlay, 1930)* | D | **New Zealand**/ North |

| ***P.* cf. *parva*** | | |
|---|---|---|
| **Gastropoda** - Trochoidea | | |
| *Turbo petholatus* Linnaeus, 1758* | H | **New Caledonia** |

| ***P. irregularis*** | | |
|---|---|---|
| **Gastropoda** | | |
| Cerithioidea | | |
| *Maoricolpus roseus* (Quoy & Gaimard, 1834) [8] | L | **New Zealand/ North** |
| Buccinoidea | | |
| *Argobuccinum pustulosum* ([Lightfoot], 1786)* | D | **New Zealand**/ South |
| **Bivalvia** | | |
| Mytiloidea | | |
| *Perna canaliculus* (Gmelin, 1791) [3] | L | **New Zealand**/ Little Papanui |
| Hiatelloidea | | |
| *Panopea smithae* Powell, 1950* | D | **New Zealand**/ South |

| ***P. densa*** | | |
|---|---|---|
| **Gastropoda** | | |
| Lottioidea | | |
| *Lottia scabra* (Gould, 1846) [2] | L | **USA**/Californi/ Point Fermin |
| Haliotoidea | | |
| *Haliotis cracherodii* Leach, 1814 [2] | D | **USA**/California/San Pedro |
| Fissurelloidea | | |
| *Fissurella volcano* Reeve, 1849 [2] | L | **USA**/Californi/ Point Fermin |
| Trochoidea | | |
| *Tegula ligulata* (Menke, 1850) [2] | D | **USA**/California/ La Jolla |
| Buccinoidea | | |
| *Burnupena catarrhacta* (Gmelin, 1791) [1] | L | **South Africa**/ Good Hope |
| *Burnupena cincta limbosa* (Lamarck, 1822) [1] | L | **South Africa**/ Port Nolloth |
| *Fusinus* sp. [1] | L | **Pananma** |
| Muricoidea | | |
| *Acanthinucella spirata* (Blainville, 1832) [2] | D | **USA**/California/San Pedro |
| Olivellinae | | |
| *Callianax biplicata* (Sowerby, 1825) [2] | D | **USA**/California/ La Jolla |

| ***P. brevis*** | | |
|---|---|---|
| **Gastropoda** | | |
| Trochoidea | | |
| *Gibbula candei* (d'Orbigny, 1840) [1] | L | **Madeira**/ Funchal Bay |
| *Steromphala divaricata* (Linnaeus, 1758) [1] | L | **Spain**/ Ibiza |
| Buccinoidea | | |
| *Pisania striata* (Gmelin, 1791) [1] | L | **Spain**/ Ibiza |
| *Cantharus* sp. [1] | - | **Spain**/ Ibiza |

| ***P. sileni*** | | |
|---|---|---|
| **Scaphopoda** | | |
| *Dentalium vallicolens* Raymond, 1904 [2] | - | **Mexico**/ San Benito Islands |

| ***P. operculata*** | | |
|---|---|---|
| **Gastropoda** - Conoidea | | |
| *Conus striatus* Linnaeus, 1758 [4] | - | **Hawai**/ Haena Bay |

| ***P. taeanata*** | | |
|---|---|---|
| commonly found in bivalves, particularly oyster shells, but also noted in a few gastropod shells | | **Korea**/ Taean Coast |

| ***P. bellardiellae*** | | |
|---|---|---|
| **Gastropoda** | | |
| *Bellardiella crassilabris* Möllendorff, 1899 [7] | H | **Papua New Guinea** |

| ***Penetrantia* sp. Japan** | | |
|---|---|---|
| **Gastropoda** | | |
| Trochoidea | | |
| *Tegula rugata* (A. Gould, 1861)* | L | **Japan**/ Yoshihama Bay |
| Buccinoidea | | |
| *Japeuthria ferrea* (Reeve, 1847)* | L | **Japan**/ Sagami Bay |
| Muricoidea | | |
| *Reishia clavigera* (Küster, 1860)* | L | **Japan**/ Sagami Bay |

| ***P. gosaviensis* †** | | |
|---|---|---|
| **Gastropoda** - Acteonelloidea † | | |
| *Acteonella* d'Orbigny, 1842 † [9] | | **Austria**/ Gosau |

| ***P. soulei* †** | | |
|---|---|---|
| **Gastropoda** - Buccinoidea | | |
| *Neptunea contraria* (Linnaeus, 1771) [10] | | **Netherlands**/ Kaloot |

**1**-Silén 1946 **2**-Soule 1950 **3**- Silén 1956 **4**-Soule and Soule 1969 **5**- Smyth 1988 **6**- De Blauwe **7**- Schwaha et al. 2019 **8**-Gordon 1986 **9**- Voigt and Soule 1973 **10**- Pohowsky 1978 * This study
