# Supplementary material for: Boring bryozoans: an investigation into the endolithic bryozoan family Penetrantiidae
Source: Org Divers Evol. 2023 May 24;23(4):743–85. doi: 10.1007/s13127-023-00612-z (PMC10689564; doi:10.1007/s13127-023-00612-z)
Supplement: Supplementary file 2 — Online Resource S2 – EDX analysis of Penetrantia sp. from Japan. (PDF 2066 KB) [file 13127_2023_612_MOESM2_ESM.pdf]

Online resource S2

Boring bryozoans: An investigation into the endolithic bryozoan family Penetrantiidae

Organisms Diversity & Evolution

Sebastian H. Decker[1§], Masato Hirose[2], Sarah Lemer[3], Piotr Kuklinski[4], Hamish G. Spencer[5], Abigail M. Smith[6], Thomas Schwaha[1]

[1]University of Vienna, Department of Evolutionary Biology, Schlachthausgasse 43, 1030 Vienna, Austria
[2] School of Marine Biosciences, Kitasato University, Kitasato 1-15-1, Sagamihara-Minami, Kanagawa 252-0373, Japan
[3]Marine Laboratory, UOG Station, Mangilao, Guam 96923, USA
[4]Institute of Oceanology, Polish Academy of Sciences, Sopot, Poland
[5]Department of Zoology, University of Otago, Dunedin, New Zealand
[6]Department of Marine Science, University of Otago, Dunedin, New Zealand
[§]corresponding author: sebastian.decker@univie.ac.at

Penetrantia

Author: CIUS EM - Lab

Creation: 05/31/2021 9:41:03 AM

Sample Name: JT20

JT20

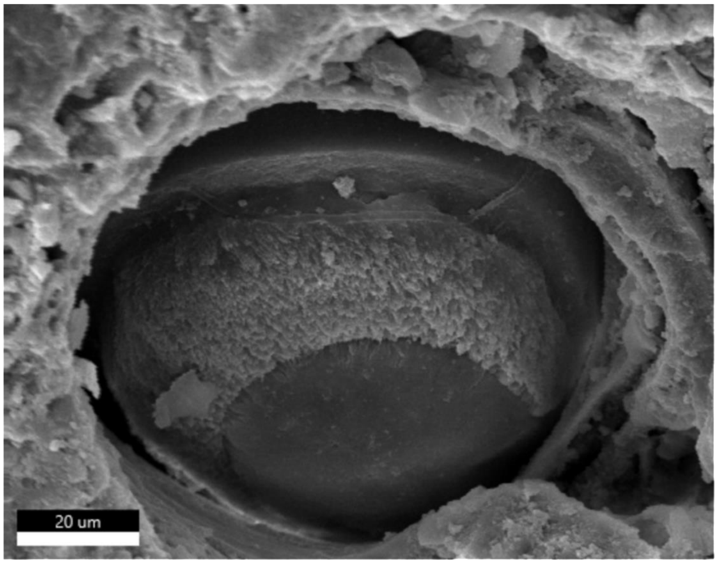

Image

Live Map 1

ElementOverlay

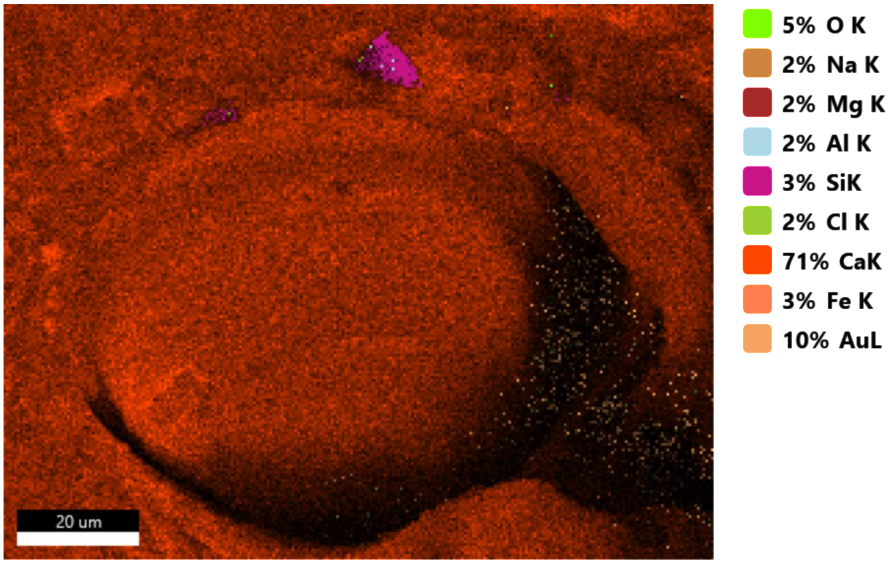

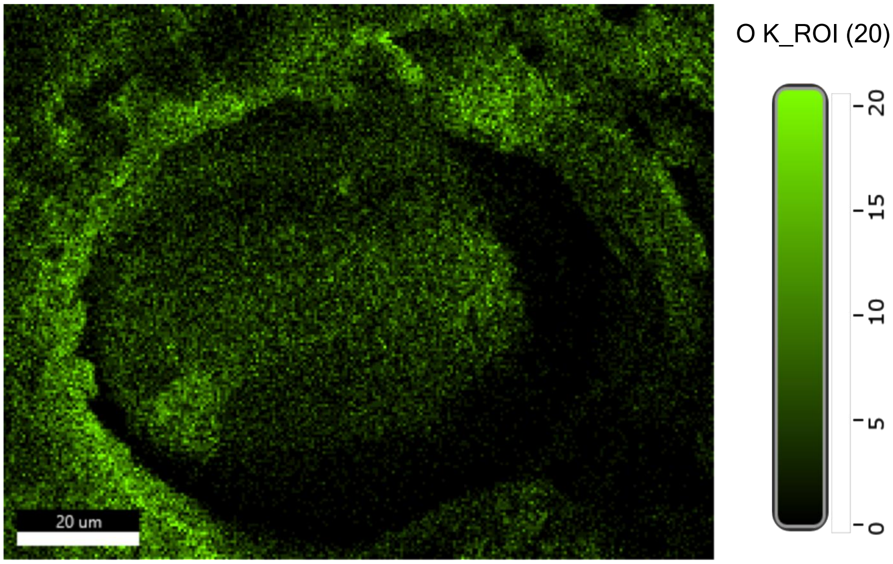

O K_ROI (20)

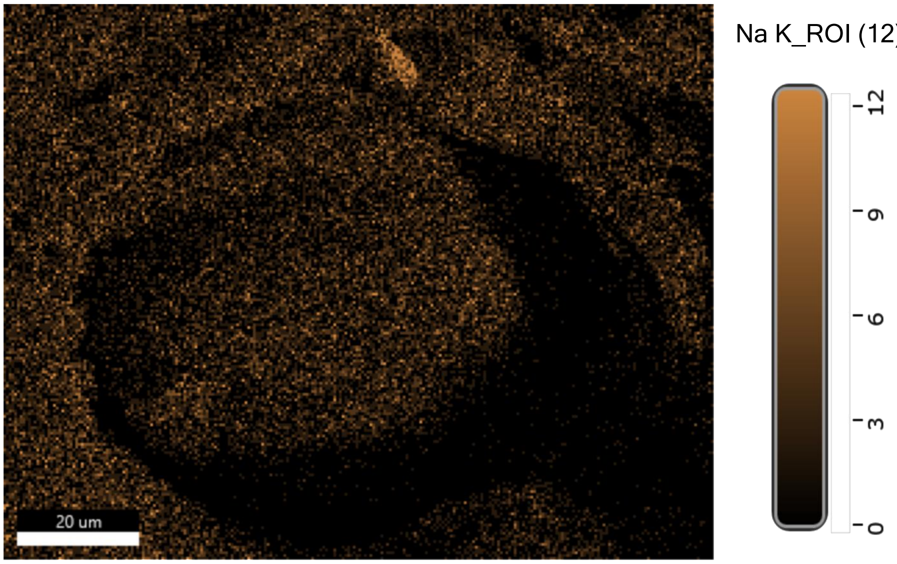

Na K_ROI (12)

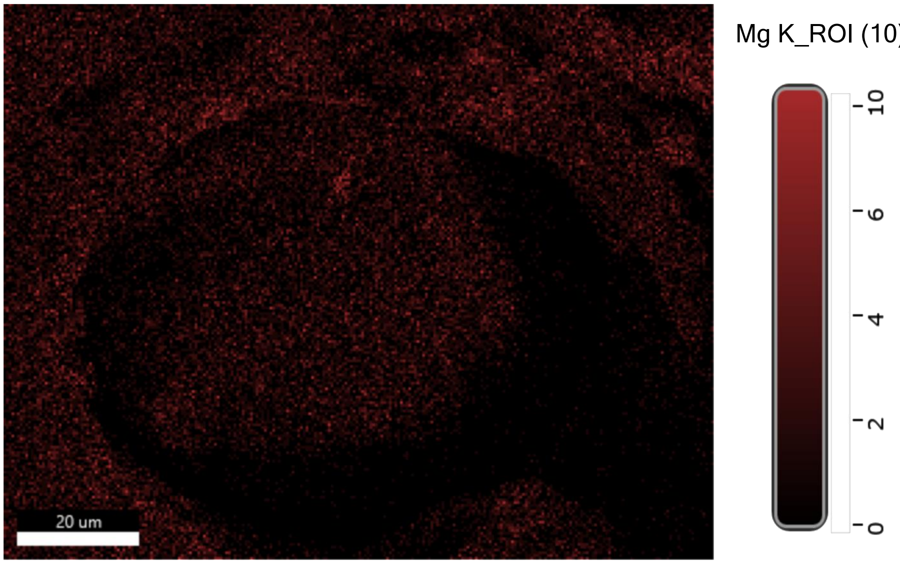

Mg K_ROI (10)

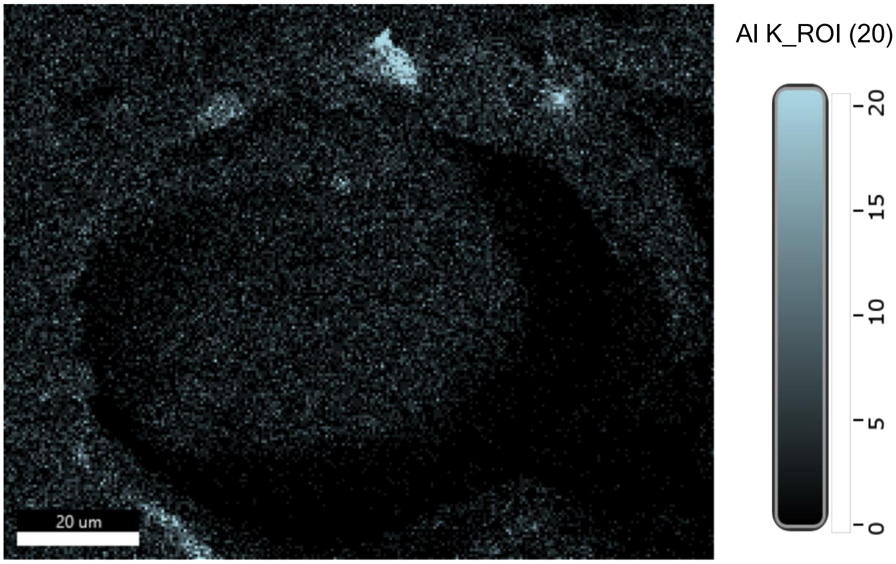

Al K_ROI (20)

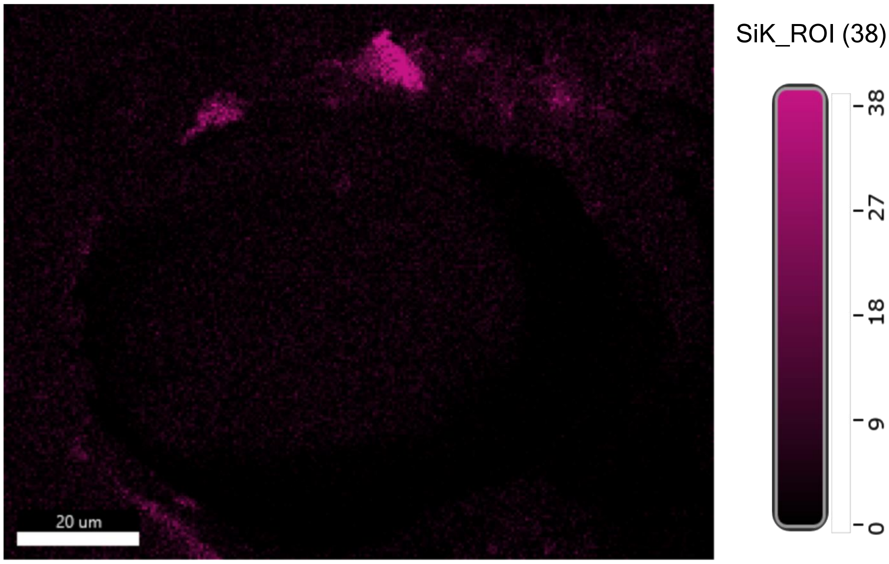

SiK_ROI (38)

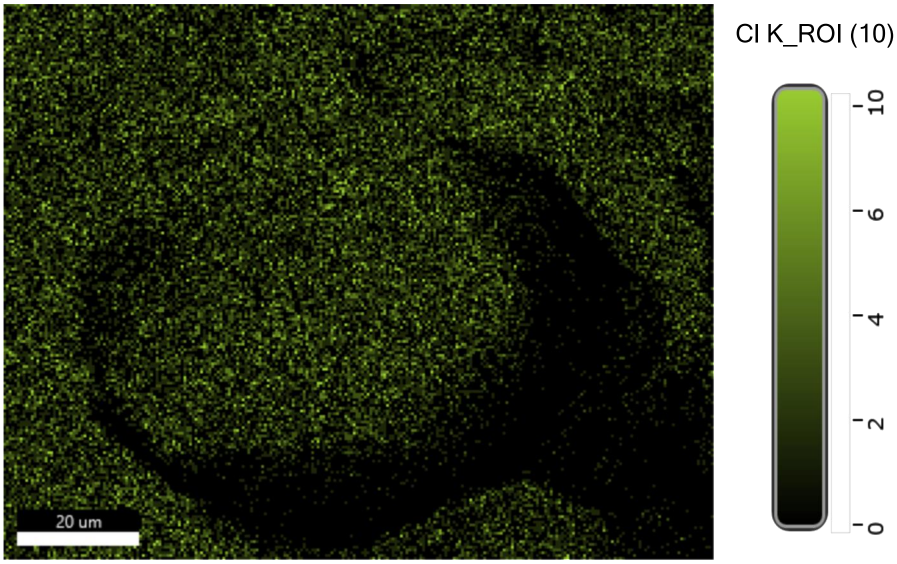

Cl K_ROI (10)

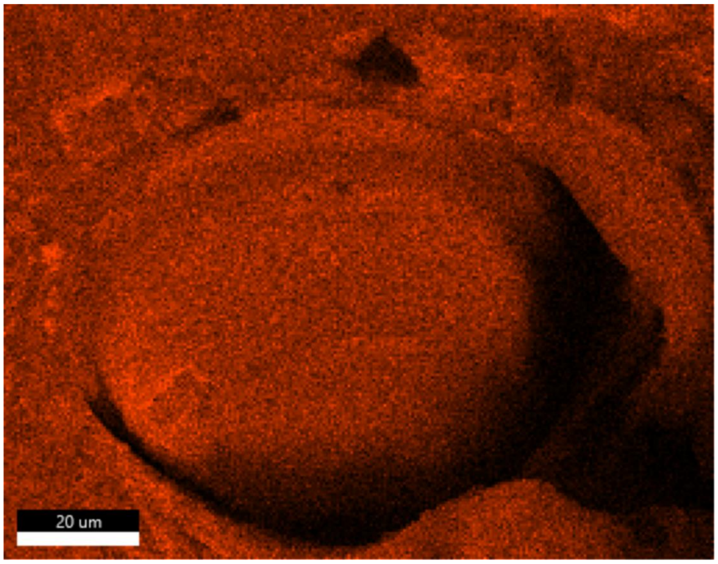

CaK_ROI (71)

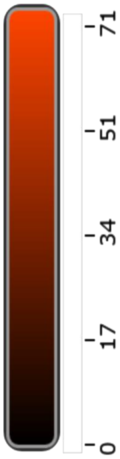

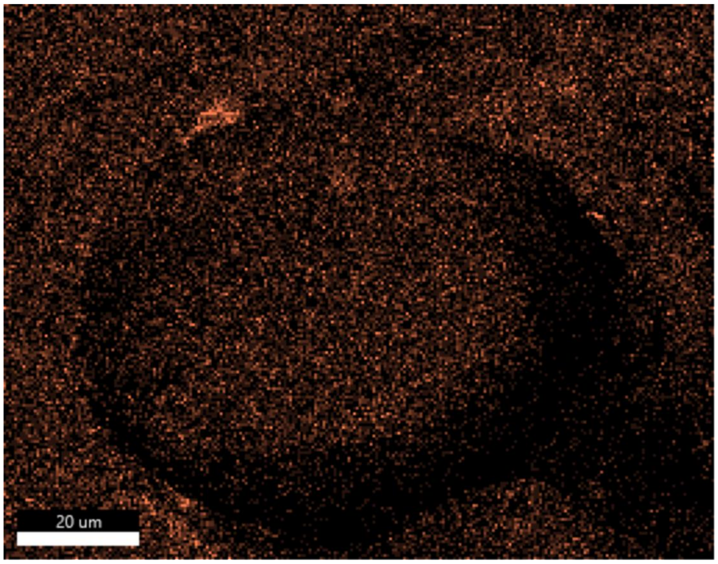

Fe K_ROI (15)

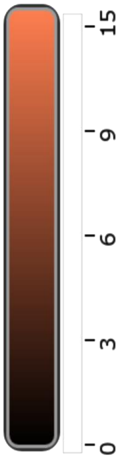

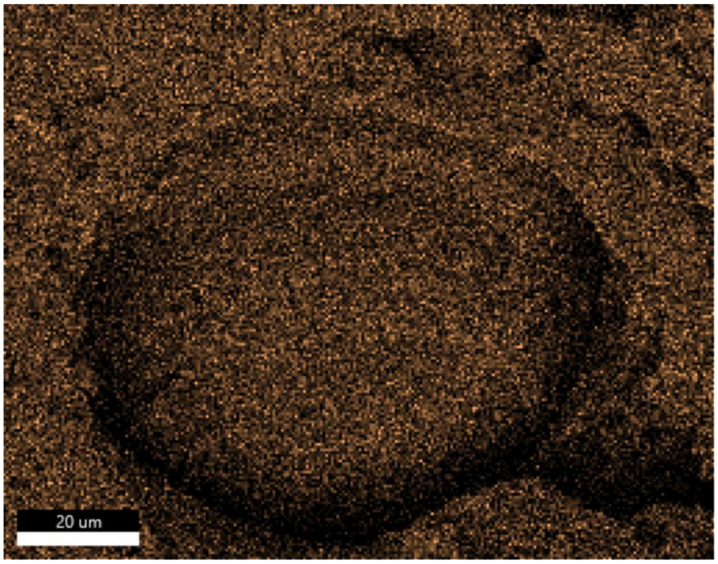

AuL_ROI (14)

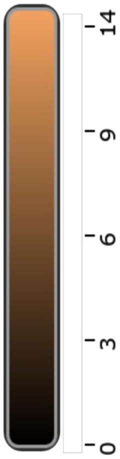

kV: 25	Mag: 1100	Takeoff: 35	Live Time(s): 227.8	Amp Time(µs): 3.84	Resolution:(eV) 126.8

Sum Spectrum

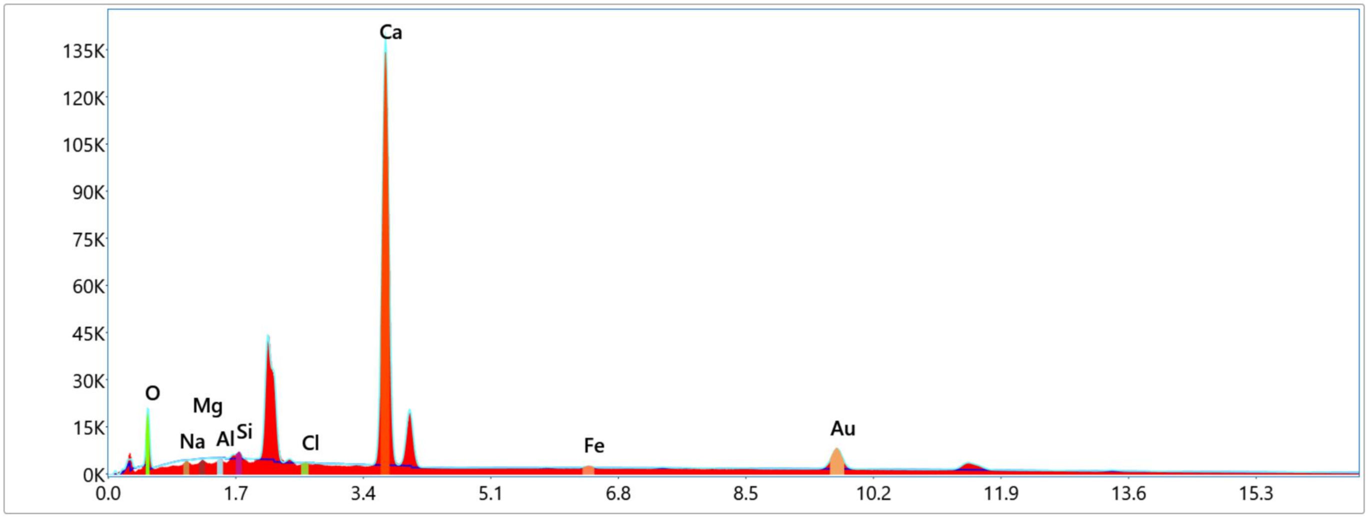

Det: Octane Plus

eZAF Quant Result - Analysis Uncertainty: 6.15 %

Element	Weight %	MDL	Atomic %	Net Int.	Error %	R	A	F
O K	34.00	0.30	65.32	436.08	10.87	0.8209	0.0410	1.0000
Na K	0.00	0.00	0.00	0.00	99.99	0.8380	0.1332	1.0043
Mg K	0.00	0.00	0.00	0.00	99.99	0.8435	0.2213	1.0069
Al K	0.00	0.00	0.00	0.00	99.99	0.8487	0.3303	1.0112
Si K	0.32	0.03	0.35	68.70	9.71	0.8536	0.4483	1.0168
Cl K	0.00	0.00	0.00	0.63	88.52	0.8675	0.5581	1.0318
Ca K	39.22	0.04	30.08	6539.88	3.77	0.8805	0.7262	1.0193
Fe K	0.29	0.08	0.16	30.15	11.90	0.9045	0.8619	1.1009
Au L	26.17	0.45	4.08	524.41	3.42	0.9405	0.9644	1.0353
